# Epidemiological study of pediatric nutritional deficiencies: an analysis from the global burden of disease study 2019

**DOI:** 10.1186/s12937-024-00945-1

**Published:** 2024-04-18

**Authors:** Chenhan Mao, Zhuyang Shen, Dan Long, Min Liu, Xiaojin Xu, Xin Gao, Yan Lin, Xindong Wang

**Affiliations:** 1 Affiliated Hospital of Integrated Traditional Chinese and Western Medicine, Nanjing University of Chinese Medicine, Nanjing, Jiangsu China; 2 Jiangsu Province Academy of Traditional Chinese Medicine, Nanjing, Jiangsu China; 3 The First Hospital of Hunan University of Chinese Medicine, Changsha, Hunan China

**Keywords:** Nutritional deficiencies, Children, Global burden of Disease (GBD), Epidemiology, Joinpoint regression analysis

## Abstract

**Background:**

Nutritional deficiencies (ND) continue to threaten the lives of millions of people around the world, with children being the worst hit. Nevertheless, no systematic study of the epidemiological features of child ND has been conducted so far. Therefore, we aimed to comprehensively assess the burden of pediatric ND.

**Methods:**

We analyzed data on pediatric ND between 1990 and 2019 from the Global Burden of Disease study (GBD) 2019 at the global, regional, and national levels. In addition, joinpoint regression models were used to assess temporal trends.

**Results:**

In 2019, the number of prevalent cases of childhood malnutrition increased to 435,071,628 globally. The global age-standardized incidence, prevalence, and DALY rates showed an increasing trend between 1990 and 2019. Meanwhile, the burden of child malnutrition was negatively correlated with sociodemographic index (SDI). Asia and Africa still carried the heaviest burden. The burden and trends of child malnutrition varied considerably across countries and regions. At the age level, we found that malnutrition was significantly more prevalent among children < 5 years of age.

**Conclusion:**

Pediatric ND remains a major public health challenge, especially in areas with low SDI. Therefore, primary healthcare services in developing countries should be improved, and effective measures, such as enhanced pre-school education, strengthened nutritional support, and early and aggressive treatment, need to be developed.

**Supplementary Information:**

The online version contains supplementary material available at 10.1186/s12937-024-00945-1.

## Introduction

The Sustainable Development Goals (SDGs) released by the United Nations General Assembly in 2015 aim to eradicate all forms of malnutrition by 2030. These SDGs also seek to achieve the international goals related to stunting and wasting in children under 5 years of age by 2025. Despite some progress on these goals, nutritional deficiencies (ND) remain widespread in poor countries [1]. Malnutrition has multiple risk factors, thus leading to differences in distribution, causes and impacts. These risk factors include poverty, low levels of education and medical awareness, food insecurity, limited access to healthcare, environmental pollution, living in areas with a high burden of infectious diseases, and inappropriate breastfeeding [2–8].

It is worrying that malnutrition continues to threaten the lives of millions of people around the world, with children being the most affected [9–12]. A recent report published by the World Health Organization (WHO) in 2020 found that malnutrition was responsible for over half of all child deaths in developing countries in the 1990s [13]. Vitamin A deficiency is still prevalent in South Asia and sub-Saharan Africa [14]. Thirteen countries have a prevalence of iodine deficiency in preschool children of more than 50% [15]. In some countries in sub-Saharan Africa, the prevalence of iron deficiency and iron-deficiency anaemia exceeds 60% in the paediatric population [16]. Strikingly, 50% of pediatric morbidity and mortality is attributed to malnutrition in sub-Saharan Africa [17]. Childhood malnutrition, which mainly includes protein-energy malnutrition and micronutrient deficiencies, not only affects children’s growth and development but may also cause the development of many chronic diseases in adulthood [18–21].

As is well known, there are considerable regional variations in the nutritional status of children. Unfortunately, the epidemiology of childhood malnutrition in different countries and regions has not been systematically assessed so far. Here, we assessed the burden and trends caused by pediatric malnutrition, including incidence, prevalence, and disability-adjusted life year (DALY), at global, regional, and national levels between 1990 and 2019. We aim to raise public awareness of the prevention and treatment of child malnutrition and to inform health policymaking.

## Materials and methods

### Data sources

The GBD 2019 is a large database researched by a multi-country cooperation that covers all WHO member countries. It provides a comprehensive assessment of health losses due to 369 diseases and injuries and 87 risk factors in 204 countries and territories worldwide between 1990 and 2019 [22–24]. The GBD study incorporates data from many various data sources, including censuses, household surveys, vital statistics, disease registries, disease notifications, health service use, websites of governments and international organizations and other sources. All data for this study were obtained from the Global Burden of Disease Study (GBD) 2019 database via the official website https://vizhub.healthdata.org/gbd-results/. The GBD 2019 study used DisMod-MR 2.1, a Bayesian meta-regression method designed to overcome the limitations of descriptive epidemiological data, such as inconsistency, missing data, as well as any large variation in methodology between data sources. A detailed description of the raw data and general methods to generate the GBD estimates have been described in detail in previous publications [25, 26] and can be found in Supplementary Material.

Malnutrition in the GBD database comprises five subtypes: iron deficiency, iodine deficiency, vitamin A deficiency, protein-energy malnutrition, and other nutrient deficiencies. Malnutrition was defined based on the 10th revision International Classification of Diseases (ICD) codes for nutritional deficiencies, stratified based on protein-energy malnutrition (ICD-10 codes E40-E46.9, E64.0), iodine deficiencies (E00-E02), vitamin A deficiencies (E50-E50.9, E64.1), dietary iron deficiencies (D50-D50.9), and other nutritional deficiencies such as vitamin deficiency anaemias, thiamine, niacin, vitamin D, vitamin C, calcium, selenium, and folate deficiency (D51-D53.9, E51-E61.9, E63-E64, E64.2-E64.9) [25].

The GBD 2019 study defines sociodemographic index (SDI) as a composite index ranging from 0 to 1, representing average incomes per capita, education level, and fertility rates. SDI value of 0 indicates that the region has the lowest level of theoretical development related to health outcomes, while SDI value of 1 indicates that the region is ranked the highest [25]. This value was then applied to categorize countries into low (SDI < 0.45), low-middle (SDI ≥ 0.45 and < 0.61), middle (SDI ≥ 0.61 and < 0.69), high-middle (SDI ≥ 0.69 and < 0.80), and high (SDI ≥ 0.80) SDI bands. Countries and territories were grouped into 21 regions based on epidemiological similarity and geographical proximity [27]. We collected data from the GBD database on malnutrition from 1990 to 2019 globally, in 21 GBD regions, and 204 countries and territories stratified by sex and age group (< 5, 5–9, 10–14).

### Statistical analysis

This study was conducted on children under 15 years of age and analyzed the burden and trend of child malnutrition by age, sex, year, and location. In the GBD study, uncertainty in the estimates, the main considerations are uncertainty due to data sources, modeling uncertainty, data errors, and data manipulation. The uncertainty of estimates was calculated by creating 1000 values for each estimate of the burden and aggregating across causes and locations at the level of each of the 1000 values for all intermediate steps in the calculation. The lower and upper bounds of the 95% uncertainty intervals (UIs) were the 25th and 975th values of the ordered 1000 values. Significant differences were established if 975 or more of the ordered 1000 values of difference were on either side of zero. Absolute cases and age-standardized rates (ASRs) of incidence, prevalence, and DALY were reported to measure the burden of ND among children. DALYs are the sum of years of life lost and years lived with disability. ASRs, calculated using the GBD world standard population, remove the effect of differences in age distribution across countries, regions, or historical periods to facilitate data comparisons.

In addition, joinpoint regression analyses were used to assess temporal trends in child malnutrition. The Joinpoint software (version 4.9.1.0; National Cancer Institute, Rockville, MD, US) was used to understand temporal trends in a structured way and to test which trends between joinpoints were statistically significant [28]. The Joinpoint software fitted a log-linear model using ASRs under a Poisson distribution. Monte Carlo permutation method was used to determine the number of connected points and corresponding *P*-value. Bayesian information criterion (BIC) was employed to test the model’s goodness of fit [29]. We specified a minimum of zero and a maximum of five joinpoints in the model. Average Annual Percentage Change (AAPC), Annual Percentage Change (APC), and the corresponding 95% confidence intervals (CIs) were calculated for this study. APC/AAPC > 0 indicated that the rates increased year by year, and APC/AAPC < 0 represented that the rates decreased year by year during the segment.

Moreover, R 4.3.1 was used for data analysis and plotting in this study. *P* < 0.05 was considered statistically significant.

## Results

### Global burden and trends

**Fig. 1:**
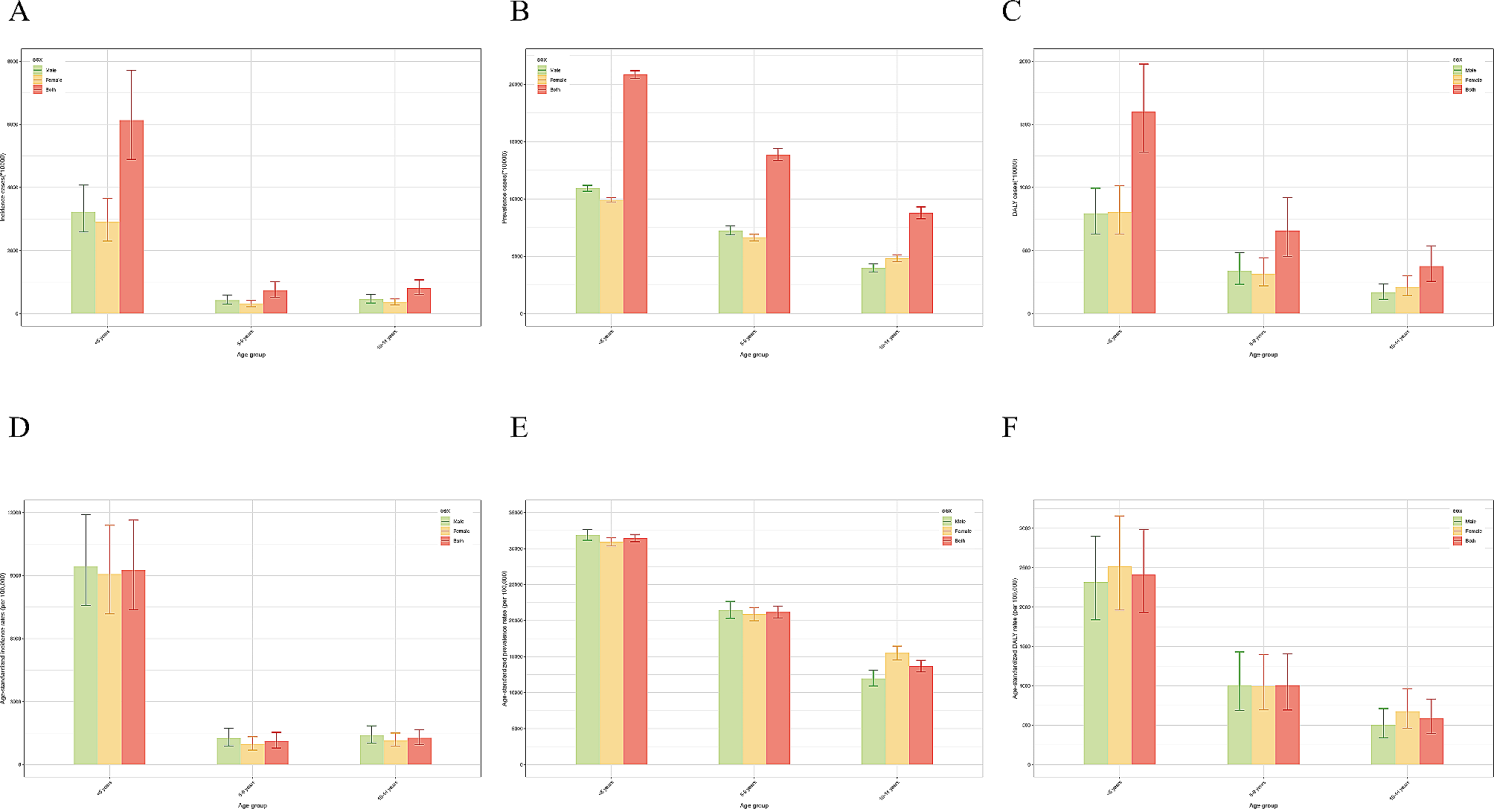
Incidence, prevalence and DALY of children with nutritional deficiency in different age groups in 2019. (**A**) incident cases; (**B**) prevalent cases; (**C**) DALYs; (**D**) incidence; (**E**) prevalence; (**F**) DALY rate

**Fig. 2:**
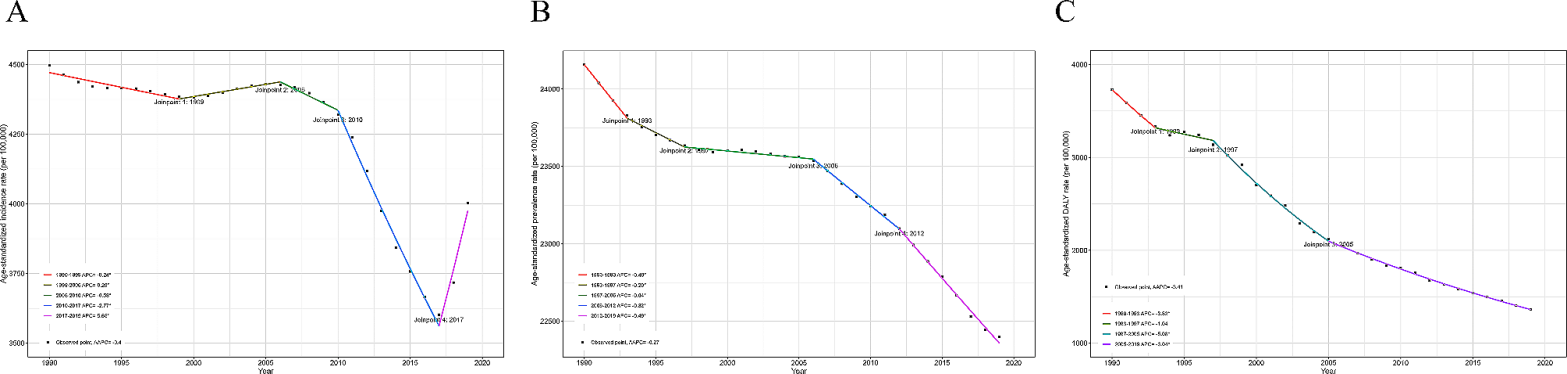
Joinpoint regression analysis of ASRs for nutritional deficiency in children from 1990 to 2019. (**A**) incidence; (**B**) prevalence; (**C**) DALY rate

In 2019, 52,330,896 (95% UI: 48,025,169 to 57,716,774) new cases and 26,339,578 (20,235,568 to 33,595,123) DALYs of childhood malnutrition were reported, with 435,071,628 (425,910,405 to 444,749,386) prevalent cases (Tables 1, 2 and 3). In the context of gender, the prevalence and DALYs of childhood malnutrition were more considerable among girls than boys, while the opposite was true for incidence. Furthermore, among all age groups, children under 5 years had the highest cases and ASRs of incidence, prevalence, and DALYs (Fig. 1 and Supplementary Table 1). Over the past three decades, the global age-standardized incidence, prevalence, and DALY rates of childhood malnutrition have declined (Fig. 2 and Supplementary Table 2). More specifically, as shown in Fig. 2, the global age-standardized DALY rate has significantly decreased since 1990, and the most notable declines were observed between 1997 and 2005 (APC = -5.08% (95% CI: -5.37% to -4.78%), *P* < 0.05). However, ASIR declined most significantly between 2010 and 2017 (APC = -2.77% (95% CI: -2.93% to -2.62%), *P* < 0.05) and then has been consistently increasing in the past 2 years. Similarly, the ASPR of childhood malnutrition decreased consistently with different APCs, with the most significant decline occurring between 1990 and 1993 (APC = -0.48% (95% CI: -0.59% to -0.37%), *P* < 0.05).

**Table 1:** Incidence number and ASR of nutritional deficiencies among children in 1990 and 2019, and AAPC of ASR from 1990 to 2019

Incidence	1990		2019		1990–2019
	Cases NO.(95%UI)	ASR/100,000 (95% CI)	Cases NO.(95%UI)	ASR/100,000 (95% CI)	AAPC% (95%CI)
Global	48554505.95 (45694002.85 to 52168822.4)	4496.41 (3431.97 to 5966.41)	52330896.46 (48025168.54 to 57716773.7)	4003.24 (3139.28 to 5101.4)	-0.4 (-0.51 to -0.3)
Sex					
Boy	26416220.36 (24758966.86 to 28460924.74)	4569.06 (3531.95 to 5969.36)	28887791.7 (26363871.47 to 32068822.98)	4145.03 (3254.90 to 5314.40)	-0.36 (-0.46 to -0.25)
Girl	22138285.58 (20920822.13 to 23694761.26)	4419.80 (3322.51 to 5967.01)	23443104.75 (21666997.69 to 25716158.36)	3851.90 (3008.93 to 4893.96)	-0.48 (-0.6 to -0.36)
SDI					
High SDI	1492630.15 (1279789.94 to 1765155.65)	1175.76 (892.18 to 1573.99)	1471405.09 (1223400.11 to 1786140.06)	1201.98 (891.55 to 1656.19)	0.06 (-0.07 to 0.18)
High-middle SDI	4657097.08 (4231047.21 to 5206690.89)	1543.97 (1387.34 to 1740.7)	4744068.72 (4198433.8 to 5437811.82)	1963.78 (1723.9 to 2269.45)	0.81 (0.71 to 0.91)
Middle SDI	12082296.62 (11166181.41 to 13245258.34)	2066.15 (1892.13 to 2280.84)	13681028.33 (12340124.58 to 15413352.65)	2530.32 (2275.62 to 2849.57)	0.67 (0.53 to 0.81)
Low-middle SDI	20212480.05 (19235958.75 to 21363149.62)	4283.8 (4040.33 to 4585.04)	17775411.52 (16379434.83 to 19526408.69)	3508.87 (3228.13 to 3859.1)	-0.7 (-0.89 to -0.51)
Low SDI	10092393.4 (9682574.55 to 10562167.38)	3813.05 (3615.6 to 4047.5)	14636987.97 (13841222.94 to 15607947.71)	3027.57 (2835.82 to 3260.86)	-0.79 (-0.89 to -0.7)
GBD regions					
Andean Latin America	99685.48 (94043.87 to 106285.47)	921.38 (736.56 to 1167.31)	90176.82 (82878.3 to 99289.97)	678.36 (559.86 to 831.58)	-1.12 (-1.32 to -0.93)
Australasia	44128.96 (40475.76 to 48532.6)	1049.17 (923.76 to 1207.8)	49978.41 (45785.07 to 55536.76)	1010.49 (884.53 to 1184.09)	-0.16 (-0.79 to 0.48)
Caribbean	109478.8 (101988.72 to 118915.32)	2183.07 (1602.06 to 2900.99)	100393.17 (91919.79 to 111553.14)	1767.96 (1306.79 to 2360.04)	-0.74 (-0.89 to -0.6)
Central Asia	388234.47 (368914.74 to 409993.42)	2180.17 (1759.53 to 2727.85)	402101.16 (379080.06 to 429956.12)	2109.37 (1695.28 to 2690.47)	-0.11 (-0.27 to 0.05)
Central Europe	305636.1 (271657.75 to 347183.25)	1982.61 (1449.9 to 2749.26)	198164.63 (174732.37 to 229591.41)	2039.63 (1431.15 to 2940.74)	0.11 (-0.04 to 0.27)
Central Latin America	714708.42 (649350.51 to 797101.62)	1798.74 (1351.7 to 2401.61)	724756.32 (639605.28 to 834152.63)	1524.52 (1206.31 to 1948.87)	-0.61 (-0.66 to -0.56)
Central Sub-Saharan Africa	855762.9 (806887.97 to 904989.58)	5350.14 (4160.88 to 6853.47)	1433299.05 (1261327.03 to 1,612,817)	3699.31 (2864.18 to 4684.2)	-1.24 (-1.44 to -1.05)
East Asia	3283374.7 (2783790.53 to 3921207.02)	3239.33 (1953.61 to 5085.77)	3805845.7 (3152823.25 to 4639794.68)	2412.99 (1697.93 to 3433.21)	-1.04 (-1.31 to -0.76)
Eastern Europe	743956.49 (689018.29 to 811249.52)	2063.84 (1664.76 to 2656.02)	562713.96 (513820.92 to 618254.29)	2329.85 (1781.5 to 3100.32)	0.39 (0.23 to 0.54)
Eastern Sub-Saharan Africa	2351494.22 (2253003.27 to 2464207.12)	3985.82 (3046.35 to 5265.03)	3145746.52 (2996586.13 to 3307121.1)	2808.2 (2232.53 to 3539.49)	-1.21 (-1.28 to -1.14)
High-income Asia Pacific	365111.75 (330589.31 to 410697.6)	1272.74 (1108.46 to 1507.51)	259914.07 (233539.32 to 294771.26)	1313.35 (1124.76 to 1579.38)	0.09 (-0.02 to 0.2)
High-income North America	321928.85 (252851.68 to 414653.04)	642.49 (457.81 to 911.67)	363860.71 (275816.38 to 474861.6)	679.15 (469.04 to 982.31)	0.17 (0.07 to 0.28)
North Africa and Middle East	3036488.89 (2874883.31 to 3244454.42)	3419.89 (2647.53 to 4406.09)	4279252.11 (3997982.73 to 4639215.69)	3265.97 (2726.52 to 3981.35)	-0.19 (-0.22 to -0.16)
Oceania	61115.19 (58256.35 to 64687.97)	4073.97 (3056.55 to 5459.99)	103760.94 (97954.97 to 110151.99)	3843.95 (2892.36 to 5110.39)	-0.33 (-0.54 to -0.12)
South Asia	27226399.43 (25859178.78 to 28955700.22)	8641.37 (6923.5 to 11042.53)	25365704.22 (23212695.71 to 28071778.59)	7861 (6123.81 to 10040.09)	-0.36 (-0.44 to -0.28)
Southeast Asia	4576165.48 (4286016.31 to 4942823.94)	4828.32 (3617.4 to 6448.55)	4655692.59 (4278864.35 to 5138607.94)	4106.73 (3260.07 to 5250.54)	-0.56 (-0.77 to -0.35)
Southern Latin America	97678.62 (86506.15 to 111589.58)	951.9 (753.86 to 1206.51)	125876.14 (110477.42 to 147087.1)	1015.36 (832.78 to 1249.32)	0.17 (-0.05 to 0.39)
Southern Sub-Saharan Africa	323475.34 (307508.4 to 343125.71)	2413.53 (1867.41 to 3222.83)	285974.85 (268249.8 to 307538.79)	2096.6 (1596.32 to 2771.82)	-0.51 (-0.79 to -0.23)
Tropical Latin America	323129.09 (292445.28 to 365516.27)	1325.53 (927.19 to 1857.95)	431137.2 (397153.01 to 475140.24)	1139.58 (951.7 to 1401.66)	-0.57 (-0.68 to -0.45)
Western Europe	760860.61 (629062.83 to 930294.09)	1351.89 (1011.58 to 1841.19)	741588.33 (604570.53 to 918853.68)	1358 (1000.31 to 1877.21)	-0.01 (-0.18 to 0.16)
Western Sub-Saharan Africa	2565692.18 (2483168.89 to 2666258.76)	4363.28 (3350.69 to 5720.53)	5204959.56 (4981654.7 to 5482227.22)	3595.06 (2958.03 to 4432.47)	-0.69 (-0.83 to -0.54)

**Table 2:** Prevalence number and ASR of nutritional deficiencies among children in 1990 and 2019, and AAPC of ASR from 1990 to 2019

Prevalence	1990		2019		1990–2019
	Cases NO.(95%UI)	ASR/100,000 (95% CI)	Cases NO.(95%UI)	ASR/100,000 (95% CI)	AAPC% (95%CI)
Global	427538173.72 (419116917.08 to 435738379.57)	24159.03 (23473.13 to 24885.85)	435071628.35 (425910405.22 to 444749386.4)	22399.28 (21697.2 to 23100.36)	-0.27 (-0.29 to -0.25)
Sex					
Boy	217961120.75 (212461725.02 to 223205808.92)	23942.69 (22995.96 to 24891.39)	221385750.05 (214965889.85 to 228027312.86)	22103.89 (21118.66 to 23133.46)	-0.28 (-0.3 to -0.25)
Girl	209577052.97 (204759820.79 to 214397446.43)	24383.25 (23517.10 to 25260.62)	213685878.29 (208289369.43 to 218556332.6)	22713.61 (21883.14 to 23513.60)	-0.24 (-0.26 to -0.23)
SDI					
High SDI	13042145.85 (12085614.93 to 14090404.74)	7670.83 (6737.3 to 8728.86)	8789314.15 (7912734.22 to 9795651.56)	5550.85 (4645.77 to 6633.56)	-1.1 (-1.16 to -1.05)
High-middle SDI	47055997.46 (45185204.41 to 49017657.37)	15589.71 (14558.59 to 16654.85)	27066579.88 (25753819.51 to 28469019.99)	11185.44 (10286.77 to 12156.93)	-1.15 (-1.17 to -1.12)
Middle SDI	121457409.45 (117818360.72 to 125071188.66)	20840.04 (19874.23 to 21876.56)	87610999.31 (84568163.1 to 90736517.23)	16070.83 (15202.45 to 16936.67)	-0.89 (-0.94 to -0.85)
Low-middle SDI	152715843.43 (148369382.95 to 156558693.48)	33169.77 (31848.03 to 34461.13)	145281665.75 (141116715.93 to 149724155.62)	28182.19 (26936.41 to 29523.89)	-0.57 (-0.59 to -0.54)
Low SDI	93040316.68 (91055637.67 to 95034794.93)	37338.54 (36055.12 to 38664.53)	166067518.41 (162306392.91 to 169819823.89)	34848.07 (33519.15 to 36205.93)	-0.24 (-0.24 to -0.23)
GBD regions					
Andean Latin America	3312670.77 (3003432.97 to 3677350.58)	21887.6 (18193.14 to 25849.17)	2731712.77 (2375935.18 to 3104033.69)	15083.03 (12055.98 to 18680.62)	-1.29 (-1.34 to -1.23)
Australasia	452010.93 (339838.22 to 593351.52)	10099.66 (6525.96 to 15181.6)	406253.6 (289655.41 to 559396.99)	7661.7 (4707.83 to 12248.94)	-0.95 (-0.98 to -0.92)
Caribbean	2475705.58 (2295155.38 to 2650613.84)	21513.17 (18975.85 to 24292.81)	2525074.79 (2318653.37 to 2730995.9)	21816.73 (18998.58 to 24894.72)	0.06 (0.03 to 0.08)
Central Asia	6169059.32 (5803801.02 to 6525179.61)	24080.92 (21649.95 to 26640.02)	5505676.85 (5027648.01 to 5995790.7)	20250.6 (17308.75 to 23364.37)	-0.61 (-0.65 to -0.57)
Central Europe	4206401.09 (3836976.36 to 4582803.74)	15230.06 (13083.18 to 17499.8)	1913944.3 (1711022.05 to 2139635.41)	11267.46 (9384.59 to 13584.93)	-1.03 (-1.05 to -1.01)
Central Latin America	9192531.4 (8737600.67 to 9705081.58)	14241.45 (13113.09 to 15499.98)	6798067.69 (6364501.33 to 7226248.32)	10648.93 (9663.8 to 11729.26)	-1 (-1.02 to -0.98)
Central Sub-Saharan Africa	9305417.1 (8745324.71 to 9896496.68)	35265.71 (31503.99 to 38994.46)	19046346.76 (17572990.17 to 20611885.64)	33158.26 (29238.85 to 37291.97)	-0.21 (-0.27 to -0.15)
East Asia	43540380.72 (40621807.58 to 46575743.93)	12913.96 (11428.86 to 14486.66)	13264058.79 (11894525.65 to 15066393.1)	5632.23 (4663.83 to 6775.98)	-2.84 (-2.92 to -2.76)
Eastern Europe	4509274.44 (3961114.03 to 5123504.97)	8927.23 (7156.05 to 11153.17)	2483223.82 (2039694.15 to 3036993.13)	6850.83 (5106.01 to 9385.69)	-0.91 (-0.99 to -0.83)
Eastern Sub-Saharan Africa	30253957.42 (29477970.54 to 31085275.03)	32464.23 (31092.14 to 33893.09)	51751685.27 (50066321.52 to 53494965.4)	29013.16 (27463.51 to 30604.96)	-0.39 (-0.42 to -0.36)
High-income Asia Pacific	3688925.79 (3,081,159 to 4389485.49)	11032.35 (8100.85 to 14970.07)	1644464.5 (1285276.69 to 2082617.9)	7334.47 (4948.03 to 10826.92)	-1.4 (-1.43 to -1.36)
High-income North America	2,736,783 (2267189.81 to 3221669.99)	4455.18 (3244.22 to 5986.88)	2691906.17 (2,043,973 to 3464132.74)	4157.25 (2605.55 to 6311.11)	-0.26 (-0.34 to -0.19)
North Africa and Middle East	31667374.35 (30215764.7 to 33258190.43)	21542.74 (19708.3 to 23486.42)	30702567.64 (28911681.61 to 32699538.38)	17634.29 (15983.34 to 19424.52)	-0.69 (-0.71 to -0.67)
Oceania	680578.81 (624417.44 to 741498.16)	25300.34 (21775.23 to 28882.38)	1234865.73 (1108438.77 to 1358192.71)	24721.99 (20513.38 to 29253.53)	-0.08 (-0.11 to -0.06)
South Asia	177,686,205 (172762062.31 to 182129724.18)	39965.29 (38404.75 to 41574.59)	175315418.13 (170141183.26 to 180859582.95)	34629.26 (33011.33 to 36310.4)	-0.5 (-0.52 to -0.47)
Southeast Asia	43043216.66 (40735023.76 to 45556462.97)	25055.83 (22846.62 to 27417.94)	26625176.44 (24842233.91 to 28508595.91)	16291.32 (14658.9 to 18151.54)	-1.49 (-1.53 to -1.45)
Southern Latin America	2254598.19 (1972286.5 to 2587027.56)	15178.24 (12036.75 to 18935.13)	1561766.45 (1245267.64 to 1911273.3)	10855.03 (7442.84 to 15077.54)	-1.15 (-1.23 to -1.07)
Southern Sub-Saharan Africa	4136325.36 (3748563.36 to 4565291.13)	20248.41 (16735.05 to 23829.14)	4094501.1 (3681025.99 to 4562848.23)	17396.17 (14641.99 to 20733.09)	-0.52 (-0.56 to -0.48)
Tropical Latin America	9640746.91 (8209698.15 to 11298816.22)	18136.87 (13616.33 to 23446.72)	6601914.66 (5410937.76 to 7955812.18)	13608.71 (9654.23 to 18315.45)	-0.98 (-1 to -0.97)
Western Europe	5228875.2 (4680301.63 to 5844334.19)	7596.76 (6318.05 to 9057.72)	3530913.66 (3057241.26 to 4044236.9)	5302.55 (4252.03 to 6580.18)	-1.23 (-1.3 to -1.17)
Western Sub-Saharan Africa	33357135.68 (32290817.76 to 34497517.85)	36670.37 (34596.88 to 38709.21)	74642089.22 (70967310.47 to 78347904.17)	37218.83 (34341.32 to 39968.12)	0.05 (0.04 to 0.07)

**Table 3:** DALY number and ASR of nutritional deficiencies among children in 1990 and 2019, and AAPC of ASR from 1990 to 2019

DALY	1990		2019		1990–2019
	Cases NO.(95%UI)	ASR/100,000 (95% CI)	Cases NO.(95%UI)	ASR/100,000 (95% CI)	AAPC% (95%CI)
Global	67004080.08 (52751020.67 to 86643263.5)	3729.04 (2847.3 to 4908.53)	26339578.19 (20235568.07 to 33595122.77)	1363.88 (1031.97 to 1776.16)	-3.41 (-3.63 to -3.19)
Sex					
Boy	33419025.1 (26637974.67 to 40916879.04)	3614.15 (2807.38 to 4503.39)	13025360.94 (9864886.46 to 16811589.29)	1306.97 (979.63 to 1716.97)	-3.46 (-3.68 to -3.24)
Girl	33585054.99 (24142068.49 to 46837276.24)	3850.55 (2715.66 to 5429.04)	13314217.25 (10110307.49 to 17084398.46)	1424.63 (1063.49 to 1873)	-3.3 (-3.47 to -3.12)
SDI					
High SDI	341980.75 (228033.75 to 487956.62)	199.81 (131.57 to 289.29)	197857.84 (127021.31 to 290459.55)	122.5 (76.06 to 184.44)	-1.68 (-1.76 to -1.59)
High-middle SDI	2708169.98 (2133675.99 to 3410532.86)	899.32 (690.97 to 1153.36)	889866.58 (609368.87 to 1255173.37)	366.94 (250.58 to 521.57)	-3.05 (-3.15 to -2.95)
Middle SDI	10466118.15 (8593891.46 to 12843715.96)	1789.5 (1452.42 to 2245.48)	3672925.78 (2653216.48 to 4948693.12)	674.83 (488.86 to 914.71)	-3.34 (-3.43 to -3.26)
Low-middle SDI	30738507.97 (22278511.41 to 41320066.1)	6470.76 (4546.44 to 8835.53)	8014898.63 (6,051,123 to 10526655.07)	1567.18 (1163.53 to 2060.43)	-4.76 (-5.27 to -4.25)
Low SDI	22718618.86 (18012183.07 to 29698464.9)	8440.29 (6539.33 to 11248.41)	13549000.3 (10635432.97 to 16958567.26)	2812 (2115.95 to 3657.25)	-3.73 (-3.9 to -3.57)
GBD regions					
Andean Latin America	511677.4 (423122.87 to 621655.31)	3309.88 (2628.55 to 4216.81)	120317.29 (86916.85 to 161469.57)	664.05 (449.27 to 940.4)	-5.46 (-5.56 to -5.37)
Australasia	8860.13 (5057.58 to 15407.74)	197.11 (95.42 to 389.69)	6995.77 (3819.4 to 12018.66)	130.77 (60.24 to 261.22)	-1.4 (-1.45 to -1.35)
Caribbean	412817.06 (317256.35 to 543963.11)	3510.89 (2605.76 to 4785.57)	162804.51 (120079.26 to 218291.64)	1417.43 (974.11 to 2029.73)	-3.08 (-3.24 to -2.91)
Central Asia	267502.23 (189977.96 to 369112.66)	1039.6 (722.49 to 1477.17)	184141.18 (121262.74 to 261707.83)	677.64 (422.75 to 1014.64)	-1.47 (-1.52 to -1.43)
Central Europe	130528.41 (85912.89 to 189186.58)	467.79 (295.82 to 699.94)	48429.49 (30899.46 to 71826.82)	281.55 (172.93 to 432.63)	-1.73 (-1.78 to -1.69)
Central Latin America	1147864.39 (1001992.5 to 1310773.37)	1762.32 (1503.8 to 2043.65)	323253.84 (251195.63 to 410457.11)	509.14 (385.13 to 663.03)	-4.19 (-4.37 to -4.01)
Central Sub-Saharan Africa	2441366.7 (1699192.53 to 3479392.65)	8156.01 (5499.16 to 11932.09)	1243314.72 (907797.06 to 1663642.13)	2133.92 (1475.85 to 2978.16)	-4.51 (-4.6 to -4.43)
East Asia	4106152.11 (3411183.28 to 4907729.31)	1204.44 (950.97 to 1490.85)	371575.37 (262424.15 to 512108.85)	158.33 (105.3 to 231.15)	-6.9 (-8.72 to -5.03)
Eastern Europe	122277.87 (83798.06 to 172575.93)	240.47 (155.29 to 365.12)	56215.84 (36374.57 to 84279.58)	153.2 (90.4 to 251.25)	-1.55 (-1.65 to -1.46)
Eastern Sub-Saharan Africa	10488649.6 (8273360.92 to 13458073.08)	10389.59 (8053.71 to 13526.02)	4887294.47 (3861116.92 to 6101895.69)	2700.64 (2064.35 to 3504.95)	-4.51 (-5.01 to -4.01)
High-income Asia Pacific	101581.9 (62240.92 to 153980.06)	295.91 (169.18 to 485.51)	36424.67 (21680.33 to 58544.85)	158.86 (81.84 to 282.03)	-2.12 (-2.18 to -2.06)
High-income North America	58239.77 (36593.66 to 87533.15)	94.91 (55.13 to 151.95)	58525.87 (34886.61 to 92182.79)	88.13 (44.76 to 156.32)	-0.28 (-0.38 to -0.17)
North Africa and Middle East	2346154.01 (1692013.85 to 3634202.9)	1568.58 (1102.02 to 2501.05)	1244436.21 (895835.72 to 1725576.38)	716 (500.53 to 1003.64)	-2.65 (-2.76 to -2.54)
Oceania	42881.7 (32434.11 to 55839.05)	1574.59 (1136.52 to 2144.04)	59387.74 (41574.98 to 80694.99)	1181.19 (779.16 to 1706.55)	-0.98 (-1.25 to -0.72)
South Asia	32935873.86 (23396975.49 to 44554668.52)	7201.79 (4939.54 to 9871.73)	9600280.24 (6996736.8 to 12771201.26)	1915.32 (1378.42 to 2585.07)	-4.45 (-4.51 to -4.38)
Southeast Asia	3748702.19 (2855091.79 to 5015088.52)	2188.48 (1565.36 to 3032.9)	1053103.84 (768035.42 to 1424535.44)	645.76 (460.81 to 881.21)	-4.14 (-4.22 to -4.05)
Southern Latin America	129707.04 (103855.83 to 165808.31)	874.69 (672.31 to 1165.11)	41516.83 (27137.93 to 63328.17)	287.74 (168.64 to 491.18)	-3.81 (-4.22 to -3.41)
Southern Sub-Saharan Africa	721075.85 (571850.37 to 904773.37)	3512.03 (2682.44 to 4557.02)	426811.77 (323456.21 to 545320.47)	1827.65 (1323.21 to 2472.44)	-2.17 (-2.69 to -1.66)
Tropical Latin America	1397103.69 (1187212.59 to 1642584.9)	2730.03 (2245.58 to 3345.42)	300058.31 (218826.79 to 412879.09)	623.55 (414.92 to 919.1)	-4.97 (-5.11 to -4.83)
Western Europe	122908.29 (79155.03 to 176072.06)	175.42 (108.33 to 260.66)	81039.61 (52000.87 to 117469.84)	118.45 (72.4 to 181.86)	-1.36 (-1.44 to -1.27)
Western Sub-Saharan Africa	5762155.88 (4540596.2 to 7223029.13)	5849.09 (4461.75 to 7575.25)	6033650.61 (4635254.4 to 7718440.27)	2961.72 (2177.06 to 3944.28)	-2.32 (-2.49 to -2.15)

### Regional and national burden

Regionally, in 2019, the highest ASIR was observed in South Asia (7861 (95% CI: 6123.81 to 10040.09) per 100,000 children), followed by Southeast Asia (4106.73 (3260.07 to 5250.54) per 100,000 children) and Oceania (3843.95 (2892.36 to 5110.39) per 100,000 children). The highest ASPR and age-standardized DALY rate of childhood malnutrition were observed in Western Sub-Saharan Africa (ASPR: (37218.83 (95% CI: 34341.32 to 39968.12) per 100,000 children); DALY rate: (2961.72 (2177.06 to 3944.28) per 100,000 children)), with High-income North America being the lowest estimates. Since 1990, the ASPR of childhood malnutrition has declined in most regions, with the greatest decline observed in East Asia, followed by Southeast Asia and high-income Asia Pacific. The largest decrease in ASIR was recorded in Central Sub-Saharan Africa, followed by Eastern Sub-Saharan Africa and Andean Latin America. However, the greatest increases were seen in Eastern Europe for ASIR. Regarding the age-standardized DALY rate, all regions have shown a downward trend from 1990 to 2019, with the greatest decrease being observed in East Asia.

**Fig. 3:**
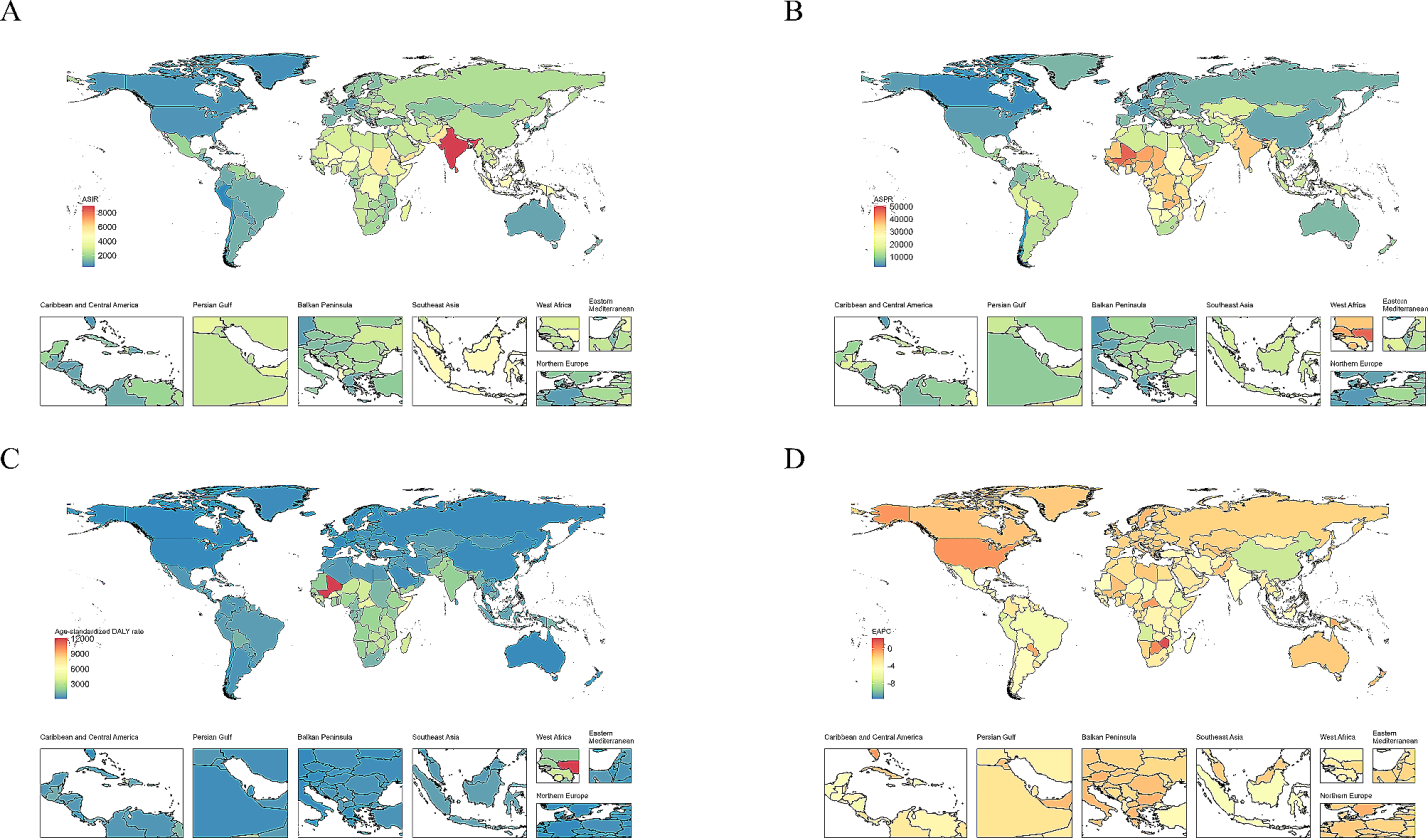
Incidence, prevalence, and DALY rates of children with nutritional deficiency in different countries and territories in 2019. (**A**) age-standardized incidence rate; (**B**) age-standardized prevalence rate; (**C**) age-standardized DALY rate; (**D**) estimated annual percent change of DALY rate

Nationally, India (133,648,266) had the highest number of affected children in 2019, which accounted for 30.72% of the total number of children affected globally. The vast majority of countries have shown decreasing trends in ASRs over the study period. Equatorial Guinea showed the highest decline in ASIR, whereas Ecuador had the greatest decrease in ASPR. Meanwhile, the Democratic People’s Republic of Korea experienced the greatest decrease in the age-standardized DALY rate. In contrast, Zimbabwe was the only country where the age-standardized DALY rate increased. Taiwan (Province of China) had the greatest increase in ASIR among all nations and territories, whereas Yemen had the greatest increase in ASPR. In 2019, the highest ASIR of childhood malnutrition was shown in India (8930.63 (95% CI: 6824.33 to 11518.57)/100,000), whereas Peru (428.94 (344.44 to 534.57)/100,000) had the lowest reported ASIR (Fig. 3 and Supplementary Table 3). Notably, the ASPR of Bhutan was up to 50398.46 (95% CI: 43741.28 to 57065.8) per 100,000 children, with Chile (2112.67 (1096.34 to 4085.94)/100,000) being the lowest (Supplementary Table 4). As for age-standardized DALY rate, Mali (12124.41 (8258.07 to 17515.91)/100,000) carried the heaviest burden, with Chile (47.08 (25.28 to 89.36)/100,000) having the lowest estimate (Supplementary Table 5). As can be seen, all ASRs varied by over 20 times from country to country, indicating that the burden of child malnutrition disparately varied across countries.

### Childhood malnutrition burden and SDI

**Fig. 4:**
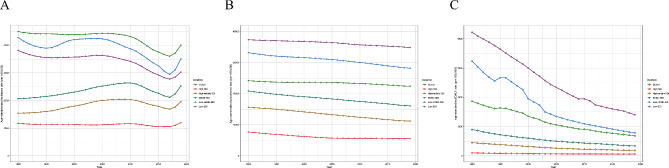
Incidence, prevalence, and DALY rates of children with nutritional deficiency in five SDI groups from1990 to 2019. (**A**) incidence; (**B**) prevalence; (**C**) DALY rate

**Fig. 5:**
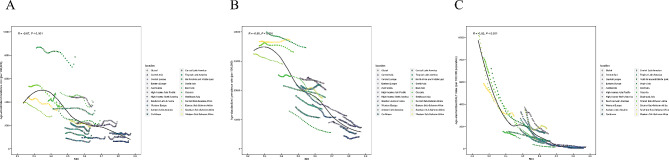
Incidence, prevalence, and DALY rates of children with nutritional deficiency across 21 GBD regions by SDI. (**A**) incidence; (**B**) prevalence; (**C**) DALY rate

Childhood malnutrition burden differed substantially based on SDI. Of the children with nutritional deficiency in 2019, 166.07 million (38.17%) lived in low-income countries and 8.79 million (2.02%) in high-income ones. As shown in Figs. 4 and 5, ASIR, ASPR, and age-standardized DALY rates of childhood malnutrition were more notable in lower SDI quintiles. In other word, ASIR (*R* = -0.67, *P* < 0.05), ASPR (*R* = -0.88, *P* < 0.05), and age-standardized DALY rate (*R* = -0.83, *P* < 0.05) were all negatively correlated with SDI. Since 1990, the ASPR and age-standardized DALY rates have decreased in all five SDI regions. Low-middle SDI countries showed the largest decrease in age-standardized DALY rate, but high SDI countries showed the lowest declines. Among the five SDI regions, the high-middle SDI region showed the most significant growth in ASIR.

### Different malnutrition burdens among children

The global burden of different malnutrition subtypes in 2019 is listed in Supplementary Tables 6, 7, 8, 9 and 10. Iron deficiency was the most prevalent ND among children with malnutrition of both sexes worldwide, accounting for about 90% of all ND. 391.49 million (95% UI: 382.83 to 400.50) children suffered from iron deficiency in 2019, corresponding to a prevalence of 20,146.35 (95% CI: 19,407.85 to 20,888.54) per 100,000 children. Vitamin A deficiency was the second most prevalent malnutrition, affecting 209.67 million (196.24 to 225.11) children. Notably, the prevalence of vitamin A deficiency in children was as high as 10779.02 (95%CI: 9727.6 to 12133.06) per 100,000 children. Iodine deficiency was the least prevalent malnutrition during the period. Iron deficiency was associated with the highest DALYs, followed by protein-energy malnutrition, Vitamin A deficiency, and iodine deficiency.

Except for protein-energy malnutrition, the low SDI areas recorded the highest number of cases and prevalence of the remaining three types of malnutrition among five SDI areas. The number of prevalent cases of iron deficiency, vitamin A deficiency, and iodine deficiency among children in low SDI areas in 2019 were 154,901,335 (150,630,641 to 159,216,170), 106,741,856 (100,467,847 to 113,414,826), and 5,889,501 ( 3,971,517 to 8,153,952), respectively. Prevalence rates of these three types of malnutrition in low SDI areas were 32,516.96 (30,993.53 to 34,036.61), 22,389.56 (20,252.91 to 24,796.64) and 1275.4 (861.44 to 1762.91) per 100,000 children, respectively. The prevalent cases of protein-energy malnutrition among children in low-middle SDI areas was as high as 25,102,290 (23,894,967 to 26,615,434) with a prevalence of 5009.55 (4734.76 to 5340.25) per 100,000 children.

Among 21 GBD regions, South Asia (34,174,598 (32,267,712 to 36,551,526)) recorded the highest prevalent cases of protein-energy malnutrition in children in 2019, followed by Western Sub-Saharan Africa (8,704,211 (8,472,115 to 8,980,617)) and Southeast Asia (6,724,680 (6,372,986 to 7,149,002)). Meanwhile, South Asia (7069.86 (6645.26 to 7577.03) per 100,000 children) showed the highest prevalence of protein-energy malnutrition among children. Notably, South Asia also experienced the highest prevalent cases of vitamin A deficiency (47,190,262 (36,397,675 to 60,576,338)) and iodine deficiency (4,881,808 (3,116,618 to 7,125,750)). The top 3 prevalence rates of vitamin A deficiency in children were Central Sub-Saharan Africa (31,206.7 (23,673.5 to 39,486.02) per 100,000 children), Eastern Sub-Saharan Africa (25,052.34 (21,931.14 to 28,528.86) per 100,000 children) and Western Sub-Saharan Africa (18,803.86 (16,846.05 to 21,093.73) per 100,000 children). The prevalence of iodine deficiency was also highest in Central Sub-Saharan Africa (5478.62 (3785.45 to 7239.22) per 100,000 children), followed by South Asia (887.18 (565.43 to 1299.87) per 100,000 children) and Eastern Sub-Saharan Africa (788.37 (505.73 to 1172.02) per 100,000 children).

## Discussion

To our knowledge, this study is the first comprehensive analysis of the burden of child malnutrition at the global, regional, and national levels over the past 30 years. About 435.1 million children worldwide suffered from malnutrition investigated by the GBD group in 2019 compared with 427.5 million in 1990. This marginal change is in sharp contrast with the trend in DALY, which substantially decreased from 67 million in 1990 to 26.3 million in 2019. The results of the joinpoint regression showed a global decline in the prevalence (AAPC = -0.27% (95% CI: -0.29% to -0.25%)) and DALY (-3.41% (-3.63% to -3.19%)) of childhood malnutrition, which may be attributed to the implementation and advancement of The Millennium Development Goals [30]. Nevertheless, the burden of malnutrition on children is still at a high level. A systematic evaluation in 2022 confirmed the high burden of malnutrition-related complications of children in low-income and middle-income countries, including tuberculosis, pneumonia, gastroenteritis, and anemia [31]. Child malnutrition poses a huge challenge to children’s health and social development.

According to SDI, the burden of child malnutrition varied considerably across regions and countries. There was a negative correlation between the burden of child malnutrition and SDI, indicating that the higher the socio-economic level, the lower the burden of child malnutrition. ASIR and ASPR for child malnutrition were significantly higher in the low SDI quintile compared to countries with high SDI. We found that prevalent cases were approximately 19 times higher in low SDI regions than in high SDI regions in 2019, and the ASPR in low SDI areas was 6 times higher than high SDI areas. At the same time, among five SDI areas, the lowest incidence reduction was observed in low SDI regions. The DALY rate in low SDI areas (2812 per 100,000 children) was 23 times higher than high SDI areas (122.5 per 100,000 children). The huge disparities in the burden of malnutrition between different countries were due to a multitude of reasons. In countries with high SDI, low prevalence and DALY were attributed to greater parental nutritional awareness, advanced diagnostic and treatment technologies, and easier access to health care. Unfortunately, the majority of children with malnutrition live in low-income and middle-income countries, where medical resources are very limited. The high prevalence of child malnutrition in poor areas may be attributed to poor conditions such as low levels of social development, lack of awareness of childcare, and barriers to education. A study in Bangladesh found that socio-economic inequalities in child malnutrition were stark. Stunting and underweight were more concentrated among children born to mothers from poorer households and with lower levels of education [32]. A study in Pakistan also found that better socio-economic status and well-being in the household reduces malnutrition and health risks in children [33], which is consistent with the findings of a study in Zimbabwe [34]. In the future, there is a need to strengthen interregional cooperation to gradually spread medical advances and health awareness in high-income countries to low- and middle-income countries to improve their healthcare capacity.

At the age level, we found that malnutrition was significantly more prevalent among children < 5 years of age. Preschool children are at risk for childhood malnutrition. Malnutrition, including stunting, severe wasting, vitamin A and zinc deficiencies, and suboptimal breastfeeding, has been the underlying cause of about one-third of under-five deaths [1]. In 2016, an estimated 155 million children younger than 5 years of age were stunted and 52 million were wasted, including 17 million who were severely wasted [35]. Statistically, more than half of all children under five years of age with stunting two-thirds of all children with wasting in Asia, and more than one-third of all children with stunting and one-quarter of all children with wasting in Africa [36].

It is found that Asia and Africa continue to have the highest burden of child malnutrition. However, in comparison, Asia has improved while Africa seems to be less pronounced. Some dietary guidance policies are needed in Africa to contribute to the reduction of the burden of child malnutrition. In addition, we found that the incidence, prevalence, and DALY rates of child malnutrition have declined at variable rates several countries, but have even increased in others. Nationally, the number of children suffering from malnutrition is alarmingly high in India and it has the highest ASIR (36,390.4 per 100,000 children). Ecuador showed the greatest decline in prevalence rate over the past 30 years, from 19,033.34 in 1990 to 6788.37 in 2019, with an EAPC of -4.03 (95% CI: − 4.2 to -3.85), followed by China and Chile. In terms of incidence, Equatorial Guinea experienced the greatest reduction, with an EAPC of -4.93% (-5.66% to -4.2%), followed by Mozambique and Angola. In contrast, countries and territories such as Taiwan (Province of China), Australia, and Montenegro showed an increase in incidence rates, whereas Yemen, Burkina Faso, and Zimbabwe showed a rise in prevalence. Of the 204 countries and territories, Zimbabwe (EAPC = 1.98 (1.08 to 2.89)) was the only country where the age-standardized DALY rate increased over the past three decades. Zimbabwe is part of sub-Saharan Africa and is located in southern Africa. Poverty and inadequate maternal and child care are recognized as the main drivers of malnutrition in Zimbabwe. Child malnutrition in Zimbabwe is largely influenced by where children live and the wealth of their parents [34].

Despite a decline in the burden of childhood malnutrition, iron deficiency and protein-energy malnutrition remain the leading causes of childhood malnutrition leading to DALYs. Among the five subtypes, iron deficiency was the most common nutritional disorder in childhood, which is consistent with previous findings [37]. In 2017, the Global Burden of Disease study reported that dietary iron deficiency was the fourth and twelfth leading cause of years lived with disability in women and men, respectively [38]. It is worth noting that protein-energy malnutrition is the most severe form of malnutrition among children. Children with protein-energy malnutrition typically lack the amino acids required for growth and development, which can inhibit cell and body growth, leading to slow development and immune dysfunction [39].

There is a long way to go to improve childhood nutrition. Targeted measures are urgently needed to reduce the burden of ND on children. Firstly, preschool children are a group that requires focused attention. Secondly, screening and prevention of ND among children need to be strengthened in less economically developed regions. International cooperation and experience sharing should be enhanced to improve food security and sanitation for children in poor countries. It is also extremely necessary to reinforce the construction of primary healthcare institutions and to constantly revise and perfect relevant laws on the prevention and treatment of infectious diseases. In addition, strategies and policies should be carefully formulated for different types of ND, which will assist in reducing the overall incidence of ND. Last but not least, it is essential to combine the efforts of society, the school and the family in order to strengthen nutritional education for children, as well as to implement practical and targeted nutritional and dietary interventions. For instance, professional dietitians should be assigned to schools, and nutrition courses should be offered to guide children to establish a scientific and rational dietary pattern. In particular, it is necessary to focus on strengthening nutritional literacy education for parents to reduce the risk of micronutrient deficiencies by improving children’s diets.

There are some limitations to this study. Firstly, the quality of the data used in this study relies on the quality control of the original GBD data collection process, and bias is still inevitable. Furthermore, as the data were aggregated from multiple sites worldwide, there may be a limitation of underdiagnosis and underreporting, which may result in an underestimation of our results, and it is recommended that the findings be further validated with the help of a large cohort study. Finally, our study was limited by changes in the quality of GBD data and missing data. Due to the inability of the GBD database to obtain data on other subtypes of malnutrition, this study was unable to analyze them.

## Conclusion

Although the prevalence of child malnutrition declined globally between 1990 and 2019, it varied by region. Asia and Africa still carried the heaviest burden. Child malnutrition remains a major public health challenge, especially in regions with low SDI. Therefore, primary healthcare services in developing countries should be improved and effective measures, such as strengthening preschool education, enhancing nutritional support, and early and aggressive treatment, should be developed.

### Electronic supplementary material

Below is the link to the electronic supplementary material.

Supplementary Material 1

## Data Availability

The original contributions presented in the study are included in the article/Supplementary Material, further inquiries can be directed to the corresponding author/s.

## Abbreviations

ND: Nutritional deficiencies
GBD: Global Burden of Disease
ASR: Age-standardized rate
ASPR: Age-standardized prevalence rate
ASIR: Age-standardized incidence rate
DALY: Disability-adjusted life year
CI: Confidence interval
UI: Uncertainty interval
EAPC: Estimated annual percentage change
AAPC: Average annual percentage change
SDI: Sociodemographic index
