# Supplementary material for: Epidemiological study of pediatric nutritional deficiencies: an analysis from the global burden of disease study 2019
Source: Nutr J. 2024 Apr 18;23:44. doi: 10.1186/s12937-024-00945-1 (PMC11027389; doi:10.1186/s12937-024-00945-1)
Supplement: Supplementary file 1 — Supplementary Material 1 [file 12937_2024_945_MOESM1_ESM.docx]

**Appendix (Supplementary Materials)**

Epidemiological study of pediatric nutritional deficiencies: an analysis from the Global Burden of Disease Study 2019

**Supplementary Material:** Overview of nutritional deficiencies estimation methods in Global Burden of Disease Study 2019

All data for this study were obtained from the Global Burden of Disease Study (GBD) 2019 database via the official website [https://vizhub.healthdata.org/gbd-results/.](https://vizhub.healthdata.org/gbd-results/) The GBD study incorporates data from many various data sources, including censuses, household surveys, vital statistics, disease registries, disease notifications, health service use, websites of governments and international organizations and other sources. In GBD 2019, nutritional deficiencies were defined based on the 10th revision International Classification of Diseases (ICD) codes, stratified based on protein-energy malnutrition (ICD-10 codes E40-E46.9, E64.0), iodine deficiencies (E00-E02), vitamin A deficiencies (E50-E50.9, E64.1), dietary iron deficiencies (D50-D50.9), and other nutritional deficiencies such as vitamin deficiency anaemias, thiamine, niacin, vitamin D, vitamin C, calcium, selenium, and folate deficiency (D51-D53.9, E51-E61.9, E63-E64, E64.2-E64.9).

**Vitamin A deficiency**

Case definition

The case definition of vitamin A deficiency is the prevalence of serum retinol < 0.7 µmol/L.

In GBD 2019, the assessment of vitamin A deficiency burden involves the quantification of total vitamin A deficiency as well as blindness and vision loss due to vitamin A deficiency, which are associated with corneal ulcerations and corneal scars.

**Iodine Deficiency**

Case definition

Our assessment of the non-fatal burden of iodine deficiency includes estimates of only the subset of iodine deficiency associated with visible goiter (grade 2) and its associated sequelae, including thyroid dysfunction, heart failure, and intellectual disability (historically referred to as “cretinism”). It does not include estimates of sub-clinical iodine deficiency or non-visible goiter (grade 1) induced by iodine deficiency.

**Dietary Iron Deficiency**

Case definition

Dietary iron deficiency in the GBD cause analysis is defined as inadequate iron to meet the body’s needs due to inadequate dietary intake of iron, but not due to other causes of absolute or functional iron deficiency.

**Protein-energy malnutrition**

Case Definition

Protein-energy malnutrition (PEM) includes moderate and severe acute malnutrition, commonly referred to as “wasting”, and was defined in terms of weight-for-height Z-scores (WHZ) on the WHO 2006 growth standard for children. GBD collaborators quantified non-fatal PEM burden in four mutually exclusive and collectively exhaustive categories, reflecting distinct gradations of disability that can occur: moderate wasting without oedema (WHZ < -2SD to < -3 SD), moderate wasting with oedema (WHZ < -2SD to < -3 SD), severe wasting without oedema (WHZ < -3SD), and severe wasting with oedema (WHZ < -3SD). The aggregate of categories that include “oedema” can be considered equivalent to the disease state commonly referred to as “kwashiorkor” and severe wasting can likewise be considered equivalent to “marasmus.” For PEM, ICD 10 codes are E40-E46.9, E64.0, and ICD 9 codes are 260-263.9.

**Other nutritional deficiencies**

Other nutritional deficiencies encompass a wide variety of causes of morbidity, ranging from vitamin deficiencies to other nutritional anaemias. In GBD 2019, as done previously, GBD collaborators treat these causes as a single category, given their relatively limited burden, diversity in underlying causes and risk factors, and data availability. Instead of modelling them in a traditional modelling format, GBD collaborators calculate the YLDs associated with other nutritional deficiencies using a YLD/YLL ratio. The first input for this non-fatal portion of other nutritional deficiencies burden is the YLL estimates from the GBD 2019 causes of death (CoD) analysis. The causes and their associated ICD-10 codes that constitute other nutritional deficiencies for CoD are listed below. Additionally, CoD includes specific models for protein-energy malnutrition, another nutritional cause of morbidity and mortality; as proteinenergy malnutrition has a specific non-fatal model that results in YLDs, we can calculate the YLD/YLL ratio for protein-energy malnutrition. GBD collaborators multiply the YLL estimates for other nutritional deficiencies from CoD by the YLD/YLL ratio for PEM, providing us with an estimate of the YLDs associated with other nutritional deficiencies. There were no changes in modeling strategy for other nutritional deficiencies from GBD 2017.

**Supplementary Table1** Global incidence, Prevalence, and DALYs of children with nutritional deficiency in different age groups in 1990 and 2019 and the AAPC

|  | 1990 |  | 2019 |  | 1990-2019 |
| --- | --- | --- | --- | --- | --- |
|  | Cases  NO.(95%UI) | ASR/100,000 (95% CI) | Cases  NO.(95%UI) | ASR/100,000 (95% CI) | AAPC(% )  (95%CI) |
| Incidence |  |  |  |  |  |
| Both |  |  |  |  |  |
| <5 years | 69026306.74 (52608525.11 to 91969056.91) | 10919.88 (8322.6 to 14549.39) | 61336085.27 (48921131.37 to 77165328.8) | 9253.49 (7380.5 to 11641.58) | -0.58  (-0.66 to -0.5) |
| 5–9 years | 5964452.12 (4482564.24 to 7886795.43) | 1019.28 (766.04 to 1347.8) | 7318804.66 (5174825.88 to 10060282.56) | 1117.88 (790.41 to 1536.62) | 0.28  (0.04 to 0.52) |
| 10–14 years | 5893157.26 (4638699.91 to 7493803.41) | 1098.06 (864.32 to 1396.3) | 8166559.93 (6113007.79 to 10707540.97) | 1271.68 (951.91 to 1667.36) | 0.48  (0.41 to 0.56) |
| Girl |  |  |  |  |  |
| <5 years | 33366191.5 (24950334.86 to 45410361.91) | 10878.19 (8134.42 to 14804.88) | 29063589.29 (22996053.71 to 36514510.72) | 9069.79 (7176.31 to 11394.98) | -0.65  (-0.74 to -0.55) |
| 5–9 years | 2595935.59 (1966183.28 to 3402583.15) | 912.03 (690.78 to 1195.43) | 3082534.4 (2181771.77 to 4212471.68) | 973.16 (688.79 to 1329.89) | 0.22  (0.06 to 0.39) |
| 10–14 years | 2659703.04 (2109673.75 to 3347298.22) | 1015.47 (805.47 to 1277.99) | 3571927.4 (2712398.64 to 4670376.51) | 1149.07 (872.57 to 1502.44) | 0.43  (0.26 to 0.6) |
| Boy |  |  |  |  |  |
| <5 years | 35660115.24 (27623910.68 to 46606242.44) | 10959.17 (8489.46 to 14323.17) | 32272495.98 (25926698.79 to 40733994.47) | 9425.41 (7572.08 to 11896.65) | -0.51  (-0.62 to -0.4) |
| 5–9 years | 3368516.53 (2521370.46 to 4455177.1) | 1120.86 (838.98 to 1482.45) | 4236270.26 (2962584.21 to 5860188.4) | 1253.52 (876.64 to 1734.04) | 0.34  (0.2 to 0.47) |
| 10–14 years | 3233454.22 (2525613.04 to 4155558.78) | 1176.78 (919.17 to 1512.37) | 4594632.53 (3362571.89 to 6131094.8) | 1386.71 (1014.86 to 1850.43) | 0.52  (0.42 to 0.61) |
| Prevalence |  |  |  |  |  |
| Both |  |  |  |  |  |
| <5 years | 218904284.45 (215741221.43 to 222062668.37) | 34630.39 (34130 to 35130.04) | 208456140.32 (205130694.01 to 211832193.11) | 31448.81 (30947.12 to 31958.14) | -0.33  (-0.36 to -0.3) |
| 5–9 years | 129422397.51 (124861393.45 to 134129933.6) | 22117.35 (21337.91 to 22921.83) | 138713401.77 (133424351.89 to 143978257.1) | 21187.2 (20379.35 to 21991.36) | -0.16  (-0.2 to -0.12) |
| 10–14 years | 79211491.76 (74964284.91 to 84016063.68) | 14759.28 (13967.91 to 15654.5) | 87902086.25 (82694292.32 to 93060755.48) | 13687.93 (12876.99 to 14491.23) | -0.26  (-0.31 to -0.21) |
| Girl |  |  |  |  |  |
| <5 years | 104368719.1 (102369020.54 to 106350860.88) | 34026.74 (33374.79 to 34672.97) | 99192976.67 (97349970.7 to 101019046.3) | 30954.86 (30379.72 to 31524.72) | -0.33 (-0.35 to -0.3) |
| 5–9 years | 62418657.92 (59708188.01 to 65112875.54) | 21929.49 (20977.22 to 22876.04) | 66250828.29 (63269010.07 to 69123518.61) | 20915.51 (19974.15 to 21822.43) | -0.17  (-0.2 to -0.13) |
| 10–14 years | 42789675.96 (40141133.35 to 45563929.16) | 16337 (15325.8 to 17396.21) | 48242073.33 (45150275.74 to 51165746.1) | 15519.28 (14524.66 to 16459.81) | -0.18  (-0.22 to -0.14) |
| Boy |  |  |  |  |  |
| <5 years | 114535565.36 (112193384.81 to 116892863.91) | 35199.41 (34479.61 to 35923.87) | 109263163.66 (106659720.93 to 111840423.66) | 31911.09 (31150.73 to 32663.79) | -0.34  (-0.37 to -0.31) |
| 5–9 years | 67003739.59 (63553666.88 to 70428432.3) | 22295.27 (21147.27 to 23434.83) | 72462573.49 (68601466.97 to 76576693.94) | 21441.85 (20299.34 to 22659.23) | -0.15  (-0.21 to -0.09) |
| 10–14 years | 36421815.8 (33719942.44 to 39151393.95) | 13255.35 (12272.03 to 14248.75) | 39660012.92 (36126742.83 to 43422884.16) | 11969.79 (10903.42 to 13105.46) | -0.35  (-0.39 to -0.32) |
| DALY |  |  |  |  |  |
| Both |  |  |  |  |  |
| <5 years | 54640634.34 (42023019.77 to 71747436.39) | 8644.08 (6647.99 to 11350.36) | 16004379.25 (12805277.82 to 19785537.55) | 2414.51 (1931.87 to 2984.95) | -4.2  (-4.42 to -3.98) |
| 5–9 years | 8499421.05 (6374913.22 to 11180344.02) | 1452.49 (1089.43 to 1910.64) | 6569570.67 (4542055.9 to 9211068.38) | 1003.44 (693.76 to 1406.91) | -1.28  (-1.34 to -1.22) |
| 10–14 years | 3864024.69 (2778773.12 to 5258583.71) | 719.97 (517.76 to 979.82) | 3765628.26 (2551977.86 to 5350961.72) | 586.38 (397.39 to 833.24) | -0.71  (-0.78 to -0.65) |
| Girl |  |  |  |  |  |
| <5 years | 27235099.7 (19197444.97 to 38754923.26) | 8879.31 (6258.83 to 12635.05) | 8059971.12 (6292794.61 to 10120645.85) | 2515.25 (1963.77 to 3158.32) | -4.14  (-4.35 to -3.93) |
| 5–9 years | 4236106.62 (2996494.63 to 5726891.29) | 1488.27 (1052.76 to 2012.02) | 3164261.8 (2202137.51 to 4418904.22) | 998.96 (695.22 to 1395.06) | -1.36  (-1.46 to -1.26) |
| 10–14 years | 2113848.67 (1492065.66 to 2886022.65) | 807.06 (569.67 to 1101.88) | 2089984.33 (1431410.55 to 2987770.96) | 672.34 (460.48 to 961.15) | -0.63  (-0.73 to -0.54) |
| Boy |  |  |  |  |  |
| <5 years | 27405534.64 (21542674.78 to 33548588.3) | 8422.35 (6620.56 to 10310.25) | 7944408.13 (6291944.74 to 9937476.61) | 2320.22 (1837.61 to 2902.31) | -4.36  (-4.67 to -4.04) |
| 5–9 years | 4263314.43 (3175087.47 to 5669440.74) | 1418.6 (1056.5 to 1886.49) | 3405308.88 (2322946.22 to 4834455.1) | 1007.64 (687.37 to 1430.53) | -1.18  (-1.23 to -1.14) |
| 10–14 years | 1750176.03 (1254642.8 to 2393160.86) | 636.96 (456.61 to 870.97) | 1675643.93 (1134713.55 to 2358481.41) | 505.73 (342.47 to 711.81) | -0.81  (-0.86 to -0.75) |

**Supplementary Table2** Global trends for age-standardized rates (per 100,000 population) of nutritional deficiency among children younger than 15 years old from 1990 to 2019

| ASIR |  | ASPR |  | Age-standardized DALY rate |  |
| --- | --- | --- | --- | --- | --- |
| Year | APC(%)_95%CI | Year | APC(%)_95%CI | Year | APC(%)_95%CI |
| 1990-1999 | -0.24  (-0.34 to -0.14) | 1990-1993 | -0.48  (-0.59 to -0.37) | 1990-1993 | -3.82  (-5.1 to -2.52) |
| 1999-2006 | 0.2  (0.03 to 0.37) | 1993-1997 | -0.2  (-0.29 to -0.1) | 1993-1997 | -1.04  (-2.26 to 0.2) |
| 2006-2010 | -0.58  (-1.07 to -0.08) | 1997-2006 | -0.04  (-0.06 to -0.02) | 1997-2005 | -5.08  (-5.37 to -4.78) |
| 2010-2017 | -2.77  (-2.93 to -2.62) | 2006-2012 | -0.32  (-0.37 to -0.28) | 2005-2019 | -3.04  (-3.15 to -2.92) |
| 2017-2019 | 5.66  (4.62 to 6.72) | 2012-2019 | -0.46  (-0.49 to -0.43) |  |  |

**Supplementary Table3** Incidence of nutritional deficiency among children in 1990 and 2019 and the EAPC from 1990 to 2019 in different countries and territories

| Location | 1990 |  | 2019 |  | 1990-2019 |
| --- | --- | --- | --- | --- | --- |
|  | Incidence cases NO.(95%UI) | ASIR/100,000 (95% CI) | Incidence cases NO.(95%UI) | ASIR/100,000 (95% CI) | EAPC (95%CI) |
| Afghanistan | 86337.59 (80658.99 to 92074.79) | 3352.23 (2517.51 to 4422.49) | 387240 (358842.09 to 416202.4) | 3052 (2504.4 to 3773.08) | -0.8 (-0.92 to -0.68) |
| Albania | 13260.78 (11266.1 to 15741.28) | 4617.72 (2995.39 to 6526.58) | 7641.68 (6278.72 to 9378.26) | 4676.68 (2853.13 to 7212.93) | -0.12 (-0.44 to 0.21) |
| Algeria | 219268.98 (203810.18 to 237587.37) | 3176.04 (2526.6 to 4023.74) | 303346.92 (280853.82 to 329742.41) | 3043.37 (2598.34 to 3609.87) | -0.38 (-0.61 to -0.15) |
| American Samoa | 328.25 (307.19 to 352) | 2175.37 (1819.2 to 2641.39) | 261.02 (242.95 to 284.1) | 2091.17 (1815 to 2447.31) | -0.46 (-0.57 to -0.35) |
| Andorra | 88.01 (70.67 to 110.77) | 1249.51 (912.32 to 1738.34) | 102.32 (78.22 to 132.33) | 1308 (932.94 to 1847.51) | -0.19 (-0.32 to -0.05) |
| Angola | 170639.29 (156032.75 to 185506.22) | 4848.07 (3664.14 to 6624.8) | 246846.37 (221648.67 to 273445.71) | 2487.06 (1988.81 to 3087.19) | -2.77 (-3.08 to -2.47) |
| Antigua and Barbuda | 184.51 (166.99 to 204.52) | 1627.17 (1272.98 to 2075.1) | 157.3 (141.12 to 176.44) | 1500.83 (1198.24 to 1907.57) | -0.47 (-0.62 to -0.31) |
| Argentina | 71046.76 (62216.81 to 82319.88) | 1110.42 (854.36 to 1437.68) | 103389.42 (89780.45 to 121449.89) | 1175.78 (956.43 to 1452.61) | -0.23 (-0.4 to -0.06) |
| Armenia | 9740.75 (8881.54 to 10682.76) | 1532.65 (1132.71 to 2107.5) | 5416.86 (4866.33 to 6115.79) | 1683.38 (1166.76 to 2447.99) | 0.6 (0.41 to 0.79) |
| Australia | 34370.73 (31279.74 to 38012.21) | 997.01 (869.57 to 1154.12) | 40039.15 (36541.57 to 44502.59) | 968.55 (843.23 to 1139.51) | 0.67 (0.34 to 1) |
| Austria | 13995.82 (11223.3 to 17801.08) | 1311.53 (947.52 to 1844.06) | 14712.51 (11491.33 to 18862.27) | 1411.93 (1017.55 to 1993.61) | 0.3 (0.23 to 0.37) |
| Azerbaijan | 23142.89 (21302.4 to 25356.11) | 2535.23 (1733.07 to 3635.83) | 26368.98 (24076.97 to 29137.49) | 1875.57 (1455.77 to 2426.02) | -1.11 (-1.26 to -0.95) |
| Bahamas | 931.03 (772.97 to 1142.22) | 3215.84 (2184.79 to 4605.8) | 1473.1 (1179.44 to 1878.16) | 3317.9 (2365.71 to 4582.91) | -0.85 (-1.12 to -0.57) |
| Bahrain | 3242.18 (3000.78 to 3494.1) | 2460.51 (2045.52 to 3010.41) | 3583.75 (3199.31 to 4069.77) | 2471.84 (1866.95 to 3329.59) | -0.32 (-0.44 to -0.19) |
| Bangladesh | 2719359.78 (2620730.14 to 2819253.05) | 7085.73 (6035.83 to 8225.24) | 1384747.42 (1307359.44 to 1481225.12) | 4311.89 (3749.34 to 5053.42) | -2.02 (-2.22 to -1.83) |
| Barbados | 683.69 (622.5 to 758.14) | 1567.24 (1287.75 to 1939.87) | 550.32 (483.81 to 628.66) | 1696.14 (1347.95 to 2142.33) | 0.03 (-0.17 to 0.23) |
| Belarus | 19804.88 (17469.44 to 22854.14) | 1835.24 (1279.61 to 2558.7) | 15468.96 (13821.19 to 17587.5) | 1760.59 (1259.6 to 2446.77) | -0.05 (-0.25 to 0.15) |
| Belgium | 17059.29 (14411.03 to 20734.35) | 1227.72 (922.03 to 1660.43) | 17833.57 (14787.55 to 21761.95) | 1197.91 (886.29 to 1634.06) | -0.34 (-0.51 to -0.17) |
| Belize | 545.05 (497.03 to 603.48) | 1675.97 (1166.64 to 2309.74) | 791.5 (712.4 to 905.26) | 1190.29 (929.53 to 1536.04) | -1.56 (-1.76 to -1.35) |
| Benin | 80050.01 (76290.3 to 84128.75) | 4315.96 (3429.83 to 5594.66) | 120614.85 (113560.21 to 127608.23) | 2783.84 (2311.04 to 3389.57) | -1.73 (-1.86 to -1.59) |
| Bermuda | 123.22 (110.52 to 139.22) | 1579.1 (1184.81 to 2130.04) | 85.1 (73.41 to 100.05) | 1998.13 (1353.87 to 2910.98) | 0.29 (0 to 0.58) |
| Bhutan | 6394.57 (5986.55 to 6872.08) | 4545.91 (3372.42 to 6213.46) | 6032.83 (5018.14 to 7347.6) | 5271.39 (3687.82 to 7420.8) | -0.2 (-0.44 to 0.04) |
| Bolivia (Plurinational State of) | 28981.73 (27421.08 to 30681.65) | 1304.46 (1075.57 to 1622.94) | 22477.86 (20327.32 to 25048.41) | 946.57 (719.17 to 1243.2) | -1.39 (-1.54 to -1.24) |
| Bosnia and Herzegovina | 10474.68 (8939.66 to 12167.83) | 1890.18 (1286.39 to 2785.59) | 4532.33 (3750.22 to 5457.04) | 1910.13 (1260.57 to 2842.61) | -0.12 (-0.21 to -0.03) |
| Botswana | 8686.52 (8182.59 to 9238.33) | 2155.58 (1775.51 to 2636.88) | 10111.08 (9494.7 to 10826.8) | 2147.95 (1746.96 to 2699.6) | 0.36 (0.07 to 0.65) |
| Brazil | 316932.2 (286809.5 to 358187.2) | 1341.31 (938.44 to 1883.1) | 423687.97 (390428.49 to 466468.72) | 1153.06 (966.25 to 1416.29) | -1.52 (-1.78 to -1.26) |
| Brunei Darussalam | 1400.78 (1288.67 to 1517.35) | 1557.45 (1367.45 to 1790.31) | 1312.24 (1201.06 to 1438.57) | 1570.27 (1379.74 to 1836.69) | -0.17 (-0.24 to -0.1) |
| Bulgaria | 17606.86 (15603.23 to 20234.97) | 1834.06 (1313.4 to 2592.7) | 11401.05 (10061.94 to 13161.22) | 2024.63 (1445.74 to 2872.39) | 0.47 (0.34 to 0.61) |
| Burkina Faso | 196428.48 (188986.22 to 204385.79) | 5020.9 (4247.97 to 6100.01) | 360637.79 (344933.51 to 376047.36) | 4239.18 (3620.33 to 5065.45) | -0.65 (-1.01 to -0.29) |
| Burundi | 86108.02 (82733.84 to 89450.75) | 4364.61 (3497.89 to 5582.72) | 86896.33 (81550.21 to 92185.8) | 2958.58 (2186.8 to 3931.13) | -1.55 (-1.63 to -1.47) |
| Cabo Verde | 1597.29 (1416.06 to 1794.02) | 1673.29 (1281.9 to 2155.92) | 1738.75 (1586.34 to 1903.82) | 1676.53 (1323.82 to 2144.45) | -0.91 (-1.24 to -0.58) |
| Cambodia | 125108.13 (117972.17 to 132871.53) | 5463.62 (3748.25 to 7782.84) | 110185.4 (102921.62 to 117544.52) | 3316.99 (2736.33 to 4150.85) | -2.05 (-2.33 to -1.78) |
| Cameroon | 81729.91 (77218.25 to 86350.51) | 2465.8 (1924.23 to 3187.29) | 169224.83 (157969.37 to 181878.59) | 2139.37 (1723.86 to 2674.88) | -1.16 (-1.35 to -0.97) |
| Canada | 29082.75 (24273.41 to 35639.84) | 642.25 (481.02 to 881.24) | 30872.18 (25057.81 to 38727.86) | 639.17 (468.53 to 879.72) | -0.29 (-0.44 to -0.14) |
| Central African Republic | 31785.97 (30170.6 to 33478.55) | 3827.46 (3031.38 to 4887.29) | 45554.98 (41955.34 to 49340.11) | 3072.78 (2395.22 to 3974.71) | -0.65 (-0.71 to -0.59) |
| Chad | 148115.6 (143425.84 to 153137.79) | 5889.87 (4875.62 to 7323.23) | 258011.4 (246047.88 to 270112.09) | 3938.85 (3274.29 to 4809.58) | -1.38 (-1.57 to -1.19) |
| Chile | 20698.61 (18486.08 to 23307.25) | 560.32 (469.07 to 669.99) | 16774.4 (14469.86 to 19526.21) | 544.05 (437.88 to 682.77) | -1.05 (-1.43 to -0.68) |
| China | 3129753.46 (2642895.13 to 3747784.05) | 3209.86 (1922.04 to 5044.04) | 3666202.39 (3030187.54 to 4484537.73) | 2378.55 (1668.42 to 3396.8) | -0.89 (-1.13 to -0.64) |
| Colombia | 84331.27 (77679.38 to 92245.62) | 1083.67 (883.41 to 1317.19) | 78309.74 (68113.93 to 91682.72) | 1095.05 (830.93 to 1443.53) | -0.36 (-0.51 to -0.21) |
| Comoros | 5896.84 (5581.19 to 6238.8) | 3576.69 (3031.63 to 4232.59) | 4314.36 (4076.15 to 4574.1) | 2569.94 (2216.41 to 3032.09) | -1.24 (-1.33 to -1.15) |
| Congo | 25116.48 (22169.99 to 27975.18) | 3250.63 (2514.04 to 4134.54) | 34919.1 (29771.13 to 40371.14) | 2333.14 (1847.21 to 2934.31) | -1.22 (-1.4 to -1.04) |
| Cook Islands | 103.56 (95.76 to 112.55) | 2106.82 (1746.16 to 2599.46) | 66.86 (61.37 to 74.02) | 2469.82 (1936.12 to 3243.1) | -0.14 (-0.32 to 0.05) |
| Costa Rica | 10532.61 (9290.14 to 12201.27) | 1047.15 (868.76 to 1276.76) | 10811.5 (8885.24 to 13535.45) | 1307.69 (986.1 to 1750.76) | -0.07 (-0.33 to 0.19) |
| Croatia | 9138.51 (8102.96 to 10394) | 1420.4 (1106.22 to 1845.58) | 5791.64 (5048.12 to 6687.22) | 1617.81 (1173.15 to 2274.06) | 0.41 (0.27 to 0.54) |
| Cuba | 18764.35 (16375.33 to 22211.33) | 1146.67 (869.5 to 1521.08) | 13387.43 (10880.37 to 16810.66) | 1486.65 (990.11 to 2211.62) | 0.54 (0.14 to 0.93) |
| Cyprus | 1879.51 (1592.03 to 2251.28) | 1264.54 (940.21 to 1722.82) | 2118.53 (1782.66 to 2544.79) | 1265.26 (951.84 to 1701.54) | -0.11 (-0.21 to -0.01) |
| Czechia | 17957.12 (15794.95 to 20581.46) | 1537.8 (1081.64 to 2205.12) | 15157.01 (13468.12 to 17376.73) | 1567.83 (1113.46 to 2238.07) | 0.06 (-0.09 to 0.21) |
| C么te d'Ivoire | 129604.8 (122031.37 to 137284.81) | NA | 195864.86 (184204.51 to 208431.85) | NA | NA |
| Democratic People's Republic of Korea | 123914.59 (113032.9 to 136764.8) | 5600.39 (3724.75 to 8232.56) | 121531.89 (107962.29 to 138096.73) | 4469.32 (3261.06 to 6017.99) | -2.43 (-2.89 to -1.98) |
| Democratic Republic of the Congo | 609082.33 (574463.33 to 646014.81) | 5762.35 (4446.51 to 7415.8) | 1089621.66 (944745.84 to 1240349.01) | 4317.1 (3304.43 to 5536.68) | -1.11 (-1.48 to -0.74) |
| Denmark | 9169.15 (7442.75 to 11347.73) | 1320.34 (966.66 to 1821.23) | 9292.7 (7607.83 to 11498.32) | 1251.44 (916.08 to 1738.37) | -0.35 (-0.47 to -0.23) |
| Djibouti | 11299.59 (10708.84 to 11916.7) | 5949.35 (4962.27 to 7267.02) | 16349.69 (15427.07 to 17347.51) | 4566.01 (3969.67 to 5338.67) | -1 (-1.17 to -0.83) |
| Dominica | 186.91 (169.13 to 207.85) | 1662.47 (1233.77 to 2244.51) | 105.99 (93.35 to 122.33) | 1642.25 (1210.05 to 2218.09) | -0.33 (-0.46 to -0.21) |
| Dominican Republic | 18730.32 (17327.84 to 20309.1) | 1685.74 (1204.14 to 2284.95) | 18978.91 (17440.27 to 21008.53) | 965.89 (760.4 to 1229.38) | -2.1 (-2.28 to -1.91) |
| Ecuador | 31911.48 (29844.5 to 33761.12) | 1056.79 (888.7 to 1272.98) | 38474.06 (35703.88 to 41901.85) | 901.92 (790.67 to 1047.26) | -1.02 (-1.27 to -0.77) |
| Egypt | 395018.51 (364853.12 to 429171.38) | 2863.51 (2197.32 to 3753.34) | 682476.68 (625449.81 to 755785.88) | 2625.17 (2208.13 to 3140.95) | -0.92 (-1.08 to -0.76) |
| El Salvador | 14674.03 (13455.05 to 16066.38) | 1195.72 (917.95 to 1547.77) | 11623.31 (10611.82 to 12854.11) | 941.98 (768.35 to 1166.08) | -1.04 (-1.31 to -0.77) |
| Equatorial Guinea | 12768.04 (11329.27 to 14101.56) | 6220.47 (5299.73 to 7205.01) | 10071.93 (9222.87 to 11020.94) | 2056.46 (1812.18 to 2330.65) | -4.93 (-5.66 to -4.2) |
| Eritrea | 58996.61 (56417.62 to 61772.24) | 5987.2 (4707.62 to 7839.51) | 64083.45 (60540.52 to 67843.4) | 3524.86 (2925.3 to 4294.67) | -1.96 (-2.12 to -1.79) |
| Estonia | 4477.75 (4093.43 to 4953.06) | 2161.23 (1643.85 to 2856.17) | 2603.36 (2362.96 to 2887.65) | 2016.77 (1496.96 to 2803.58) | 0.02 (-0.13 to 0.16) |
| Eswatini | 2646.84 (2423.74 to 2927.91) | 1687.33 (1122.52 to 2462.28) | 1727.2 (1509.51 to 1989.51) | 1438.25 (907.67 to 2101.56) | -0.49 (-0.66 to -0.32) |
| Ethiopia | 800141.59 (746342.45 to 864294.39) | 4749.53 (3640.53 to 6328.82) | 1149612.54 (1083796.32 to 1225445.89) | 3533.75 (2824.59 to 4491.2) | -1 (-1.29 to -0.71) |
| Fiji | 4952.87 (4655.63 to 5294.59) | 2324.97 (2024.69 to 2748.14) | 4741.12 (4411.43 to 5114.88) | 2261.72 (1947.21 to 2644.54) | -0.42 (-0.56 to -0.27) |
| Finland | 10694.97 (8666.23 to 13295.77) | 1388.59 (1027.71 to 1898.04) | 10056.46 (7829.64 to 13062.62) | 1425.25 (1043.31 to 1979.54) | -0.2 (-0.31 to -0.08) |
| France | 104630.58 (90491.36 to 123283.12) | 1154.86 (887.41 to 1537.88) | 109602.55 (91358.85 to 133092.29) | 1209.95 (903.56 to 1626.93) | -0.12 (-0.23 to 0) |
| Gabon | 6370.79 (5661.11 to 7117.82) | 2317.99 (1752.43 to 3030.19) | 6285.01 (5769.7 to 6856.55) | 1458.95 (1214.98 to 1783.37) | -2.18 (-2.35 to -2) |
| Gambia | 16877.57 (16021.22 to 17794.46) | 4572.17 (3691.13 to 5812.65) | 22512.45 (21061.23 to 24179.89) | 3303.16 (2729.42 to 4061.09) | -1.11 (-1.38 to -0.84) |
| Georgia | 10382 (9319.34 to 11693.82) | 1325.01 (959 to 1864.06) | 5752.59 (5164.33 to 6535.8) | 1327.65 (960.54 to 1843.69) | -0.09 (-0.42 to 0.24) |
| Germany | 87465.81 (66627.24 to 115909.92) | 893.77 (600.81 to 1331.07) | 70432.27 (53530.99 to 92551.47) | 811.33 (544.76 to 1206.21) | -0.57 (-0.73 to -0.42) |
| Ghana | 200357.43 (192325.19 to 209616.31) | 3777.36 (3104.36 to 4685.07) | 216558.5 (203637.7 to 229304.28) | 2585.92 (2192.26 to 3118.11) | -1.64 (-1.84 to -1.44) |
| Greece | 15611.56 (13151.06 to 19019.13) | 964.71 (756.8 to 1270.33) | 12939.1 (10747.01 to 15680.8) | 1060.5 (818.61 to 1430.7) | 0.05 (-0.04 to 0.14) |
| Greenland | 66.75 (58.13 to 77.59) | 607.96 (460.39 to 816.7) | 52.52 (44.47 to 64.34) | 575.22 (432.04 to 792.77) | -0.51 (-0.65 to -0.37) |
| Grenada | 266.42 (239.69 to 299.52) | 1543.57 (1175.78 to 2006.16) | 219 (194.89 to 246.15) | 1533.68 (1231.16 to 1930.1) | -0.47 (-0.65 to -0.3) |
| Guam | 734.74 (681.73 to 796.34) | 2267.16 (1834.07 to 2810.51) | 777.14 (713.19 to 853.47) | 2114.86 (1799.19 to 2534.19) | -0.65 (-0.82 to -0.48) |
| Guatemala | 61210.57 (57852.99 to 65323.86) | 1882.17 (1611.95 to 2256.48) | 40957.04 (37211.52 to 45438.3) | 995.2 (826.79 to 1215.49) | -2.4 (-2.65 to -2.15) |
| Guinea | 102418.26 (95695.14 to 109852.71) | 4105.02 (3343.35 to 5097.31) | 118942.41 (112160.43 to 126283.98) | 2643.28 (2229.14 to 3212.6) | -1.55 (-1.65 to -1.45) |
| Guinea-Bissau | 14723.8 (13923.26 to 15610.19) | 3866.95 (3135.05 to 4903.28) | 14715.05 (13790.14 to 15768.66) | 2532.78 (2102.93 to 3112.59) | -1.71 (-1.85 to -1.58) |
| Guyana | 5772.92 (5475.3 to 6101.41) | 3393.27 (2758.57 to 4236.62) | 2847.47 (2651.66 to 3055.09) | 2242.56 (1824.19 to 2793.82) | -1.76 (-1.94 to -1.58) |
| Haiti | 33213.59 (31287.12 to 35236.38) | 3735.53 (2540.24 to 5206.52) | 43482.43 (39718.08 to 47398.58) | 2434.8 (1760.72 to 3275.26) | -1.86 (-1.99 to -1.72) |
| Honduras | 21251.28 (19702.55 to 22984.99) | 1431.98 (1144.99 to 1810.13) | 20050.02 (18203.03 to 22126.25) | 891.53 (715.4 to 1112.76) | -2.42 (-2.62 to -2.22) |
| Hungary | 17245.89 (15226.95 to 19703.24) | 1306.9 (1084.36 to 1567.49) | 11952 (10563.37 to 13529.19) | 1334.05 (1010.38 to 1785.57) | -0.23 (-0.35 to -0.1) |
| Iceland | 604.15 (498.05 to 737.22) | 1235.89 (912.95 to 1702.98) | 635.15 (519.65 to 795.54) | 1232.25 (908.6 to 1700.15) | -0.1 (-0.19 to -0.01) |
| India | 21991229.28 (20791591.96 to 23514003.65) | 9355.98 (7402.1 to 12117.73) | 20155578.01 (18230982.41 to 22534162.47) | 8930.63 (6824.33 to 11518.57) | -0.38 (-0.52 to -0.24) |
| Indonesia | 1875115.41 (1727657.19 to 2083988.5) | 5643.57 (3932.03 to 7939.36) | 2059360.21 (1876040.97 to 2301151.59) | 4892.26 (3852.39 to 6309.56) | -0.76 (-1.04 to -0.49) |
| Iran (Islamic Republic of) | 416119.22 (380912.19 to 459985.04) | 2844.69 (2105.75 to 3851.09) | 478309.94 (436255.57 to 532339.92) | 2820.65 (2358.87 to 3469.31) | -0.36 (-0.51 to -0.21) |
| Iraq | 198535.53 (186369.99 to 212675.29) | 3434.08 (2780.45 to 4307.65) | 410027.35 (376688.44 to 446267.83) | 3536.64 (3073.41 to 4118.98) | -0.27 (-0.41 to -0.13) |
| Ireland | 9484.13 (7653.94 to 12069.27) | 1239.19 (914.11 to 1706.24) | 10697.24 (8459.61 to 13699.87) | 1318.29 (955.66 to 1842.12) | 0.03 (-0.05 to 0.11) |
| Israel | 15375.06 (12667.05 to 18927.3) | 1265.4 (933.31 to 1751.86) | 25656.65 (21134.4 to 31568.58) | 1254.52 (914.86 to 1752.44) | -0.32 (-0.43 to -0.21) |
| Italy | 147965.02 (121873.73 to 182285.02) | 1897.85 (1447.75 to 2517.45) | 109174.61 (87581.04 to 136375.3) | 1650.39 (1236 to 2219.1) | -0.89 (-1.04 to -0.73) |
| Jamaica | 10279.6 (9495.26 to 11349.15) | 1986.17 (1616.63 to 2454.53) | 5482.5 (4857.73 to 6276.72) | 1368.35 (1087.55 to 1735.72) | -1.6 (-1.92 to -1.28) |
| Japan | 288320.82 (260781.23 to 323633.81) | 1526.03 (1344.61 to 1786.3) | 201837.8 (182437.65 to 226799.07) | 1524.49 (1325.11 to 1807.05) | -0.3 (-0.44 to -0.16) |
| Jordan | 29741.4 (27596.73 to 32477.04) | 2336.95 (1908.04 to 2894.5) | 61384.13 (54925.58 to 69658.69) | 2362.78 (1851.2 to 3076.68) | -0.19 (-0.3 to -0.08) |
| Kazakhstan | 53554.23 (49075.69 to 57759.43) | 1523.43 (1202.45 to 1982.6) | 47696.95 (43288.8 to 52615.98) | 1558.58 (1154.64 to 2164.63) | 0.04 (-0.11 to 0.19) |
| Kenya | 190893.74 (177560.14 to 208579.57) | 3503.86 (2344.71 to 5203.51) | 278121.57 (257168.86 to 305199.97) | 3247.33 (2252.96 to 4560.27) | -0.88 (-1.04 to -0.71) |
| Kiribati | 898.62 (855.58 to 944.93) | 3990.89 (3344.08 to 4830.31) | 872.43 (822.2 to 923.08) | 2823 (2412.83 to 3334.54) | -1.2 (-1.27 to -1.13) |
| Kuwait | 7284.74 (6401.82 to 8344.52) | 1969.47 (1412.7 to 2761.7) | 12147.77 (10152.29 to 14542.77) | 2470.27 (1622.11 to 3730) | 0.26 (0.11 to 0.42) |
| Kyrgyzstan | 12987.6 (11704.14 to 14624.77) | 1281.9 (931.05 to 1757) | 16496.2 (14544.13 to 18944.39) | 1579.97 (1048.16 to 2362.04) | 0.33 (0.03 to 0.64) |
| Lao People's Democratic Republic | 39668.84 (36905.7 to 42663.37) | 6392.62 (3940.66 to 9442.73) | 35572.26 (32999.86 to 38923.9) | 3709.88 (2668.31 to 5058.58) | -2.07 (-2.2 to -1.94) |
| Latvia | 7273.49 (6700.5 to 7891.19) | 2063.21 (1588.55 to 2714.23) | 3968.56 (3632.45 to 4362.11) | 1972.82 (1501.25 to 2626.72) | 0.13 (-0.02 to 0.27) |
| Lebanon | 27130.18 (25403.18 to 29107.54) | 2740.44 (2263.31 to 3348) | 28292.15 (26033.52 to 30989.35) | 2643.44 (2123.87 to 3360.02) | -0.48 (-0.68 to -0.28) |
| Lesotho | 26125.16 (24892.87 to 27521.9) | 4458.64 (3822.65 to 5322.95) | 8854.77 (8117.31 to 9717.59) | 2449.71 (1881.61 to 3177.57) | -1.92 (-2.35 to -1.49) |
| Liberia | 16337.81 (15416.6 to 17340.63) | 3142.04 (2308.82 to 4319.93) | 25095.1 (23422.95 to 26776.93) | 1997.16 (1632.84 to 2454.39) | -2.32 (-2.51 to -2.12) |
| Libya | 36909.25 (34272.2 to 40202.25) | 2683.41 (2168.87 to 3367.85) | 27009.24 (24081.28 to 30762.18) | 2847.11 (2222.62 to 3714.38) | -0.1 (-0.24 to 0.03) |
| Lithuania | 11056.44 (10196.07 to 12085.82) | 2121.21 (1639.16 to 2810.47) | 5356.16 (4917.99 to 5888.48) | 1976.49 (1492.47 to 2678.92) | -0.07 (-0.21 to 0.08) |
| Luxembourg | 624.31 (534.41 to 743.25) | 1204.84 (920.83 to 1626.93) | 939.67 (780.89 to 1166.05) | 1223.02 (909.5 to 1686.17) | -0.08 (-0.18 to 0.02) |
| Madagascar | 201204.98 (191073.82 to 211731.35) | 5142.58 (4022.41 to 6702.98) | 263147.34 (248659.63 to 278600.22) | 3363.63 (2799.51 to 4065.56) | -1.6 (-1.71 to -1.5) |
| Malawi | 56336.59 (52332.77 to 60889.58) | 4450.75 (2783.89 to 6761.83) | 81818.17 (73661.83 to 90707.04) | 2986.81 (2055.76 to 4220.66) | -2.31 (-2.59 to -2.02) |
| Malaysia | 234977.05 (220539.09 to 253277.54) | 4357.21 (3723.57 to 5171.29) | 226432.12 (208599.31 to 247706.3) | 4372.27 (3409.06 to 5776.37) | -0.33 (-0.54 to -0.12) |
| Maldives | 4907.36 (4632.52 to 5223.14) | 8160.68 (5984.46 to 10708.32) | 6583.63 (5917.17 to 7377.09) | 7829.12 (6161.42 to 10096.71) | -0.88 (-1.08 to -0.69) |
| Mali | 197743.67 (188714.72 to 206701.79) | 5330.5 (4534.84 to 6484.73) | 406175.62 (386916.51 to 425123.66) | 4696.52 (3977.58 to 5660.14) | -0.66 (-0.82 to -0.51) |
| Malta | 924.27 (755.96 to 1138.79) | 1321.8 (987.74 to 1798.19) | 696.54 (572.85 to 866.24) | 1363.55 (1002.61 to 1883.21) | -0.09 (-0.2 to 0.02) |
| Marshall Islands | 320.68 (299.24 to 344.51) | 2241.87 (1825.75 to 2793.22) | 239.12 (223.07 to 257.24) | 1936.4 (1613.54 to 2362.85) | -0.81 (-1.01 to -0.6) |
| Mauritania | 32128.74 (30297.38 to 33996.07) | 4229.82 (3474.43 to 5263.85) | 36346.28 (34162.1 to 38749.91) | 3089.14 (2605.43 to 3715.8) | -1.72 (-1.92 to -1.53) |
| Mauritius | 12551.56 (11844.98 to 13358.67) | 4864.66 (4267.04 to 5649.9) | 7034.28 (6532.72 to 7618.64) | 4469.77 (3812.62 to 5289.59) | -0.84 (-1.02 to -0.66) |
| Mexico | 395828.17 (350402.08 to 456866.03) | 2141.87 (1479.29 to 3072.44) | 434558.15 (376595.66 to 508894.29) | 1846.82 (1420.41 to 2448.55) | -1.19 (-1.51 to -0.87) |
| Micronesia (Federated States of) | 674.84 (630.1 to 722.51) | 2320.98 (1854.95 to 2907.69) | 421.43 (393.96 to 451.96) | 1941.67 (1652.96 to 2320.68) | -1.16 (-1.36 to -0.96) |
| Monaco | 33.48 (28.03 to 40.68) | 1286.67 (937.84 to 1814.32) | 46.72 (37.81 to 59.28) | 1175.69 (862.96 to 1641.48) | -0.45 (-0.55 to -0.36) |
| Mongolia | 7731.49 (7109.06 to 8458.92) | 1354.38 (1086.02 to 1713.58) | 8103.27 (7212.53 to 9090.04) | 1386.57 (984.27 to 1960.56) | -0.03 (-0.33 to 0.27) |
| Montenegro | 1685.95 (1486.83 to 1937.83) | 1985.43 (1380.07 to 2842.12) | 1244.79 (1070.93 to 1461.91) | 2436.32 (1580.36 to 3690.28) | 0.66 (0.49 to 0.82) |
| Morocco | 191717.88 (177056.63 to 208891.35) | 2720.39 (2199.59 to 3413.19) | 186410.48 (168133.19 to 208549.44) | 2686.45 (2170.64 to 3386.31) | -0.51 (-0.69 to -0.33) |
| Mozambique | 172536.64 (165400.47 to 179675.86) | 3552.73 (2942.57 to 4356.91) | 141518.37 (132114.13 to 151343.51) | 1566.29 (1266.88 to 1946.14) | -3.43 (-3.65 to -3.2) |
| Myanmar | 305570.11 (285683.63 to 325255.49) | 4007.39 (3043.7 to 5188.27) | 270127.38 (250401.52 to 292879.75) | 2931.04 (2347.7 to 3701.37) | -1.27 (-1.55 to -0.98) |
| Namibia | 13800.07 (13038.87 to 14592.18) | 3303.85 (2619.62 to 4207.58) | 14253.38 (13266.83 to 15292.59) | 2763.54 (2170.7 to 3570.84) | -0.44 (-0.78 to -0.09) |
| Nauru | 82.95 (77.61 to 88.53) | 2403.6 (1936.84 to 2977.76) | 62.91 (58.61 to 67.99) | 2198.24 (1833.78 to 2692.53) | -0.83 (-1.09 to -0.57) |
| Nepal | 314095.88 (295826.85 to 333854.82) | 5720.29 (4190.8 to 7901.38) | 234329.38 (217129.97 to 254862.95) | 4085 (3215.41 to 5265.4) | -0.99 (-1.14 to -0.85) |
| Netherlands | 24595.4 (20992.54 to 29709.03) | 1172.5 (874.9 to 1614.79) | 26766.82 (22057.25 to 33544.66) | 1258.55 (929.46 to 1751.63) | 0 (-0.1 to 0.09) |
| New Zealand | 9758.23 (8890.73 to 10775.73) | 1290 (1123.57 to 1499.99) | 9939.26 (8868.53 to 11277.88) | 1227.19 (1057.33 to 1452.53) | -0.24 (-0.38 to -0.09) |
| Nicaragua | 23372.4 (22082.46 to 24817.39) | 1607.66 (1345.43 to 1959.11) | 13265.86 (11879.39 to 14981.41) | 987.87 (797.11 to 1220.2) | -2.09 (-2.35 to -1.83) |
| Niger | 216535.38 (207842.53 to 224810.79) | 6001.27 (5143.66 to 7260.88) | 466316.28 (444502.8 to 490658.38) | 4258.27 (3732.56 to 4921.72) | -1.62 (-1.75 to -1.49) |
| Nigeria | 940252.24 (889181.44 to 1004987.96) | 4603.62 (3222.15 to 6513.14) | 2564895.88 (2397488.36 to 2773427.99) | 3990.33 (3155.34 to 5095.45) | -0.75 (-0.98 to -0.51) |
| Niue | 13.5 (12.5 to 14.57) | 2245.27 (1898.74 to 2698.97) | 6.34 (5.84 to 6.95) | 2227.39 (1861.54 to 2701.31) | -0.38 (-0.49 to -0.27) |
| North Macedonia | 5029.8 (4256.61 to 5984.95) | 2218.92 (1462.35 to 3218.25) | 3873.68 (3252.06 to 4697.35) | 2438.46 (1528.97 to 3752.5) | 0.61 (0.45 to 0.76) |
| Northern Mariana Islands | 243.35 (227.74 to 262.12) | 2212.55 (1887.75 to 2622.21) | 117.87 (108.67 to 129.35) | 2245.94 (1860.38 to 2743.27) | -0.46 (-0.66 to -0.25) |
| Norway | 10915.02 (9049.24 to 13214.61) | 1656.77 (1236.06 to 2231.2) | 12212.69 (9815.68 to 15323.68) | 1592.63 (1177.49 to 2188.04) | -0.6 (-0.79 to -0.4) |
| Oman | 25625.72 (24130.47 to 27406.76) | 3675.19 (3139.65 to 4414.21) | 30320.22 (27738.27 to 33062.46) | 3687.54 (2942.08 to 4716.42) | -0.28 (-0.4 to -0.17) |
| Pakistan | 2195319.92 (2066788.07 to 2341106.81) | 6179.52 (4770.13 to 8213.51) | 3585016.59 (3358226.18 to 3852202.75) | 5625.62 (4601.87 to 7020.38) | -0.95 (-1.14 to -0.76) |
| Palau | 88.29 (82.02 to 95.65) | 2305.55 (1988.56 to 2734.33) | 54.85 (49.8 to 60.96) | 2294.46 (1937.29 to 2780.23) | -0.34 (-0.48 to -0.2) |
| Palestine | 15840.3 (14856.51 to 17013.27) | 1972.39 (1644.62 to 2390.11) | 24774.7 (22532.92 to 27615.94) | 2008.43 (1535.42 to 2678.11) | -0.36 (-0.58 to -0.15) |
| Panama | 9518.11 (8876.25 to 10225.2) | 1452.74 (1239.69 to 1716.46) | 9623.08 (8625.55 to 10907.47) | 1116.81 (913.31 to 1381.63) | -1.45 (-1.6 to -1.3) |
| Papua New Guinea | 45003.55 (42670.3 to 47866.31) | 4820.29 (3548.41 to 6570.88) | 85285.3 (80041.75 to 90912.47) | 4217.04 (3126.05 to 5657.31) | -0.47 (-0.8 to -0.13) |
| Paraguay | 6196.89 (5380.01 to 7284.01) | 891.46 (601.24 to 1266.12) | 7449.23 (6510.71 to 8776.39) | 811.78 (573.55 to 1100.4) | 0.03 (-0.31 to 0.37) |
| Peru | 38792.27 (35352.08 to 42820.14) | 725.46 (535.42 to 978.25) | 29224.9 (26121.06 to 33288.46) | 428.94 (344.44 to 534.57) | -2.39 (-2.57 to -2.21) |
| Philippines | 502942.26 (461500 to 549924.2) | 4416.27 (2966.61 to 6428.48) | 807775.96 (745135.8 to 881435.61) | 3004.53 (2446.67 to 3779.46) | -1.57 (-1.72 to -1.41) |
| Poland | 107641.63 (94018.87 to 124271.44) | 1980.04 (1404.39 to 2796.65) | 71724.19 (62248.53 to 83894.98) | 2106.21 (1476.01 to 3022.56) | 0.13 (-0.1 to 0.36) |
| Portugal | 22095.24 (18022.59 to 27455.19) | 1551.3 (1125.49 to 2138.62) | 15318.84 (12160.16 to 19605.72) | 1419.84 (1034.71 to 1989.23) | -0.12 (-0.33 to 0.08) |
| Puerto Rico | 9485.7 (8559.8 to 10652.48) | 1792.67 (1290.23 to 2518.38) | 3990.33 (3425.44 to 4750.44) | 2053.69 (1346.82 to 3125.66) | -0.05 (-0.32 to 0.22) |
| Qatar | 2575.57 (2368.39 to 2822.37) | 2608.15 (2061.2 to 3341.28) | 7131.53 (6300.45 to 8124.74) | 2523.06 (1917.89 to 3368) | -0.22 (-0.34 to -0.11) |
| Republic of Korea | 63611.82 (55533.66 to 74162.61) | 717.94 (578.72 to 916) | 41258.68 (34749.39 to 50269.9) | 746.77 (575.72 to 996.92) | -0.02 (-0.11 to 0.06) |
| Republic of Moldova | 13456.79 (12225.2 to 14814.03) | 1769.8 (1331.74 to 2422.16) | 6094.64 (5315.11 to 7014.17) | 2005.35 (1415.14 to 2929.81) | 0.52 (0.4 to 0.63) |
| Romania | 67343.54 (60579.36 to 75857.9) | 1935.4 (1463.65 to 2611.76) | 35680.41 (31219.25 to 41289.74) | 2147.33 (1518.08 to 3074.92) | 0.29 (0.1 to 0.48) |
| Russian Federation | 468257.38 (427548.87 to 514139.29) | 1917.38 (1527.36 to 2480.09) | 392849.18 (355152.48 to 436337.8) | 2250.96 (1683.22 to 3033.18) | 0.4 (0.14 to 0.67) |
| Rwanda | 69718.27 (65572.34 to 74185.76) | 2876.01 (2304.34 to 3631.11) | 54445.55 (50603.11 to 58701.28) | 1907.07 (1479.38 to 2436.67) | -2.31 (-2.63 to -1.99) |
| Saint Kitts and Nevis | 117.27 (105.32 to 131.77) | 1555.71 (1192.38 to 2008.4) | 102.96 (92.25 to 117.49) | 1506.33 (1170.41 to 1928.03) | -0.43 (-0.59 to -0.27) |
| Saint Lucia | 395.61 (354.91 to 442.64) | 1477.5 (1089.96 to 1955.58) | 266.56 (235.14 to 305.43) | 1390.46 (1099.19 to 1749.24) | -0.5 (-0.68 to -0.31) |
| Saint Vincent and the Grenadines | 318.97 (285.15 to 358.9) | 2022.89 (1465.05 to 2753.02) | 203.95 (182.23 to 230.08) | 1587.06 (1206.11 to 2046.42) | -1.12 (-1.28 to -0.97) |
| Samoa | 385.38 (336.39 to 450.74) | 1130.83 (842.17 to 1526.89) | 513.19 (450.19 to 599.42) | 1114.3 (873 to 1442.69) | -0.42 (-0.67 to -0.18) |
| San Marino | 41.02 (33.76 to 50.66) | 1155.87 (865.89 to 1568.84) | 51.67 (41.95 to 64.95) | 1246.06 (931.35 to 1709.57) | -0.01 (-0.12 to 0.1) |
| Sao Tome and Principe | 1158.81 (1088.05 to 1232.44) | 3030.03 (2411.95 to 3888.52) | 1252.78 (1168.45 to 1343.24) | 2323.76 (1988.78 to 2755.36) | -1.81 (-2.02 to -1.59) |
| Saudi Arabia | 115417.36 (106051.02 to 125639.72) | 2688.75 (2079.97 to 3456.24) | 113903.87 (102958.58 to 126797.34) | 2610.08 (1961.7 to 3586.04) | -0.33 (-0.5 to -0.16) |
| Senegal | 100549.52 (95129.1 to 106393.18) | 3283.91 (2744.42 to 3978.89) | 108859.74 (101711.9 to 116738.26) | 2346.74 (1979.24 to 2836.05) | -1.18 (-1.27 to -1.1) |
| Serbia | 22056.57 (19267.49 to 25554.72) | 2148.6 (1530.46 to 3057.14) | 17362.17 (15188.23 to 20216.69) | 2110.06 (1503.32 to 2985.72) | -0.25 (-0.38 to -0.13) |
| Seychelles | 359.66 (335.03 to 392.21) | 2237.19 (1815.15 to 2786.87) | 335.48 (308.53 to 369.25) | 2442.54 (1882.65 to 3171.8) | 0.01 (-0.11 to 0.13) |
| Sierra Leone | 45952.04 (43959.98 to 47957.59) | 4353 (3255.62 to 5831.69) | 62339.15 (58776.74 to 66408.89) | 2882.47 (2325.19 to 3638.2) | -1.66 (-1.77 to -1.56) |
| Singapore | 11778.34 (10902.73 to 12869.62) | 2133.65 (1897.41 to 2438.82) | 15505.35 (14273.11 to 17089.3) | 2027.22 (1779.82 to 2359.39) | -0.16 (-0.21 to -0.11) |
| Slovakia | 12514.15 (11077.24 to 14365.4) | 1796.12 (1259.37 to 2582.32) | 8510.33 (7433.26 to 9833.16) | 1861.63 (1274.67 to 2736.01) | 0.17 (0.02 to 0.32) |
| Slovenia | 3680.6 (3211.85 to 4293.22) | 1754.37 (1207.14 to 2549.53) | 3293.33 (2839.25 to 3877.87) | 1946.04 (1346.91 to 2829.65) | 0.36 (0.21 to 0.5) |
| Solomon Islands | 2378.45 (2222.57 to 2551.08) | 3016.36 (2190.36 to 4162.46) | 3519.1 (3272.84 to 3785.3) | 2238.28 (1731.82 to 2877.33) | -1.42 (-1.59 to -1.24) |
| Somalia | 161734.25 (152976.41 to 171888.16) | 6296.18 (5050.8 to 7968.25) | 323074.47 (303073.69 to 349089.58) | 4665.05 (3758.2 to 5787.46) | -1.42 (-1.54 to -1.29) |
| South Africa | 196964.41 (185723.08 to 211684.81) | 2270.64 (1717.85 to 3149.78) | 178784.64 (166824.66 to 194348.27) | 2038.21 (1543.73 to 2725.62) | -1.02 (-1.2 to -0.84) |
| South Sudan | 103060.29 (97884.59 to 108552.54) | 5168.93 (4184.45 to 6579.48) | 123111.81 (116681.6 to 129675.26) | 4147.19 (3412.01 to 5105.13) | -1.03 (-1.17 to -0.89) |
| Spain | 73876.99 (57713.64 to 95520.51) | 1267.58 (910.74 to 1785.29) | 65400.4 (51332.33 to 83333.57) | 1263.19 (909.62 to 1767.21) | -0.17 (-0.33 to -0.01) |
| Sri Lanka | 246622.12 (228162.03 to 270866.65) | 6661.72 (5543.31 to 7974.03) | 265970.47 (231922.17 to 306931.34) | 8376.65 (6303.97 to 10559.73) | 0.3 (-0.01 to 0.62) |
| Sudan | 394339.75 (379310.26 to 410219.6) | 7483.51 (5533.29 to 10083.52) | 657859.44 (615733.67 to 705963.27) | 5612.42 (4728.58 to 6835.75) | -1.4 (-1.75 to -1.05) |
| Suriname | 1148.41 (1053.56 to 1259.35) | 2176.64 (1627.41 to 2927.65) | 1661.32 (1522.91 to 1820.87) | 1957.8 (1545.65 to 2484.56) | -0.95 (-1.13 to -0.77) |
| Sweden | 20928.22 (17650.14 to 25125.23) | 1636.52 (1226.31 to 2196.78) | 24208.81 (19570.84 to 29997.95) | 1621.98 (1195.79 to 2224.24) | -0.21 (-0.43 to 0.01) |
| Switzerland | 11300.99 (9306.51 to 14051.86) | 1245.85 (911.44 to 1743.96) | 12533.54 (10346.48 to 15578.32) | 1222.79 (900.7 to 1683.91) | -0.27 (-0.37 to -0.17) |
| Syrian Arab Republic | 195604.61 (182934.24 to 210352.78) | 4001.81 (3369.52 to 4775.16) | 116370.79 (106114.62 to 128620.67) | 4088.04 (3420.66 to 5026.7) | -0.23 (-0.33 to -0.13) |
| Taiwan (Province of China) | 29706.65 (25474.98 to 35697.56) | 1273.5 (835.22 to 1885.21) | 18111.42 (13963.32 to 23888.55) | 1892.14 (1033.04 to 3128.71) | 1.55 (1.26 to 1.83) |
| Tajikistan | 55734.31 (52466.46 to 58849.01) | 2784.82 (2316.65 to 3436.33) | 65045.83 (60729.75 to 69809.26) | 2601.69 (2109.88 to 3299.08) | -0.33 (-0.45 to -0.21) |
| Thailand | 330179.45 (308673.27 to 354471.52) | 2648.56 (2246.2 to 3167.92) | 187830.37 (172349.23 to 205300.77) | 2559.34 (2114 to 3212.96) | -0.26 (-0.39 to -0.14) |
| Timor-Leste | 16414.05 (15655.29 to 17180.07) | 8513.27 (5882.06 to 12107.22) | 16739.94 (15785.02 to 17700.2) | 5465.55 (4296.16 to 6939.83) | -1.94 (-2.15 to -1.74) |
| Togo | 43044.85 (40727.07 to 45516.83) | 3608.24 (2791.49 to 4754.24) | 54785.11 (51394.85 to 58459.03) | 2542.4 (2047.21 to 3215.33) | -1.53 (-1.68 to -1.38) |
| Tokelau | 13.44 (12.56 to 14.39) | 2318.24 (1921.4 to 2850.83) | 8.39 (7.82 to 9.03) | 2102.88 (1824.01 to 2476.21) | -0.55 (-0.65 to -0.45) |
| Tonga | 544.56 (504.58 to 592.49) | 1971.01 (1634.09 to 2451.62) | 529.82 (491.35 to 575.58) | 1886.21 (1618.53 to 2246.19) | -0.48 (-0.61 to -0.36) |
| Trinidad and Tobago | 4408.22 (4061.9 to 4787.7) | 1579.01 (1314.22 to 1950.85) | 3016.09 (2719.74 to 3393.74) | 1610.89 (1313.81 to 1993.35) | -0.38 (-0.53 to -0.24) |
| Tunisia | 50295.88 (46230.57 to 55571.9) | 2611.57 (1961.21 to 3436.85) | 42796.46 (38199.81 to 48813.66) | 2450.8 (1858.65 to 3299.36) | -0.34 (-0.47 to -0.22) |
| Turkey | 272333.01 (247983.57 to 298179.92) | 2004.12 (1554.36 to 2600.95) | 187924.15 (170873.96 to 208902.71) | 1661.9 (1362.07 to 2060.97) | -1.07 (-1.29 to -0.84) |
| Turkmenistan | 26888.49 (25270.61 to 28628.34) | 2420.82 (1899.84 to 3168.15) | 22730.05 (21059.46 to 24681.09) | 2204.21 (1708.96 to 2927.46) | -0.42 (-0.54 to -0.3) |
| Tuvalu | 58.13 (54.41 to 62.27) | 2891.47 (2176.81 to 3792.75) | 48.04 (44.32 to 52.56) | 2019.23 (1695.03 to 2451.38) | -1.57 (-1.74 to -1.4) |
| Uganda | 135532.37 (127070.34 to 144768.24) | 2664.23 (1933.97 to 3628.21) | 174279.1 (162160.62 to 186857.98) | 1650.78 (1274.69 to 2136.71) | -1.85 (-1.97 to -1.72) |
| Ukraine | 219629.77 (202822.01 to 239608.64) | 2593.71 (2143.19 to 3241.79) | 136373.1 (124759.63 to 149096.53) | 2883.4 (2318.48 to 3684.15) | 0.38 (0.19 to 0.57) |
| United Arab Emirates | 18050.49 (17026.36 to 19233.93) | 3493.32 (2992.54 to 4188.87) | 29573.52 (26882.25 to 32794.1) | 3502.44 (2959.61 to 4263.07) | -0.25 (-0.4 to -0.1) |
| United Kingdom | 160870.86 (132086.46 to 195848.94) | 1799.85 (1328.05 to 2454.78) | 189512.45 (151361.08 to 237907.19) | 1974.03 (1425.65 to 2760.9) | -0.03 (-0.27 to 0.2) |
| United Republic of Tanzania | 241032.99 (230700.09 to 251840.24) | 2803.64 (2205.63 to 3571.81) | 290080.11 (272379.15 to 306949.64) | 1764.26 (1401.63 to 2220.74) | -1.95 (-2.09 to -1.81) |
| United States of America | 292772 (228113.82 to 378308.43) | 643.2 (456.02 to 917.19) | 332930.22 (249902.33 to 436956.33) | 683.31 (468.8 to 993.19) | -0.3 (-0.48 to -0.13) |
| United States Virgin Islands | 276 (251.69 to 305.72) | 1496.18 (1180.39 to 1906.18) | 190.1 (171.76 to 213.34) | 1417.89 (1142.72 to 1770.89) | -0.4 (-0.6 to -0.19) |
| Uruguay | 5929.3 (5286.17 to 6833.24) | 991.79 (814.37 to 1208.85) | 5705.99 (5055.66 to 6546.78) | 999.39 (829.04 to 1226.93) | -0.46 (-0.66 to -0.27) |
| Uzbekistan | 188072.7 (176365.11 to 200014.76) | 2681.01 (2244.2 to 3208.17) | 204490.44 (191376.72 to 219111.93) | 2527.39 (2123.83 to 3072.81) | -0.46 (-0.57 to -0.36) |
| Vanuatu | 908.05 (851.5 to 969.02) | 2266.28 (1759.17 to 2917.48) | 1335.27 (1236.08 to 1435.96) | 1956.12 (1556.15 to 2469.57) | -0.75 (-0.95 to -0.55) |
| Venezuela (Bolivarian Republic of) | 93989.97 (86556.18 to 102997.7) | 1828.59 (1504.63 to 2251.05) | 105557.61 (95086.62 to 118978.05) | 1886.96 (1580.22 to 2273.05) | -0.08 (-0.32 to 0.17) |
| Viet Nam | 875666.73 (816854.01 to 947387.6) | 4434.45 (3684.63 to 5385.19) | 655645.67 (587650.39 to 735660.59) | 4440.8 (3443.78 to 5953.8) | -0.24 (-0.44 to -0.04) |
| Yemen | 333058.47 (308800.07 to 363293.53) | 8124.6 (5658.45 to 11611.85) | 484021.38 (449510.22 to 523934.75) | 5899.95 (4560.89 to 7710.16) | -1.08 (-1.2 to -0.97) |
| Zambia | 55275.85 (52695.78 to 57907.86) | 2814.89 (1969.08 to 3919.32) | 92378.81 (86923.01 to 98189.97) | 2061.5 (1589.08 to 2705.85) | -1.18 (-1.34 to -1.01) |
| Zimbabwe | 75252.33 (70715.89 to 80577.53) | 2461.25 (1897.68 to 3188.5) | 72243.77 (66420.99 to 78428.91) | 2150.18 (1605.3 to 2863.36) | 0.13 (-0.12 to 0.38) |

**Supplementary Table 4** Prevalence of nutritional deficiency among children in 1990 and 2019 and the EAPC from 1990 to 2019 in different countries and territories

| Location | 1990 |  | 2019 |  | 1990-2019 |
| --- | --- | --- | --- | --- | --- |
|  | Prevalence cases NO.(95%UI) | ASPR/100,000 (95% CI) | Prevalence cases NO.(95%UI) | ASPR/100,000 (95% CI) | EAPC (95%CI) |
| Yemen | 2253829.69 (2066862.57 to 2429727.23) | 29187.47 (25518.99 to 33264.26) | 4480369.69 (4080712.92 to 4835218.66) | 34761.67 (30394.37 to 39403.12) | 0.77 (0.67 to 0.87) |
| Burkina Faso | 1787906.93 (1622946.09 to 1952023.83) | 36475 (31150.61 to 42296.11) | 4671720.56 (4140199.51 to 5240798.02) | 43932.08 (37270.73 to 51256.92) | 0.75 (0.67 to 0.82) |
| Zimbabwe | 1103043.89 (939051.23 to 1268163.86) | 22522.99 (16846.21 to 28806.76) | 1480794.33 (1278863.16 to 1688089.62) | 24713.59 (19161.07 to 30677.19) | 0.59 (0.42 to 0.76) |
| Haiti | 830110.75 (738820.78 to 922919.64) | 30257.94 (24616.7 to 36057.67) | 1459693.2 (1303548.7 to 1637953.26) | 34014.07 (27574.86 to 40506.48) | 0.54 (0.5 to 0.58) |
| Mali | 1750813.81 (1640446.57 to 1874373.71) | 41139.28 (36196.74 to 45841.1) | 4917709.31 (4506938.8 to 5294659.74) | 46810.81 (41061.15 to 52210.21) | 0.46 (0.35 to 0.56) |
| Guinea-Bissau | 172751.05 (153786.53 to 193989.18) | 35159.83 (28796.53 to 41285.77) | 302460.38 (270460.31 to 334451.05) | 37691.06 (30748.37 to 44543.76) | 0.42 (0.32 to 0.51) |
| Bolivia (Plurinational State of) | 581605.31 (500167.98 to 661555.88) | 21059.46 (16364.51 to 26021.36) | 857818.01 (726898.47 to 988637.21) | 21027.32 (15906.67 to 26864.37) | 0.32 (0.2 to 0.44) |
| Sierra Leone | 591699.55 (522436.06 to 657208.67) | 35872.86 (29343.64 to 42053.6) | 1282216.61 (1170334.05 to 1390836.64) | 38004.23 (32889.4 to 42903.13) | 0.24 (0.21 to 0.27) |
| Ghana | 2098856.86 (1878881.38 to 2320914.94) | 29475.49 (24663.85 to 34439.55) | 3518053.06 (3102995.08 to 3977374.6) | 31083.27 (25131.57 to 37528.36) | 0.22 (0.14 to 0.31) |
| Fiji | 65988.35 (58075.52 to 74155.26) | 23596.54 (18832.8 to 28539.67) | 62014.16 (52210.44 to 72221.42) | 23584.95 (17361.37 to 30095.21) | 0.14 (0.07 to 0.21) |
| Vanuatu | 12253.46 (10361.47 to 14350.76) | 17395.84 (12902.82 to 22441.15) | 20870.89 (17245.14 to 24470) | 18985.43 (13857.21 to 24413.4) | 0.14 (0.05 to 0.23) |
| Bhutan | 119589.1 (89009.54 to 135343.7) | 46905.22 (33706.26 to 55106.87) | 96738.2 (88779.47 to 104710.74) | 50398.46 (43741.28 to 57065.8) | 0.12 (0.05 to 0.2) |
| Angola | 1247459.63 (1107778.67 to 1398743.52) | 25617.35 (20799.91 to 30739.22) | 3718200.26 (3220555.56 to 4255397.26) | 26133.99 (20597.65 to 32152.07) | 0.1 (0.05 to 0.16) |
| Zambia | 1444069.74 (1329146.77 to 1555227.67) | 37839.04 (32898.15 to 42554.25) | 2979635.37 (2751830.49 to 3219032.92) | 38418.92 (32964.49 to 43815.13) | 0.1 (0.06 to 0.14) |
| Burundi | 820947.62 (713460.24 to 924237.32) | 29554.73 (23428.95 to 35984.75) | 1537960.83 (1357672.36 to 1734671.62) | 27613.91 (22281.81 to 33256.24) | 0.06 (-0.05 to 0.16) |
| Cameroon | 1364189.95 (1218468 to 1507470.25) | 27055.84 (22209.23 to 32071.46) | 3258059.92 (2804917.21 to 3742655.07) | 27013.59 (21257.5 to 33157.56) | 0.06 (-0.02 to 0.15) |
| Guinea | 968688.68 (888152.56 to 1048160.24) | 32207.35 (27630.48 to 36854) | 1910015.4 (1712884.57 to 2094951.93) | 32613.72 (27158.82 to 37810.59) | 0.06 (0 to 0.13) |
| Sao Tome and Principe | 19158.16 (13403.07 to 22463.9) | 33201.31 (22240.76 to 41928.56) | 21892.62 (18047.63 to 25819.55) | 30402.3 (22046.6 to 38802.26) | 0.01 (-0.44 to 0.47) |
| Papua New Guinea | 456737.91 (406188.9 to 508842.12) | 26433.84 (21267.83 to 31574.95) | 977071.64 (857597.58 to 1098644.45) | 25434.48 (20229.12 to 30997.68) | -0.02 (-0.06 to 0.02) |
| Congo | 382181.42 (340814.23 to 425117.79) | 34613.22 (28541.32 to 40800.31) | 660675.7 (567200.36 to 750596.63) | 33163.67 (26000.08 to 40237.73) | -0.03 (-0.09 to 0.04) |
| Guatemala | 726743.29 (622620.49 to 832145.48) | 19203.17 (14463.33 to 24359.73) | 1032152.55 (862698.71 to 1218102.42) | 17879.7 (12854.72 to 23336.35) | -0.04 (-0.22 to 0.14) |
| Togo | 678655.84 (614640.51 to 743552.51) | 37088.01 (31843.8 to 42701.66) | 1168750 (1060524.51 to 1273202.83) | 36924.39 (31345.55 to 42443.22) | -0.04 (-0.18 to 0.1) |
| Benin | 779694.48 (703095.56 to 859885.96) | 30371.37 (25594.04 to 35240.23) | 1809630.9 (1607595.75 to 2019379.79) | 30572.9 (24621.05 to 36785.25) | -0.05 (-0.17 to 0.08) |
| Nigeria | 15550184.74 (14650781.64 to 16551768.02) | 38356.96 (34604.83 to 42122.72) | 35708841.69 (32285587.21 to 39051225.33) | 37905.2 (32366.17 to 43288.96) | -0.05 (-0.1 to 0) |
| United States of America | 2530061.54 (2065079.79 to 3004328.76) | 4541.13 (3217.65 to 6210.86) | 2538667.94 (1891871.11 to 3328677.41) | 4318.58 (2633.58 to 6676.07) | -0.08 (-0.43 to 0.27) |
| Chad | 1211686.63 (1109881.8 to 1321549.37) | 39418.71 (33545.78 to 45293.93) | 3316480.04 (2990773.51 to 3639809.45) | 38486.62 (32139.92 to 44897.43) | -0.1 (-0.11 to -0.08) |
| Central African Republic | 453622.77 (386520.36 to 507978.71) | 35545.81 (28438.05 to 41931.75) | 806709.33 (684118.94 to 922452.64) | 35478.16 (27649.86 to 42696.19) | -0.12 (-0.2 to -0.04) |
| Democratic Republic of the Congo | 6983434.78 (6442494.56 to 7542091.49) | 37718.21 (32759.63 to 42674.9) | 13532444.42 (12181613.61 to 14838942.07) | 35739.01 (30260.83 to 41256.23) | -0.16 (-0.24 to -0.07) |
| Lesotho | 234352.72 (210968.77 to 257744.05) | 30706.7 (25725.76 to 36089.88) | 172647.18 (152093.72 to 194178.22) | 26594.71 (21579.45 to 31868.7) | -0.16 (-0.28 to -0.05) |
| Armenia | 170821.02 (145361.09 to 199727.21) | 16162.16 (11945.16 to 20966.3) | 90388.22 (73971.79 to 108215.42) | 14971.37 (10684.01 to 20027.24) | -0.19 (-0.26 to -0.13) |
| Solomon Islands | 42042.93 (36337.45 to 47365.98) | 25895.19 (20188.6 to 31543.42) | 63454.04 (53895 to 72698.72) | 24060.37 (17969.26 to 30422.44) | -0.2 (-0.25 to -0.16) |
| El Salvador | 340229.67 (274659.59 to 408870.83) | 16093.17 (11329.83 to 21546.04) | 234433.71 (183334.29 to 290711.91) | 13871.99 (9249.67 to 19415.42) | -0.21 (-0.36 to -0.07) |
| Guam | 8154.03 (6636.44 to 9707.2) | 18618.53 (13144.66 to 25231.49) | 7616.81 (6130.07 to 9476.52) | 16747.01 (11453.83 to 23262.28) | -0.21 (-0.35 to -0.07) |
| Niger | 1770766.72 (1594008.15 to 1920815.41) | 42167.2 (35748.72 to 48232.29) | 4994775.65 (4484015.26 to 5462625.04) | 40301.21 (33711.42 to 46310.54) | -0.23 (-0.3 to -0.16) |
| Kiribati | 8302.38 (7409.06 to 9236.17) | 27112.62 (21823.85 to 32526.47) | 10496.61 (9106.02 to 12073.22) | 24989.29 (19390.85 to 31186.35) | -0.24 (-0.27 to -0.22) |
| Nepal | 2566973.51 (2276254.69 to 2862066.12) | 28758.91 (23240.3 to 34577.59) | 2510726.07 (2165015.78 to 2867333.76) | 27964.02 (21983.45 to 34734.94) | -0.24 (-0.3 to -0.18) |
| Eswatini | 86539.78 (72620.79 to 99507.24) | 21919.89 (16069.34 to 28076.71) | 80858.5 (66909.3 to 95064.38) | 19862.16 (14185.89 to 26391.25) | -0.25 (-0.35 to -0.15) |
| Mauritania | 397054.83 (364898.76 to 429737.05) | 40913.87 (34755 to 47148.72) | 583094.36 (447863.37 to 661275.07) | 36606.78 (26906.11 to 44126.12) | -0.26 (-0.29 to -0.23) |
| Samoa | 12903.43 (10695.96 to 15402) | 20294.72 (14770.36 to 26472.2) | 12598.87 (10120.17 to 15460.82) | 18274.14 (12573.86 to 24732.79) | -0.27 (-0.34 to -0.2) |
| Somalia | 1359191.05 (1253380.45 to 1480187.93) | 38787.84 (33237 to 44335.72) | 3537147.17 (3228651.86 to 3843422.08) | 36428.4 (30793.42 to 42300.81) | -0.27 (-0.29 to -0.25) |
| South Sudan | 915180.97 (811981.78 to 1010628.18) | 34350.98 (28213.55 to 40530.84) | 1356289.59 (1165196.03 to 1541398.06) | 32275.8 (25151.77 to 39264.7) | -0.28 (-0.31 to -0.24) |
| Gabon | 147418.17 (129284.13 to 166255.2) | 34717.76 (27459.61 to 41752.89) | 183076.43 (150345.01 to 212516.58) | 31496.07 (24111.09 to 38969.86) | -0.3 (-0.44 to -0.16) |
| Gambia | 224468.81 (209724.43 to 237715.28) | 46396.05 (41196.66 to 51410.03) | 414588.85 (380952.67 to 446623.96) | 44726 (38826.37 to 50238.96) | -0.3 (-0.36 to -0.23) |
| Mozambique | 2155810.04 (1958437.11 to 2355538.13) | 34607.06 (29234.58 to 39779.45) | 4446272.01 (3992964.27 to 4927962.76) | 31622 (26312.36 to 36987.06) | -0.3 (-0.33 to -0.27) |
| Myanmar | 5323205.57 (4702016.26 to 5984531.34) | 33375.3 (26806.93 to 40207.07) | 4519552.18 (3973339.47 to 5085156.88) | 30712.1 (24749.25 to 37307.47) | -0.3 (-0.31 to -0.28) |
| Saint Vincent and the Grenadines | 9043.43 (7294.61 to 10961.92) | 22194.27 (15135.34 to 30003.76) | 4893.03 (3863.06 to 6018.68) | 20309.71 (13162.02 to 27975.44) | -0.31 (-0.38 to -0.25) |
| Senegal | 1629073.83 (1511639.69 to 1743540.81) | 43763.49 (37997.64 to 49181.72) | 2449310.45 (2227821.57 to 2640482.77) | 40073.16 (34780.09 to 45273.94) | -0.32 (-0.37 to -0.27) |
| Belize | 18769.74 (15373.64 to 22334.85) | 23084.53 (16520.04 to 30628.44) | 24455.2 (19318.61 to 30090.2) | 20781.01 (13720.73 to 28829.82) | -0.34 (-0.39 to -0.29) |
| Suriname | 30954.15 (25403.65 to 36652.95) | 23816.86 (16901.22 to 31485.43) | 30440.93 (24386.13 to 36572.32) | 21421.17 (14652.15 to 29278.25) | -0.34 (-0.35 to -0.33) |
| Pakistan | 18492637.28 (16635909.35 to 20172589.53) | 35589.51 (29131.86 to 41957.13) | 28961020.89 (25518002.22 to 32338959.28) | 33222.52 (26907 to 39911.97) | -0.35 (-0.46 to -0.24) |
| Panama | 102205.6 (80599.89 to 128587.07) | 12306.61 (8177.31 to 17537.54) | 119931.88 (89612.76 to 160788.83) | 10630.27 (6421.33 to 16241.9) | -0.35 (-0.5 to -0.19) |
| Bahamas | 16184.35 (12934.55 to 19797.67) | 20650.66 (14099.62 to 28319.83) | 13846.78 (10708.99 to 17157.39) | 18667.52 (12052.59 to 26360.73) | -0.36 (-0.44 to -0.28) |
| Malawi | 1632767.72 (1478948.99 to 1781981.84) | 35957.34 (30321.87 to 41525.38) | 2635718.74 (2361643.62 to 2930459.96) | 33809.3 (27981.63 to 39655.72) | -0.36 (-0.46 to -0.27) |
| Northern Mariana Islands | 2386.87 (1943.29 to 2827.14) | 18099.06 (12639.06 to 24444.01) | 1051.18 (829.24 to 1295.01) | 15422.81 (10658.05 to 21352.4) | -0.37 (-0.49 to -0.25) |
| Tonga | 7136.59 (5954.19 to 8398.1) | 18358.96 (13451.29 to 23580.9) | 5716.36 (4675.6 to 6918.7) | 16183.73 (11311.02 to 21477.52) | -0.37 (-0.41 to -0.33) |
| Ethiopia | 8747709.32 (8337621.69 to 9168828.03) | 34163.58 (31398.51 to 36944.87) | 13943449.73 (13210829.22 to 14756113.66) | 29931.25 (27330.32 to 32630.89) | -0.39 (-0.46 to -0.32) |
| Nauru | 1067.43 (909.86 to 1238.19) | 22300.6 (16500.41 to 28966.84) | 763.2 (629.34 to 925.48) | 19273.92 (13692.72 to 25917.22) | -0.4  (-0.52 to -0.28) |
| Cabo Verde | 54423.74 (48377.19 to 61259.71) | 34321.53 (27265.58 to 41267.75) | 48281.69 (40855.23 to 55583.06) | 30741.05 (22989.88 to 39376.9) | -0.42 (-0.45 to -0.39) |
| India | 138932562.03 (135177369.42 to 142692743.85) | 41603.11 (40069.72 to 43157.34) | 133648265.61 (130036808.64 to 137094113.08) | 36390.4 (35044.81 to 37675.62) | -0.42 (-0.46 to -0.38) |
| Georgia | 238689.64 (190096.84 to 289384.29) | 17604.56 (11964.35 to 23936.46) | 109841.49 (85447.16 to 135669.16) | 15165.39 (9887.06 to 21417.62) | -0.43 (-0.49 to -0.37) |
| American Samoa | 3308.13 (2759.03 to 3914.13) | 16257.3 (11972.46 to 21202.8) | 2182.72 (1736.91 to 2649.23) | 14142.94 (9951.3 to 19277.54) | -0.45 (-0.53 to -0.37) |
| Cambodia | 1684420.21 (1512584.34 to 1848807.72) | 33883.52 (27634.77 to 40175.04) | 1483606.79 (1281542.77 to 1682924.34) | 29423.94 (23048.66 to 36345.47) | -0.46 (-0.51 to -0.41) |
| Kyrgyzstan | 370313.76 (313949.26 to 426959.63) | 21698.48 (16278.45 to 27864.44) | 404416.91 (334650.69 to 478910.21) | 19017.09 (13703.83 to 25176.02) | -0.47 (-0.55 to -0.39) |
| Guyana | 83768.44 (72631.27 to 95698.67) | 29329.34 (22819.72 to 36168.06) | 50633.11 (42180.59 to 59729.28) | 24464.75 (17926.34 to 31462.07) | -0.49 (-0.53 to -0.44) |
| Kenya | 2501891.12 (2414326.65 to 2586134.67) | 21404.71 (20297.47 to 22493.93) | 3506017.08 (3326368.99 to 3667090.3) | 18615.65 (17474.21 to 19755.03) | -0.49 (-0.55 to -0.43) |
| Barbados | 12408.45 (10345.9 to 14660.39) | 19985.25 (14508.44 to 26149.55) | 8222.47 (6432.13 to 10288.17) | 17143.37 (11128.64 to 24838) | -0.52 (-0.56 to -0.48) |
| Bulgaria | 235213.55 (183995.05 to 298934.38) | 14278.96 (9393.58 to 20888.04) | 117664.35 (88838.05 to 148694.54) | 12363.03 (7688.52 to 18115.16) | -0.53 (-0.55 to -0.51) |
| Sudan | 2934725.28 (2642631.68 to 3246109.69) | 30703.94 (25108.12 to 36186.9) | 4083676.67 (3587077.25 to 4582872.39) | 25864.36 (20888.78 to 31189.14) | -0.53 (-0.58 to -0.48) |
| Timor-Leste | 107866.18 (73985.27 to 126557.96) | 29653.78 (18559.01 to 37711.87) | 137435.48 (119335.81 to 154706.84) | 27352.08 (21707.05 to 33579.15) | -0.53 (-0.63 to -0.43) |
| Uzbekistan | 2713651.14 (2405557.99 to 3032275.05) | 30603.09 (24668.73 to 37097.56) | 2741073.6 (2316644.02 to 3159076.96) | 26479.43 (20129.94 to 33703.22) | -0.54 (-0.61 to -0.48) |
| Grenada | 8021.54 (7236.9 to 8849.71) | 25223.39 (21043.24 to 29451.45) | 4478.36 (3560.56 to 5481.55) | 20991.29 (14567.44 to 28402.42) | -0.55 (-0.61 to -0.49) |
| Tuvalu | 879.08 (761.46 to 1004.8) | 25147.17 (18908.91 to 31629.28) | 683.2 (560.38 to 807.24) | 20895.4 (15145.49 to 27272.63) | -0.55 (-0.6 to -0.5) |
| Jamaica | 172738.27 (145621.4 to 203595.86) | 21124.71 (15712.19 to 27595.13) | 104858.29 (81211.89 to 130127.2) | 18200.04 (12361.6 to 25493.34) | -0.56 (-0.65 to -0.46) |
| New Zealand | 91210.27 (68813.41 to 117804.58) | 11493.45 (7645.43 to 16703.79) | 83720.75 (58400.86 to 115268.65) | 9762.95 (5502.18 to 15843.45) | -0.56 (-0.6 to -0.53) |
| Palau | 936.74 (759.81 to 1103.73) | 19878.62 (14237.2 to 26463.2) | 511.81 (403.08 to 633.22) | 16572.15 (11214.27 to 23192.83) | -0.56 (-0.63 to -0.49) |
| Afghanistan | 1143637.88 (994028.46 to 1300390.97) | 22003.3 (16997.95 to 27508.99) | 3401717.38 (2975310.57 to 3897104.78) | 19043.28 (14691.37 to 23753.96) | -0.57 (-0.68 to -0.47) |
| Saint Lucia | 11772.8 (9552.17 to 14150.02) | 22447.09 (15628.52 to 30216.52) | 5646.41 (4382.63 to 7058.5) | 18834.7 (12002.27 to 26874.57) | -0.58 (-0.66 to -0.51) |
| United Republic of Tanzania | 4681439.41 (4347305.48 to 5012933.42) | 37675.97 (32725.4 to 42380.93) | 8228933.5 (7315243.47 to 9201116.35) | 33029.57 (26415.04 to 39640.23) | -0.58 (-0.67 to -0.5) |
| Namibia | 176683.61 (136863.71 to 201217.43) | 29210.09 (21567.47 to 35743.61) | 209204.71 (173195.63 to 237236.09) | 24841.94 (18663.76 to 30812.36) | -0.59 (-0.7 to -0.48) |
| Liberia | 279556.01 (237127.35 to 318774.01) | 30166.2 (23882.32 to 36213.13) | 459118.9 (381129.99 to 542153.17) | 24850.69 (18645.68 to 31451.92) | -0.61 (-0.67 to -0.55) |
| Marshall Islands | 6385.03 (5873.77 to 6867.84) | 28499.71 (24789.84 to 32570.27) | 4330.72 (3699.74 to 4993.38) | 24057.52 (18629.83 to 30117.31) | -0.61 (-0.65 to -0.56) |
| Botswana | 166714.77 (148096.14 to 185592.89) | 29220.64 (23910.99 to 34996.44) | 169850.75 (140020.55 to 199456.37) | 24285.58 (17407.8 to 31896.67) | -0.62 (-0.69 to -0.55) |
| Comoros | 76396.03 (55676.39 to 85609.94) | 35327.72 (24834.01 to 42407.21) | 70915.79 (58294.66 to 82025.06) | 30772.32 (22803.07 to 38228.45) | -0.63 (-0.71 to -0.54) |
| Madagascar | 1905185.13 (1705202.24 to 2107358.62) | 33280.44 (27586.41 to 39468.75) | 3085972.09 (2687623.62 to 3450834.57) | 27995.42 (22401.88 to 33807.79) | -0.63 (-0.71 to -0.55) |
| Djibouti | 85461.33 (77558.26 to 93447.54) | 37731.03 (31523.85 to 44363.88) | 138074.84 (118556.76 to 156976.13) | 32142.24 (25042.38 to 39490.43) | -0.64 (-0.72 to -0.56) |
| Dominica | 3856.33 (3088.9 to 4697.68) | 15908.57 (10651.25 to 21894.19) | 1817.02 (1426.41 to 2273.33) | 13384.59 (8897.46 to 18956.92) | -0.65 (-0.76 to -0.53) |
| Eritrea | 548542.7 (497713.84 to 597017.92) | 38007.86 (31899.02 to 44013.91) | 853254.59 (752683.24 to 951377.38) | 32236.15 (25452.33 to 39014.73) | -0.65 (-0.67 to -0.62) |
| Trinidad and Tobago | 89032.52 (72246.87 to 107273.36) | 22261.87 (15369.25 to 29987.02) | 49594.37 (38205.85 to 62625.22) | 18979.3 (12130.65 to 26837.47) | -0.65 (-0.72 to -0.58) |
| United States Virgin Islands | 5950.16 (4662.03 to 7237.9) | 18908.44 (12537.36 to 26506.28) | 3091.63 (2350.3 to 3930.12) | 15947.7 (9631.1 to 23955.74) | -0.66 (-0.79 to -0.53) |
| Saint Kitts and Nevis | 2753.47 (2133.24 to 3389.11) | 19933.8 (13324.92 to 27645.53) | 1822.39 (1386.21 to 2302.35) | 16234.86 (9958.23 to 23711.74) | -0.67 (-0.76 to -0.59) |
| United Arab Emirates | 117777.71 (96150.51 to 142085.3) | 19091.64 (13607.96 to 25799.41) | 167169 (129159.92 to 209652.74) | 15206.52 (10002.09 to 21446.35) | -0.68 (-0.76 to -0.6) |
| Cook Islands | 1277.29 (1049.65 to 1534.39) | 18954.7 (13224.39 to 25253.71) | 608.11 (482.37 to 760.15) | 15258.6 (10137.4 to 21448.3) | -0.7 (-0.74 to -0.66) |
| Lithuania | 84444.33 (63632.1 to 109071.88) | 10229.07 (6469.03 to 15253.72) | 34856.3 (25742.17 to 46915.98) | 8437.34 (5234.08 to 13367.77) | -0.7 (-0.76 to -0.63) |
| Rwanda | 853261.52 (741729.05 to 961767.27) | 24343.34 (19593.67 to 29529.05) | 976406.51 (815691.48 to 1139003.84) | 20312.63 (15221.84 to 26051.86) | -0.7 (-0.83 to -0.57) |
| Uganda | 2503902.47 (2259765 to 2759563.29) | 28279.85 (23067.24 to 33615.16) | 4414265.06 (3837626.79 to 5045422.84) | 22757.28 (17511.88 to 28183.9) | -0.7 (-0.83 to -0.57) |
| Cuba | 444579.42 (335787.18 to 554687.48) | 17747.8 (11372.14 to 25411.13) | 255315.23 (190126.63 to 329541.7) | 14615.21 (8566.79 to 22133.17) | -0.71 (-0.76 to -0.65) |
| Honduras | 454946.25 (394876.89 to 519981.4) | 20184.33 (15345.09 to 25436.55) | 525370.98 (429627.09 to 632888.8) | 16082.51 (11486.7 to 21959.11) | -0.72 (-0.77 to -0.68) |
| Montenegro | 20069.92 (15583.34 to 25553.42) | 12770.84 (8156.23 to 18698.58) | 11396.69 (8579.32 to 14830.49) | 10908.82 (6842.19 to 16634.41) | -0.72 (-0.8 to -0.63) |
| Micronesia (Federated States of) | 12803.09 (11616.87 to 13981.92) | 26951.52 (22779.51 to 31375.3) | 6416.68 (5360.26 to 7443.95) | 21671.41 (16050.95 to 27680.07) | -0.73 (-0.79 to -0.68) |
| Paraguay | 286451.6 (233111.07 to 342552.37) | 16784.36 (11419.56 to 22793.16) | 250024.98 (189714.34 to 315171.37) | 13052.01 (8322.82 to 19129.54) | -0.75 (-0.81 to -0.69) |
| Venezuela (Bolivarian Republic of) | 867023.64 (698589.56 to 1068598.51) | 12049.83 (8195.01 to 16639.52) | 637698.63 (483577.09 to 816886.38) | 9402.65 (6164.12 to 13944.64) | -0.77 (-0.84 to -0.7) |
| Antigua and Barbuda | 4282.7 (3507.94 to 5059.78) | 22922 (16108.52 to 30164.36) | 2972.32 (2322.29 to 3672.39) | 18692.84 (11890.65 to 26310.19) | -0.8 (-0.85 to -0.74) |
| Libya | 367079.7 (304265.48 to 443142.37) | 19089.96 (13161.6 to 25743.14) | 212020.3 (165739.52 to 267066.88) | 15619.51 (10339.47 to 22177.84) | -0.81 (-0.92 to -0.7) |
| Egypt | 5375714.62 (4566795.45 to 6230716.34) | 23749.73 (17825.24 to 30279.19) | 5737500.13 (4623098.26 to 7004844.45) | 17654.24 (12376.77 to 23885.69) | -0.82 (-0.96 to -0.69) |
| Tokelau | 182.33 (158.15 to 208.91) | 24146.48 (17987.82 to 30867.97) | 92.03 (75.54 to 108.51) | 18954.55 (13366.97 to 25323.67) | -0.83 (-0.85 to -0.81) |
| United Kingdom | 948537.3 (773178.88 to 1158011.11) | 8661.23 (6192.28 to 11877.31) | 814056.19 (608230.83 to 1094058.3) | 7004.11 (4524.49 to 10822.87) | -0.83 (-0.89 to -0.77) |
| Tajikistan | 526200.75 (435306.38 to 600673.65) | 21526.53 (15845.14 to 27351.1) | 589873.05 (490575.88 to 689959.47) | 17735.46 (12996.6 to 23171.31) | -0.84 (-0.93 to -0.74) |
| Albania | 205816.4 (168136.57 to 244922.38) | 18277.8 (12572.98 to 24599.25) | 67271.88 (55767.99 to 82246.84) | 14335.53 (10397.05 to 19327.36) | -0.86 (-1.04 to -0.69) |
| Niue | 171.9 (141.55 to 206.06) | 20856.81 (15060.09 to 27470.3) | 62.67 (49.9 to 77.44) | 16467.88 (11112.81 to 22924.57) | -0.86 (-0.91 to -0.81) |
| Saudi Arabia | 859908.25 (755184.22 to 977075.62) | 12271.22 (9792.57 to 15017.94) | 616294.45 (466335.26 to 792808.84) | 9170.53 (6198.78 to 13513.66) | -0.86 (-0.93 to -0.79) |
| Romania | 910803.48 (761782.55 to 1085956.05) | 17077.31 (12997.65 to 21984.57) | 388851.12 (304171.06 to 481521.75) | 13637.04 (8977.91 to 19362.54) | -0.87 (-0.94 to -0.79) |
| Turkmenistan | 314800.8 (271137.59 to 360401.92) | 20110.39 (15507.93 to 25347.41) | 246969.71 (203726.81 to 296848.19) | 15953.19 (11300.5 to 21491.26) | -0.88 (-0.95 to -0.82) |
| Mauritius | 86148.06 (75590.95 to 97887.2) | 27422.3 (22075.51 to 33364.69) | 41035.62 (33918.34 to 48735.39) | 20979.73 (15638.04 to 27313.49) | -0.9 (-0.94 to -0.86) |
| Slovakia | 167983.7 (129972.37 to 214665.83) | 13348.09 (8393.55 to 19396.73) | 84806.08 (61296.23 to 112530.93) | 10156.56 (6180.38 to 15779.65) | -0.91 (-0.96 to -0.86) |
| Morocco | 2388596.78 (2041602.88 to 2759537.37) | 24156.71 (18335.11 to 30804.7) | 1681494.85 (1354027.87 to 2043400.1) | 18304.79 (12744.44 to 24925.53) | -0.92 (-0.96 to -0.88) |
| Algeria | 2317678.58 (1902237.24 to 2749253.88) | 21529.1 (15341.66 to 28296.62) | 1936785.17 (1530961.63 to 2415740.06) | 16145.66 (10891.63 to 23040.14) | -0.95 (-0.97 to -0.94) |
| Brunei Darussalam | 14932.22 (12125.89 to 18064.99) | 15962.39 (11079.8 to 21820.96) | 11324.21 (8578.57 to 14839.58) | 12148.9 (7564.18 to 18654.86) | -0.95 (-1.02 to -0.88) |
| Ukraine | 1073312.46 (851604.5 to 1324281.23) | 9745.46 (6681.66 to 13788.58) | 527096.45 (411586.39 to 668963.23) | 8113.58 (5655.03 to 11763.93) | -0.95 (-1.05 to -0.84) |
| Kuwait | 93763.23 (82103.37 to 105907.78) | 16286.48 (12793.81 to 20187.55) | 108230.38 (84222.8 to 135131.45) | 12552.52 (8319.5 to 17941.4) | -0.96 (-1.05 to -0.88) |
| Mexico | 4647297.59 (4458394.75 to 4848818.69) | 13889.04 (12946.44 to 14853.73) | 3198469.68 (3051026.11 to 3358126.52) | 10197.7 (9479.73 to 10947.33) | -0.96 (-1.11 to -0.81) |
| Brazil | 9354295.31 (7918090.08 to 11006935.52) | 18179.07 (13531.53 to 23636.73) | 6351889.68 (5191239.22 to 7699384.3) | 13631.76 (9562.83 to 18500.78) | -0.97 (-1.01 to -0.93) |
| Malaysia | 1432507.78 (1310407.43 to 1548733.14) | 21487.46 (18503.46 to 24509.16) | 1233907.29 (1017509.66 to 1480706.89) | 16372.95 (11896.27 to 21860.58) | -0.97 (-0.99 to -0.94) |
| Sweden | 99817.11 (66644.48 to 142192.58) | 6402.09 (3476.25 to 10955.09) | 85905.53 (55415.05 to 130706.25) | 4853.41 (2636.51 to 8936.21) | -0.97 (-1.06 to -0.89) |
| Palestine | 197643.95 (164399.29 to 233541.71) | 18786.25 (13373.88 to 24882.44) | 255471.31 (205007.7 to 304286.65) | 13897.53 (9808.24 to 18870.01) | -1 (-1.04 to -0.97) |
| Iraq | 1680786.53 (1427530.17 to 1969816.02) | 19685.17 (15136 to 25124.44) | 2077945.99 (1682830.59 to 2521671.72) | 15319.23 (10910.74 to 20836.42) | -1.01 (-1.1 to -0.92) |
| North Macedonia | 84277.77 (69869.2 to 99439.52) | 16037.45 (11682.75 to 20604.9) | 40849.81 (33539.16 to 49941.56) | 12094.78 (8534.46 to 16447.99) | -1.02 (-1.06 to -0.97) |
| Syrian Arab Republic | 1436664.39 (1214731.48 to 1672356.06) | 22958.86 (17152.02 to 29616.16) | 639915.93 (508383.59 to 793843.5) | 17538.19 (12121.11 to 23925.98) | -1.02 (-1.14 to -0.89) |
| Czechia | 254987.08 (191073.72 to 321369.35) | 12592.02 (8036.39 to 18468.02) | 151237.12 (111061.46 to 199748.38) | 9089.86 (5363.97 to 14166.14) | -1.03 (-1.17 to -0.89) |
| Seychelles | 4865.3 (4000.64 to 5809.46) | 20584.87 (14670.73 to 27532.75) | 3322.42 (2573.13 to 4180.12) | 15391.61 (10016.49 to 22172.58) | -1.04 (-1.09 to -1) |
| Latvia | 61733.68 (46406.37 to 77739.64) | 10705.75 (6906.11 to 15772.99) | 24847.68 (17871.24 to 33369.99) | 8288.99 (5075.75 to 13059.31) | -1.06 (-1.13 to -0.99) |
| Uruguay | 131323.55 (100940.7 to 163921.79) | 16487.49 (10697.63 to 23373.9) | 84618.03 (62026.31 to 111251.31) | 12535.81 (7927.65 to 18791.8) | -1.06 (-1.13 to -0.98) |
| Greece | 121566.36 (81393.99 to 177369.43) | 6630.45 (3498.29 to 12072.64) | 68570.92 (44240.68 to 101017.51) | 4971.79 (2512.81 to 9203.23) | -1.09 (-1.25 to -0.94) |
| Croatia | 97483.16 (73594.12 to 125380.84) | 10572.66 (6735.55 to 15611.52) | 44756.06 (32450.46 to 59841.66) | 7955.17 (4872.84 to 12525.64) | -1.1 (-1.17 to -1.04) |
| Hungary | 273318.89 (211248.3 to 345945.68) | 13946.57 (9189.26 to 19952.07) | 133741.72 (98114.23 to 176082.22) | 10113.73 (6100.19 to 15831.77) | -1.1 (-1.21 to -0.98) |
| Australia | 360800.66 (250179.06 to 495825.43) | 9803.33 (5555.34 to 16084.61) | 322532.85 (207232.76 to 473711.08) | 7257.31 (3909.23 to 12842.39) | -1.13 (-1.21 to -1.05) |
| Greenland | 1446.67 (1047.65 to 1970.17) | 9888.44 (5353.51 to 16124.39) | 807.27 (545.37 to 1151.99) | 6960.94 (3531.4 to 12127.09) | -1.13 (-1.18 to -1.08) |
| Puerto Rico | 153295.52 (117636.14 to 193698.07) | 15930.54 (9976.36 to 23313.45) | 55244.86 (39283.53 to 75135.29) | 12020.1 (6915.31 to 18853.11) | -1.13 (-1.22 to -1.03) |
| Lao People's Democratic Republic | 518644.31 (455158.54 to 580207.77) | 27642.71 (21563.65 to 34315.39) | 464035.35 (384756.02 to 545719.03) | 20511.69 (14829.55 to 27005.69) | -1.14 (-1.19 to -1.09) |
| Monaco | 195.86 (125.6 to 294.37) | 5671.52 (3015.88 to 10692.23) | 204.75 (131.46 to 319.63) | 4234.32 (2240.4 to 8143.54) | -1.14 (-1.23 to -1.06) |
| Kazakhstan | 1152368.35 (1021854.05 to 1288201.66) | 22103.9 (17885.69 to 26572.96) | 861153.22 (707573.03 to 1029770.16) | 16654.97 (11702.78 to 22452.52) | -1.16 (-1.23 to -1.1) |
| South Africa | 2368990.59 (1993056.23 to 2767326.42) | 17943.23 (13139.86 to 23056.91) | 1981145.64 (1627214.35 to 2397606.38) | 13242.7 (9419.08 to 17770.27) | -1.16 (-1.4 to -0.91) |
| Canada | 205212.23 (142833.83 to 277560.21) | 3612.8 (2067.03 to 6022.55) | 152388.2 (104578.89 to 214200.28) | 2579.34 (1358.16 to 4670.46) | -1.17 (-1.29 to -1.04) |
| Costa Rica | 144583.63 (118173.73 to 172363.94) | 12693.24 (8977.46 to 17305.58) | 93948.76 (68494.51 to 125843.08) | 9185.84 (5311.96 to 14340.39) | -1.19 (-1.26 to -1.11) |
| Russian Federation | 2802779.79 (2297771.79 to 3369752.39) | 8209.17 (5839.51 to 11223.46) | 1698857.36 (1267989.03 to 2243972.01) | 6370.8 (4159.28 to 9763.07) | -1.19 (-1.37 to -1.01) |
| Tunisia | 463702.65 (403701.77 to 526665.08) | 14384.13 (11344.13 to 17896.5) | 260557.66 (202094.53 to 327173.45) | 10186.58 (6845.65 to 14851.1) | -1.19 (-1.22 to -1.16) |
| Belarus | 246256.27 (180463.2 to 317889.86) | 10375.24 (6233.82 to 15869.27) | 122313.51 (87457.53 to 167738.56) | 7484.65 (4369.37 to 12162.03) | -1.21 (-1.38 to -1.05) |
| San Marino | 260.49 (166.84 to 384.74) | 6081.74 (3159.44 to 10828.82) | 225.35 (147.15 to 334.85) | 4479.92 (2362.79 to 8396.3) | -1.21 (-1.31 to -1.11) |
| Austria | 93489.36 (63054.82 to 136367.13) | 7050.74 (3799.61 to 12194.32) | 62215.54 (40890.98 to 91910.91) | 4886.57 (2569.29 to 9010.97) | -1.22 (-1.33 to -1.1) |
| Jordan | 307556.52 (254622.87 to 364874.92) | 18123.45 (13131.07 to 23959.08) | 470791.15 (382100.33 to 566397.65) | 13031.83 (9184.14 to 17852) | -1.22 (-1.26 to -1.18) |
| Serbia | 315343.5 (250273.62 to 389458.84) | 15669.02 (10580.45 to 21666.35) | 159903.63 (119132.94 to 208796.11) | 11492.61 (6964.63 to 17271.79) | -1.22 (-1.29 to -1.15) |
| Iceland | 3594.87 (2375.74 to 5311.54) | 5779.26 (3070.08 to 10527.73) | 2692.19 (1733.31 to 4181.14) | 4180.55 (2199.8 to 7890.75) | -1.23 (-1.38 to -1.08) |
| Poland | 1415321.47 (1116206.32 to 1759276.9) | 15476.9 (10296.17 to 21554.64) | 632057.73 (473788.19 to 823869.64) | 11042.63 (6796.9 to 16660.23) | -1.24 (-1.32 to -1.15) |
| Andorra | 553.82 (360.69 to 803.46) | 6384.23 (3319.5 to 11220.18) | 435.43 (284.55 to 666.41) | 4581.58 (2417.64 to 8654.23) | -1.25 (-1.4 to -1.1) |
| Japan | 2180405.8 (1600274.17 to 2868208.65) | 10043.33 (5924.43 to 15664.41) | 1094229.18 (781993.63 to 1514847.08) | 7327.74 (4123.21 to 11989.11) | -1.25 (-1.31 to -1.18) |
| Switzerland | 71165.1 (47345.86 to 105181.93) | 6221.39 (3333.56 to 11278.1) | 56988.12 (36973.61 to 85460.59) | 4393.51 (2321.13 to 8096.52) | -1.25 (-1.38 to -1.12) |
| Slovenia | 45393.75 (33998.89 to 59378.36) | 11771.98 (7364.01 to 17737.9) | 25956.67 (18778.43 to 34728.8) | 8596.98 (5105.03 to 13707.94) | -1.26 (-1.34 to -1.19) |
| Bermuda | 1949.42 (1498.64 to 2439.22) | 16185.17 (10109.91 to 23741.86) | 934.37 (667.57 to 1241.94) | 11363.82 (6599.41 to 17536.53) | -1.27 (-1.39 to -1.15) |
| Peru | 1986505.37 (1704058.02 to 2307854.45) | 23524.31 (17724.2 to 29700.92) | 1536563.78 (1227660.53 to 1861874.74) | 16975.64 (11721.1 to 23283.08) | -1.27 (-1.39 to -1.15) |
| Dominican Republic | 493762.15 (407541.83 to 589292.4) | 17638.94 (12239.46 to 23843.95) | 361578.09 (285807.7 to 445829.88) | 11759.69 (7646.86 to 17035.47) | -1.28 (-1.41 to -1.15) |
| Philippines | 4931648.93 (4178572.97 to 5705735.18) | 18949.76 (14489.36 to 24067.84) | 4960277.91 (4190316.48 to 5770004.59) | 13829.24 (10654.48 to 17654.11) | -1.31 (-1.39 to -1.22) |
| Azerbaijan | 491230.14 (422459.56 to 566699.64) | 19784.09 (14706.51 to 25277.44) | 317025.99 (262559.39 to 377032.56) | 13888.09 (10156.92 to 18436.45) | -1.33 (-1.48 to -1.17) |
| Italy | 598595.96 (437416.35 to 823895.66) | 6829.49 (4090.95 to 11221.09) | 373085.04 (266473.13 to 542102.97) | 4904.62 (2865.89 to 8496.29) | -1.33 (-1.43 to -1.22) |
| Democratic People's Republic of Korea | 1547130.71 (1361920.39 to 1751692.72) | 21899.07 (16840.96 to 27424.33) | 787788.14 (657605.25 to 938904.61) | 16327.73 (11893.59 to 21769.84) | -1.35 (-1.54 to -1.15) |
| Spain | 755093.08 (530248.19 to 1033260.09) | 10994.41 (6264.87 to 17332.82) | 472318.81 (314007.43 to 678394.98) | 7523.78 (3970.77 to 13238.86) | -1.37 (-1.48 to -1.26) |
| Bosnia and Herzegovina | 180388.43 (145128.13 to 217662.75) | 16738.09 (11409.54 to 22876.86) | 55451.42 (41609.65 to 71063.21) | 12193.71 (7426.61 to 18091.36) | -1.38 (-1.55 to -1.21) |
| Bangladesh | 17574443.07 (15898993.36 to 19251318.21) | 35187.64 (29556.21 to 41240.6) | 10098667.36 (8586979.97 to 11701959.63) | 24035.73 (18502.65 to 30593.86) | -1.41 (-1.46 to -1.36) |
| Argentina | 1929188.2 (1657334.32 to 2252917.85) | 19323.7 (14810.27 to 24666.9) | 1403087.94 (1096072.1 to 1749733.05) | 13697.89 (9025.46 to 19645.1) | -1.42 (-1.64 to -1.2) |
| Bahrain | 32838.28 (27054.96 to 39179.21) | 19322.98 (13665.72 to 25725.91) | 28821.81 (21963.98 to 36581.89) | 13025.62 (8225.27 to 19460.86) | -1.43 (-1.47 to -1.38) |
| Netherlands | 160183.33 (102327.67 to 243622.05) | 5958.08 (3181.86 to 10762.24) | 109531.22 (69680.29 to 175460.16) | 4194.16 (2255.01 to 8076.91) | -1.43 (-1.53 to -1.33) |
| Malta | 7075.88 (4769 to 10330.64) | 8362 (4422.02 to 14886.35) | 3480.76 (2280.98 to 5128.79) | 5554.01 (2982.28 to 10114.27) | -1.48 (-1.56 to -1.41) |
| Thailand | 2772936.01 (2521143.77 to 3020064.01) | 17236.69 (14811.87 to 19921.54) | 1092606.26 (837051.45 to 1422281.34) | 11412.12 (7642.89 to 16842.96) | -1.49 (-1.54 to -1.43) |
| Estonia | 36601.28 (27461.22 to 46681.47) | 10529.51 (6682.35 to 15753.84) | 15043.24 (11023.04 to 20301.49) | 7311.6 (4627.4 to 11707.57) | -1.52 (-1.61 to -1.44) |
| Israel | 150205.99 (103900.21 to 206398.53) | 9920.64 (5440.5 to 16252.5) | 174708.11 (116881.5 to 252250.72) | 6616.33 (3489.52 to 11611.35) | -1.52 (-1.61 to -1.43) |
| Norway | 60414.28 (40806.98 to 88612.61) | 7647.94 (4174.37 to 12967.36) | 46328 (30363.65 to 71599.19) | 5150.97 (2733.59 to 9847.12) | -1.53 (-1.65 to -1.41) |
| Belgium | 124550.11 (81654.94 to 180745.63) | 7093.9 (3701.03 to 12414.87) | 87219.58 (56718.72 to 132899.77) | 4680.23 (2465.51 to 8615.66) | -1.57 (-1.65 to -1.49) |
| France | 775376.12 (515800.72 to 1135705.96) | 6771.99 (3657.45 to 11911.19) | 502318.6 (321430.1 to 783352.85) | 4487.82 (2384.96 to 8697.14) | -1.6 (-1.7 to -1.5) |
| Republic of Moldova | 204146.63 (160383.74 to 249414.93) | 16607.45 (11319.71 to 22972.16) | 60209.28 (46653.66 to 75482.28) | 11032.19 (7216.15 to 15821.98) | -1.6 (-1.74 to -1.46) |
| Oman | 286699.46 (264344.34 to 308701.22) | 33083.16 (28625.26 to 37463.44) | 223038.76 (174759.72 to 275014.47) | 20592.69 (13754.3 to 28664.16) | -1.65 (-1.81 to -1.5) |
| Germany | 841622.79 (596363.35 to 1170398.09) | 6516.38 (3572.88 to 10754.68) | 450462.98 (303282.45 to 659374.61) | 3915.59 (2058.46 to 7162) | -1.66 (-1.95 to -1.36) |
| Iran (Islamic Republic of) | 4480442.4 (3735535.15 to 5229251.41) | 17039.16 (12597.22 to 21895.45) | 2119652.3 (1714041.56 to 2597607.96) | 10398.67 (7444.44 to 14394.34) | -1.67 (-1.73 to -1.61) |
| Taiwan (Province of China) | 523393.75 (395708.45 to 674114.45) | 10136.11 (6237.8 to 15060.83) | 183208.37 (127799.11 to 249547.47) | 6259.05 (3568 to 10184.84) | -1.7 (-1.88 to -1.52) |
| Finland | 77009.88 (48502.37 to 116010.32) | 8298.71 (4277.95 to 14982.84) | 42166.95 (25784.63 to 71124.28) | 5176.13 (2566.29 to 10801.91) | -1.74 (-1.86 to -1.61) |
| Mongolia | 190983.71 (164593.23 to 217760.13) | 20870.72 (16071.06 to 25677.66) | 144934.66 (117594.3 to 173102.14) | 13850.16 (9455.74 to 18905.84) | -1.77 (-1.92 to -1.62) |
| Denmark | 67634.09 (45496.7 to 97777.38) | 8002.27 (4225.46 to 14200.88) | 46966.94 (29745.65 to 71568.94) | 5094.98 (2562.9 to 9693.05) | -1.78 (-1.91 to -1.66) |
| Luxembourg | 4782.01 (3188.52 to 6776.09) | 7270.37 (3797.95 to 12835.3) | 4283.1 (2811.78 to 6586.77) | 4446.58 (2363.93 to 8328.98) | -1.81 (-1.97 to -1.65) |
| Singapore | 75560.2 (57603.79 to 98148.46) | 12091.85 (7524.36 to 18390.17) | 60201.91 (44297.5 to 82145.46) | 7465.58 (4590.47 to 12128.75) | -1.86 (-1.94 to -1.77) |
| Republic of Korea | 1418027.56 (1187475.95 to 1675135.94) | 12885.76 (9442.25 to 17038.26) | 478709.21 (348479.01 to 646752.18) | 7236.85 (4286.94 to 11639.64) | -1.91  (-2.08 to -1.74) |
| Turkey | 4628614.81 (3828120.43 to 5440780.88) | 21461.59 (15318.48 to 28207.18) | 1961058.7 (1479494.41 to 2500874.63) | 13105.53 (8377.95 to 19269.42) | -1.92 (-2.02 to -1.82) |
| Lebanon | 256938.31 (218103.65 to 296600.44) | 19961.99 (14745.88 to 25855.15) | 167716.6 (126081.37 to 215735.37) | 11801.14 (7357.04 to 18093.64) | -1.93 (-2 to -1.86) |
| Ireland | 63476.38 (43205.07 to 91959.86) | 7060.94 (3901.11 to 12120.7) | 41653.66 (27882.74 to 63837.21) | 4281.57 (2313.58 to 8056.61) | -1.94 (-2.05 to -1.82) |
| Portugal | 184724.89 (126712.57 to 261677.49) | 9645.75 (5236.58 to 16362.2) | 73490.33 (48344.07 to 108770.17) | 5641.3 (2915.82 to 10494.17) | -1.94 (-2.07 to -1.81) |
| Qatar | 21476.38 (17682.24 to 25778.63) | 16465.23 (11602.63 to 22193.73) | 41146.09 (30265.6 to 54707.29) | 9999.83 (6168.46 to 16096.04) | -1.99 (-2.12 to -1.87) |
| Sri Lanka | 1383482.34 (1233901.7 to 1556965.82) | 25297.72 (21000.77 to 30022.34) | 746920.38 (629225.7 to 889557.19) | 15436.63 (11777.65 to 20324.62) | -2.07 (-2.23 to -1.92) |
| Nicaragua | 225828.57 (180772.77 to 279099.26) | 12093.83 (8223.29 to 17059.66) | 135509.85 (99847.92 to 177609.34) | 6926.94 (4280.17 to 10849.44) | -2.11 (-2.17 to -2.04) |
| Maldives | 40576.7 (36858.02 to 44269.87) | 36841.59 (31121.87 to 43255.7) | 25340.4 (21187.1 to 29654.19) | 22195.55 (16431.53 to 29175.49) | -2.14 (-2.27 to -2.02) |
| Viet Nam | 5668890.64 (4917035.82 to 6419885.97) | 21555.46 (16960.18 to 26518.09) | 2519755.86 (2076877.9 to 3004223.32) | 12210.82 (9028.05 to 16395.04) | -2.2 (-2.28 to -2.13) |
| Equatorial Guinea | 91300.33 (82067.78 to 99955.35) | 44725.63 (37798.91 to 51305.83) | 145240.62 (117686.65 to 175084.13) | 26154.37 (18705.07 to 34000.65) | -2.24 (-2.52 to -1.97) |
| Colombia | 1683673.18 (1347524.16 to 2052845.76) | 14159.71 (9709.81 to 19586.09) | 820551.65 (606241.63 to 1065736.2) | 7508.55 (4865.51 to 11109.88) | -2.34 (-2.44 to -2.23) |
| Cyprus | 14608.71 (9961.92 to 20368.03) | 7571.28 (4197.22 to 12741.9) | 8527.44 (5714.71 to 12895.83) | 4004.27 (2197.86 to 7541.81) | -2.5 (-2.64 to -2.35) |
| Indonesia | 19030810.53 (17224194.83 to 21225575.54) | 28288.64 (23584.88 to 33198.23) | 9362498.83 (8080029.59 to 10665198.38) | 15089.56 (12027.66 to 18755.65) | -2.57 (-2.77 to -2.36) |
| Chile | 193995.25 (132586.28 to 283372.37) | 4790.99 (2537.95 to 8810.84) | 73981.99 (47814.61 to 116439.59) | 2112.67 (1096.34 to 4085.94) | -2.71 (-2.92 to -2.5) |
| China | 41469856.27 (38496403.8 to 44442439.42) | 12762.82 (11237.24 to 14376.81) | 12293062.28 (10929123.49 to 14092984.34) | 5397.33 (4405.29 to 6574.81) | -3.29 (-3.48 to -3.1) |
| Ecuador | 744560.09 (615173.97 to 889161.99) | 19033.34 (13588.81 to 25195) | 337330.97 (267302.05 to 416621.5) | 6788.37 (4609.82 to 9650.21) | -4.03 (-4.2 to -3.85) |

**Supplementary Table 5** DALY of nutritional deficiency among children in 1990 and 2019 and the EAPC from 1990 to 2019 in different countries and territories

| Location | 1990 |  | 2019 |  | 1990-2019 |
| --- | --- | --- | --- | --- | --- |
|  | DALY cases NO.(95%UI) | ASR/100,000 (95% CI) | DALY cases NO.(95%UI) | ASR/100,000 (95% CI) | EAPC (95%CI) |
| Afghanistan | 205956.61 (129272.42 to 332304.61) | 3688.32 (2188.19 to 6245.1) | 209321.55 (146261 to 293209.56) | 1152.17 (732.6 to 1770.88) | -3.99  (-4.33 to -3.65) |
| Albania | 9334.69 (6725.15 to 12836.66) | 827.23 (536.7 to 1256.14) | 1910.41 (1227.71 to 2966.72) | 406.78 (229.74 to 694.02) | -3.19  (-3.65 to -2.73) |
| Algeria | 110633.79 (72259.82 to 163245.65) | 1028.46 (587.29 to 1693.27) | 58168.37 (36488.98 to 91636.9) | 485.79 (263.91 to 861.32) | -2.65  (-2.71 to -2.58) |
| American Samoa | 184.98 (133.31 to 250.94) | 896.31 (589.89 to 1316.01) | 81.37 (55.57 to 118.35) | 527.13 (314.56 to 840.88) | -1.9  (-1.99 to -1.81) |
| Andorra | 11.67 (6.47 to 20.13) | 126.57 (55.79 to 255.55) | 9.57 (4.88 to 16.48) | 89.32 (38.87 to 186.52) | -1.37  (-1.52 to -1.22) |
| Angola | 941985.94 (487264.93 to 1725152.08) | 16879.23 (8495.49 to 31053.39) | 290641.87 (201002.77 to 393843.31) | 2002.69 (1282.77 to 2931.63) | -7.42  (-7.67 to -7.16) |
| Antigua and Barbuda | 177.57 (124.25 to 251.25) | 950.24 (585.59 to 1469.44) | 101.85 (66.88 to 149.27) | 641.43 (359.74 to 1058.7) | -1.42  (-1.5 to -1.34) |
| Argentina | 118241.92 (94797.33 to 152055.52) | 1191.51 (911.3 to 1602.41) | 37548.06 (24092.82 to 58217.18) | 365.74 (206.9 to 642.84) | -4.44  (-4.77 to -4.11) |
| Armenia | 6093.09 (3853.49 to 9054.58) | 578.06 (319.28 to 935.78) | 2762.97 (1649.18 to 4387.7) | 455.18 (236.35 to 788.33) | -0.75  (-0.81 to -0.7) |
| Australia | 6913.74 (3643.99 to 12853.18) | 186.74 (77.7 to 396.28) | 5375.09 (2627.17 to 9917.41) | 119.87 (47.55 to 264.56) | -1.64  (-1.75 to -1.52) |
| Austria | 1946.54 (1052.24 to 3473.14) | 144.55 (63.27 to 291.27) | 1269.16 (681.02 to 2149.93) | 98.97 (44.27 to 200.32) | -1.2  (-1.36 to -1.03) |
| Azerbaijan | 27041.38 (20382.55 to 35534.83) | 1073.95 (725.42 to 1532.82) | 8859.96 (5481.9 to 13283.24) | 385.15 (214.54 to 639.19) | -3.81  (-3.93 to -3.7) |
| Bahamas | 688.72 (474.16 to 991.93) | 886.67 (542.22 to 1368.98) | 442.03 (273.44 to 656.92) | 587.89 (320.11 to 1008.1) | -1.54  (-1.73 to -1.35) |
| Bahrain | 1047.11 (668.08 to 1626.83) | 618.09 (341.02 to 1043.44) | 675.22 (384.31 to 1113.43) | 300.4 (140.82 to 579.33) | -2.59  (-2.66 to -2.52) |
| Bangladesh | 5173054.36 (3919117.11 to 6460235.8) | 9994.99 (7103.49 to 13086.86) | 455333.37 (317706.06 to 639134.89) | 1087.65 (688.06 to 1633.89) | -7.67  (-7.91 to -7.43) |
| Barbados | 558.95 (396.75 to 789.6) | 899.07 (578.92 to 1341.04) | 276.96 (173.71 to 430.79) | 564.13 (308.78 to 959.92) | -1.6  (-1.74 to -1.47) |
| Belarus | 7440.67 (4892.22 to 11425.08) | 312.22 (178.37 to 546.58) | 2813.18 (1713.44 to 4535.92) | 171.36 (88.31 to 324.25) | -2.27  (-2.46 to -2.07) |
| Belgium | 2455.17 (1352.24 to 4231.78) | 137.9 (58.97 to 289.36) | 1665.68 (865.55 to 2916.45) | 86.47 (37.19 to 177.65) | -1.85  (-1.95 to -1.75) |
| Belize | 1705.26 (1367.79 to 2101.2) | 2091.86 (1550.03 to 2802.58) | 1001.57 (690 to 1409.93) | 859.97 (517.5 to 1329.52) | -2.98  (-3.23 to -2.73) |
| Benin | 230642.29 (147433.32 to 356412.86) | 8291.52 (5173.37 to 13099.4) | 122436.25 (84686.88 to 168561.12) | 2013.34 (1274.66 to 3041.87) | -4.92  (-5.24 to -4.59) |
| Bermuda | 55.98 (34.35 to 90.81) | 466.44 (236.08 to 856.89) | 20.75 (11.44 to 34.97) | 245.73 (107.34 to 473.67) | -2.26  (-2.47 to -2.06) |
| Bhutan | 12310.19 (6770.52 to 20486.75) | 4679.23 (2373.18 to 8517.6) | 4937.45 (3325.53 to 7184.02) | 2584.64 (1621.8 to 3834.12) | -2.25  (-2.34 to -2.15) |
| Bolivia (Plurinational State of) | 160631.83 (109036.31 to 227667.59) | 5432.53 (3497.11 to 7963.87) | 50872.36 (35678.49 to 70534.1) | 1226.12 (777.63 to 1827.2) | -5.12  (-5.22 to -5.02) |
| Bosnia and Herzegovina | 5704.92 (3370.49 to 9038.48) | 524.76 (264.29 to 917.93) | 1484.38 (861.59 to 2450.2) | 318.89 (147.07 to 601.17) | -2.19  (-2.44 to -1.93) |
| Botswana | 16835.65 (12221.17 to 23267.8) | 2981.27 (1949.68 to 4383.3) | 16496.35 (10963.69 to 23353.11) | 2399.57 (1437.69 to 3770.93) | 0.2  (-0.2 to 0.59) |
| Brazil | 1379813.26 (1172453.62 to 1621792.51) | 2792.51 (2296.26 to 3424.44) | 286800.54 (207587.84 to 396782.45) | 620.33 (408.19 to 923.05) | -5.54  (-5.75 to -5.33) |
| Brunei Darussalam | 413.19 (247.63 to 625.86) | 448.22 (232.9 to 765.19) | 268.94 (155.81 to 441.93) | 285.78 (129.5 to 545.69) | -1.59  (-1.71 to -1.46) |
| Bulgaria | 6880.67 (3953.72 to 10795.7) | 412.94 (205.04 to 748.69) | 3164.82 (1837.3 to 4922.2) | 328.37 (158.9 to 600.15) | -0.87  (-0.94 to -0.8) |
| Burkina Faso | 340971.65 (218132.35 to 505309.55) | 6527.76 (3991.95 to 10047.35) | 530960.3 (356810.49 to 754647.54) | 4759.19 (3014.88 to 7354.74) | -1.27  (-1.78 to -0.77) |
| Burundi | 311632.56 (188022.5 to 466900.23) | 10199.14 (6002.46 to 15609.36) | 170384.92 (106228.28 to 272080.71) | 2955.51 (1718.11 to 4946.25) | -4  (-4.35 to -3.65) |
| Cabo Verde | 5675.46 (4154.9 to 7614.03) | 3500.81 (2330.68 to 5074.42) | 1914.41 (1234.86 to 2717.12) | 1220.73 (704.6 to 1925.98) | -4.01  (-4.27 to -3.76) |
| Cambodia | 343727.96 (152119.75 to 558580.77) | 6535.77 (2851.41 to 10854.75) | 57152.25 (39173.46 to 81930.33) | 1133.36 (707.49 to 1744.91) | -6.28  (-6.58 to -5.97) |
| Cameroon | 161483.67 (107681.73 to 230857.29) | 3006.91 (1888.97 to 4627.17) | 168808.54 (112855.25 to 238377.47) | 1399.28 (856.12 to 2127.73) | -2.82  (-2.98 to -2.66) |
| Canada | 4519.62 (2523.52 to 7551.34) | 78.76 (34.98 to 158.39) | 3217.37 (1725.71 to 5360.89) | 52.87 (22.61 to 106.43) | -1.44  (-1.58 to -1.3) |
| Central African Republic | 78382.89 (48882.72 to 127143.53) | 5604.2 (3274.35 to 9420.57) | 105647.21 (69454.44 to 154887.53) | 4476.16 (2721.96 to 6867.17) | -0.25  (-0.6 to 0.11) |
| Chad | 491132.39 (305233.13 to 725702.71) | 14175.14 (8610.74 to 21137.89) | 318954.2 (227806.86 to 430528.07) | 3529.43 (2325.65 to 5100.69) | -5.06  (-5.26 to -4.85) |
| Chile | 5128.2 (3536.66 to 7889.88) | 126.9 (75.08 to 225.96) | 1682.81 (1071.9 to 2739.61) | 47.08 (25.28 to 89.36) | -3.18  (-3.34 to -3.02) |
| China | 3914602.53 (3241129.37 to 4650210.47) | 1192.05 (948.61 to 1469.53) | 342551.66 (241941.77 to 468917.76) | 150.97 (99.36 to 222.12) | -7.74  (-8.18 to -7.3) |
| Colombia | 145498.77 (119569.59 to 175677.9) | 1210.03 (945.36 to 1557.31) | 52289.56 (38750.68 to 68976.71) | 478.09 (323.61 to 696.35) | -2.85  (-3.21 to -2.49) |
| Comoros | 14920.71 (8070.36 to 22912.56) | 6521.94 (3257.26 to 10244.91) | 4609.44 (3200.69 to 6089.5) | 2016.3 (1267.33 to 2946.91) | -3.95  (-4.08 to -3.82) |
| Congo | 36879.1 (24065.69 to 54118.14) | 3140.9 (1828.84 to 4976.16) | 29185.33 (19068.61 to 41801.7) | 1462.94 (883.93 to 2245.59) | -2.87  (-3.15 to -2.58) |
| Cook Islands | 40.48 (24.88 to 61.43) | 601.54 (313.03 to 1028.25) | 15.71 (9.27 to 25.26) | 391.28 (189.56 to 718.12) | -1.43  (-1.48 to -1.38) |
| Costa Rica | 5446.93 (3813.7 to 7645.55) | 476.98 (307.44 to 736.2) | 2365.78 (1377.92 to 3825.27) | 229.14 (110.64 to 420.62) | -2.4  (-2.64 to -2.15) |
| Croatia | 2344.41 (1330.11 to 3784.53) | 249.89 (117.75 to 457.52) | 933.47 (539.85 to 1539.55) | 162.23 (75.39 to 308.15) | -1.67  (-1.77 to -1.57) |
| Cuba | 13395.52 (8051.96 to 21136.41) | 536.94 (271.52 to 968.53) | 6678.83 (3708.74 to 10931.74) | 375.06 (171.53 to 711.51) | -1.34  (-1.44 to -1.24) |
| Cyprus | 308.94 (169.36 to 527.23) | 157.13 (69.34 to 311.05) | 161.15 (88.94 to 286.26) | 75.71 (34.32 to 157.8) | -2.96  (-3.13 to -2.79) |
| Czechia | 6672.57 (3774.14 to 10747.37) | 323.44 (148.43 to 607.56) | 3248.91 (1856.74 to 5439.49) | 193.91 (82.88 to 381.93) | -1.62  (-1.83 to -1.41) |
| C么te d'Ivoire | 238419.89 (165289.59 to 337760.11) | NA | 192126.71 (126817.93 to 276134.99) | NA | NA |
| Democratic People's Republic of Korea | 176047.22 (96777.67 to 296992.92) | 2273.94 (1158.02 to 3997.75) | 24626.87 (16250.2 to 35773.73) | 510.53 (293.7 to 832.68) | -11.41  (-16.22 to -6.31) |
| Democratic Republic of the Congo | 1354419.19 (943871.75 to 1873387.41) | 6470.19 (4312.75 to 9312.93) | 804738.68 (549118.75 to 1105811.14) | 2100.14 (1327.81 to 3161.13) | -3.81  (-4.34 to -3.29) |
| Denmark | 1427.54 (763.77 to 2438.05) | 166.67 (72.58 to 344.38) | 900.96 (484.96 to 1610.45) | 94.94 (40.67 to 199.08) | -2.25  (-2.42 to -2.08) |
| Djibouti | 29641.29 (16298.96 to 47229.06) | 11744.89 (6171.13 to 18886.69) | 15574.68 (10215.76 to 22429.18) | 3502.75 (2150.77 to 5340.88) | -4.47  (-4.86 to -4.07) |
| Dominica | 185.85 (135.83 to 254.83) | 769.21 (507.25 to 1165.48) | 75.11 (51.14 to 106.62) | 561.35 (330.61 to 874.07) | -1.11  (-1.43 to -0.8) |
| Dominican Republic | 109088.38 (83712.7 to 139366.75) | 3717.76 (2722.74 to 4956.97) | 20892.47 (13859.52 to 29773.91) | 675.6 (401.93 to 1080.43) | -5.47  (-6.12 to -4.81) |
| Ecuador | 79196.83 (65766.3 to 95307.69) | 2004.67 (1557.47 to 2572.91) | 15028.22 (11163.53 to 19977.81) | 302.98 (203.11 to 454.68) | -7.19  (-7.71 to -6.67) |
| Egypt | 261015.65 (186927.16 to 362636.78) | 1138.4 (726.55 to 1711.93) | 171167.93 (103345.19 to 263308.37) | 524.22 (280.91 to 891.84) | -2.43  (-2.54 to -2.32) |
| El Salvador | 31343.23 (24625.56 to 38693.9) | 1476.21 (1074.1 to 2009.35) | 7728.71 (5059.08 to 11522.24) | 456.62 (258 to 748.49) | -3.64  (-3.79 to -3.49) |
| Equatorial Guinea | 17406.71 (11074.16 to 27609.21) | 7165.38 (4358 to 11648.17) | 5583.33 (3524.44 to 8516.09) | 1010.98 (551.1 to 1655.01) | -7.63  (-8.16 to -7.09) |
| Eritrea | 329772.43 (202491.12 to 504924.78) | 21028.65 (12610.49 to 32332.78) | 82779.7 (59280.28 to 114608.9) | 3116.53 (2008.27 to 4622.05) | -5.8  (-6.18 to -5.43) |
| Estonia | 1114.7 (755.26 to 1683.83) | 320.46 (188.68 to 543.62) | 307.25 (180.64 to 511.9) | 146.83 (72.45 to 282.22) | -2.91  (-3.03 to -2.78) |
| Eswatini | 16005.17 (10303.2 to 22469.93) | 3905.52 (2304.71 to 5758.57) | 8250.07 (5414.99 to 11972.98) | 2038.01 (1217.22 to 3186.06) | -1.7  (-1.98 to -1.42) |
| Ethiopia | 3168927.81 (2244205.9 to 4433173.73) | 11457.73 (7935.03 to 16176.85) | 1016582.52 (755011.03 to 1321302.19) | 2160.05 (1535.67 to 2919.23) | -6.24  (-6.53 to -5.94) |
| Fiji | 3236.02 (2347.69 to 4412.13) | 1161.43 (773.46 to 1665.05) | 2714.57 (1864.9 to 3815.86) | 1035.52 (634.2 to 1589.96) | -0.38  (-0.51 to -0.24) |
| Finland | 1388.19 (748.35 to 2423.39) | 146.12 (64.25 to 292.9) | 799.81 (412.7 to 1373.76) | 91.71 (39.88 to 194.9) | -1.85  (-1.99 to -1.71) |
| France | 15444.59 (8187.35 to 26504.3) | 133.08 (57.55 to 279.63) | 9761.38 (5309.37 to 16866.47) | 83.21 (36.7 to 183.82) | -1.89  (-2 to -1.78) |
| Gabon | 12292.88 (8372.11 to 17491.29) | 2731.67 (1658.31 to 4167.84) | 7518.3 (4884.48 to 10773.28) | 1294.07 (755.17 to 1978.43) | -2.3  (-2.55 to -2.05) |
| Gambia | 31548.25 (22728.37 to 42115.23) | 5993.97 (4052.56 to 8546.69) | 24304.98 (16942.51 to 33532.92) | 2614.99 (1704.6 to 3804.21) | -3.27  (-3.45 to -3.09) |
| Georgia | 7957.73 (4851.82 to 12375.14) | 585.68 (305.65 to 999.62) | 3305.13 (1893.58 to 5293.15) | 454.61 (218.91 to 803.96) | -0.72  (-0.83 to -0.61) |
| Germany | 19305.98 (11067.74 to 31752.4) | 149.19 (73.19 to 276.17) | 10294.4 (5878.4 to 16904.82) | 88.38 (42.66 to 169.95) | -1.8  (-2.1 to -1.5) |
| Ghana | 380248.95 (245709.73 to 587614.6) | 5103.35 (3169.63 to 8028.08) | 227415.86 (154937.3 to 313718.51) | 2005.67 (1242.83 to 3041.96) | -3.11  (-3.37 to -2.86) |
| Greece | 2473.29 (1244.91 to 4504.17) | 127.1 (53.28 to 277.31) | 1311.99 (672.56 to 2421.52) | 91.53 (37.59 to 203.54) | -1.29  (-1.49 to -1.1) |
| Greenland | 36.27 (20.8 to 58.69) | 252.27 (109.66 to 499.63) | 17.85 (9.36 to 31.59) | 153.41 (61.34 to 323.64) | -1.61  (-1.68 to -1.54) |
| Grenada | 431.17 (321.29 to 570.8) | 1367.66 (966.87 to 1885.82) | 160.56 (103.02 to 237.27) | 752.33 (433.99 to 1236.83) | -1.84  (-2.02 to -1.66) |
| Guam | 286.22 (183.55 to 422.46) | 654.51 (378.52 to 1074.77) | 249.13 (156 to 378.48) | 547.8 (303.17 to 921.32) | -0.41  (-0.52 to -0.31) |
| Guatemala | 174887.57 (153564.89 to 199813.48) | 4418.35 (3743.95 to 5186.12) | 55109.74 (40765.58 to 72553.89) | 956.75 (646.46 to 1391.4) | -4.73  (-5.08 to -4.39) |
| Guinea | 351375.15 (230286.32 to 515141.71) | 10288.2 (6612.03 to 15394.04) | 204861.93 (135307.05 to 295704.91) | 3402.97 (2126.06 to 5190.02) | -3.76  (-3.89 to -3.63) |
| Guinea-Bissau | 47739.72 (28455.27 to 75130.28) | 9025.1 (5191.44 to 14571.67) | 17565.93 (12071.38 to 24304.62) | 2176.61 (1375.56 to 3239.2) | -5  (-5.23 to -4.78) |
| Guyana | 10548.8 (8552.02 to 12786.79) | 3630.11 (2753.83 to 4709.94) | 2592.78 (1856.31 to 3559.6) | 1257.06 (805.65 to 1894.94) | -2.83  (-3.15 to -2.51) |
| Haiti | 235947.61 (156431.8 to 355128.48) | 8053.79 (5093.81 to 12428.5) | 117215.78 (83724.97 to 161172.14) | 2711.06 (1741.52 to 4063.19) | -3.3  (-3.68 to -2.93) |
| Honduras | 37837.11 (28570.15 to 49412.08) | 1651.37 (1122.55 to 2333.33) | 16871.15 (10697.48 to 24841.93) | 516.26 (289.74 to 841.9) | -4.01  (-4.23 to -3.8) |
| Hungary | 7860.62 (4586.54 to 12448.7) | 394.74 (192.98 to 704.06) | 3228.01 (1790.71 to 5481.79) | 240.85 (108.29 to 472.92) | -1.67  (-1.84 to -1.51) |
| Iceland | 70.62 (37.01 to 123.79) | 111.81 (47.77 to 233.79) | 53.15 (27.54 to 93.67) | 79.09 (34.42 to 167.77) | -1.4  (-1.56 to -1.25) |
| India | 25170589.81 (16666148.24 to 35186813.01) | 7387.09 (4631.84 to 10473.26) | 7062469.46 (5007570.93 to 9558609.38) | 1941.76 (1364.9 to 2653.65) | -4.58  (-4.75 to -4.41) |
| Indonesia | 1778910.95 (1362155.57 to 2342005.92) | 2684.29 (1975.88 to 3704.32) | 429652.93 (312268.77 to 574203.16) | 700.44 (483.46 to 979.52) | -4.7  (-4.81 to -4.59) |
| Iran (Islamic Republic of) | 239257.57 (178664.54 to 316115.69) | 910.53 (628.45 to 1311.58) | 53581.24 (32511.72 to 82836.17) | 262.51 (144.88 to 446.81) | -4.02  (-4.19 to -3.85) |
| Iraq | 94338.45 (66840.38 to 129364.71) | 1088.22 (715 to 1617.84) | 63424.4 (39489.24 to 97785.79) | 467.14 (265.7 to 774.24) | -3.27  (-3.41 to -3.12) |
| Ireland | 1305.92 (693.6 to 2351.43) | 138.03 (59.86 to 283.79) | 852.95 (473.99 to 1464.4) | 83.28 (38.43 to 166.09) | -2.02  (-2.18 to -1.87) |
| Israel | 3558.15 (1862.57 to 6344) | 233.12 (101.27 to 466.67) | 3593.52 (1939.86 to 6355.21) | 138 (58.97 to 280.06) | -2.02  (-2.15 to -1.9) |
| Italy | 13883.13 (7900.28 to 21510.67) | 152.08 (75.92 to 278.15) | 8535.98 (4836.02 to 13952.3) | 106.09 (51.34 to 200.2) | -1.59  (-1.72 to -1.46) |
| Jamaica | 14650.28 (11611.78 to 18329.82) | 1818.55 (1359.56 to 2417.37) | 3335.37 (2116.87 to 5006.75) | 578.35 (315.86 to 968.35) | -3.47  (-4.16 to -2.77) |
| Japan | 53049.26 (29235.98 to 85830.95) | 235.37 (107.03 to 447.99) | 23008.4 (12424.29 to 39027.84) | 149.25 (64.32 to 294.22) | -1.82  (-1.92 to -1.72) |
| Jordan | 10613.61 (6788.13 to 15960.75) | 628.13 (348.78 to 1025.32) | 12644.84 (7833.93 to 19756.85) | 346.92 (181.08 to 612.64) | -2.19  (-2.26 to -2.12) |
| Kazakhstan | 44643.43 (30017.43 to 64600.53) | 856.34 (528.7 to 1309.13) | 27750.24 (17245.38 to 42517.04) | 537.63 (290.61 to 921.29) | -1.89  (-2.04 to -1.75) |
| Kenya | 590186.45 (447432.46 to 820564.41) | 4814.59 (3572.11 to 6771.2) | 326689.81 (249640.26 to 410487.66) | 1754.27 (1313.3 to 2269.28) | -3.17  (-3.46 to -2.88) |
| Kiribati | 2000.69 (1436.82 to 2732.26) | 6208.49 (4312.97 to 8681.44) | 948.47 (653.97 to 1298.28) | 2260.03 (1466.42 to 3318.18) | -3.55  (-3.7 to -3.39) |
| Kuwait | 2748.37 (1739.2 to 4043.3) | 482.16 (278.45 to 752.3) | 2709.97 (1612.38 to 4365.31) | 313.68 (156.17 to 578.13) | -1.51  (-1.61 to -1.42) |
| Kyrgyzstan | 14013.98 (8942.11 to 20269.98) | 822.04 (472.69 to 1304.51) | 12276.55 (7514.24 to 18883.08) | 577.85 (305.76 to 983.19) | -1.34  (-1.46 to -1.23) |
| Lao People's Democratic Republic | 110343.35 (55829.01 to 195781.16) | 5656.62 (2785.57 to 10230.82) | 19638.6 (13321.53 to 27978.42) | 866.87 (516.76 to 1353.86) | -6.2  (-6.35 to -6.06) |
| Latvia | 1848.39 (1199.54 to 2813.28) | 321.03 (187.58 to 544.54) | 538.7 (315.34 to 877.42) | 179.16 (89.17 to 332.17) | -2.08  (-2.2 to -1.96) |
| Lebanon | 7300.05 (4680.2 to 10732.53) | 568.83 (325.02 to 925.86) | 3582 (2117.73 to 5873.75) | 253.04 (128.69 to 472.8) | -2.88  (-2.97 to -2.8) |
| Lesotho | 33793.92 (23091.95 to 48688.73) | 4381.57 (2823.78 to 6575.75) | 16248.35 (11594.96 to 22103.97) | 2550.62 (1679.23 to 3751.15) | -1.08  (-1.55 to -0.6) |
| Liberia | 101563.93 (67058.3 to 148350.53) | 10755.98 (6903.39 to 16036.87) | 28274.99 (18727.09 to 40752.88) | 1538.82 (901.97 to 2400.33) | -6.93  (-7.29 to -6.57) |
| Libya | 12558.03 (7923.94 to 19251.36) | 653.81 (360.93 to 1082.36) | 5880.12 (3512.57 to 9714.68) | 426.53 (219.63 to 774.83) | -1.59  (-1.77 to -1.41) |
| Lithuania | 2335.38 (1515.84 to 3645.34) | 282.66 (159.63 to 491.93) | 744.98 (415.24 to 1204.13) | 179.77 (85.77 to 337.72) | -1.39  (-1.55 to -1.23) |
| Luxembourg | 95.06 (51.25 to 160.87) | 144.31 (61.78 to 296.41) | 81.46 (43.77 to 143.45) | 82.6 (35.75 to 174.8) | -2.1  (-2.3 to -1.91) |
| Madagascar | 948746.84 (764260.04 to 1139523.77) | 15597.22 (12314.89 to 19084.14) | 392847.7 (275831.91 to 539470.46) | 3522.13 (2284.25 to 5126.76) | -4.87  (-5.02 to -4.71) |
| Malawi | 507295.64 (344988.58 to 723449.14) | 10094.69 (6656.95 to 14656.77) | 208426.61 (149161.85 to 278929.11) | 2726.94 (1830.46 to 3906.44) | -4.91  (-5.22 to -4.6) |
| Malaysia | 57296.48 (38442.62 to 80981.05) | 860.25 (558.65 to 1254.66) | 39173.69 (24141.99 to 58276.11) | 518.33 (285.42 to 853.8) | -1.78  (-1.85 to -1.72) |
| Maldives | 4019.01 (2420.22 to 6645.83) | 3527.42 (1995.28 to 6074.88) | 842.39 (550.74 to 1198.15) | 739.07 (442.04 to 1156.55) | -5.33  (-5.77 to -4.89) |
| Mali | 699395.98 (455587.98 to 1003858.73) | 14529.43 (9155.9 to 20961.7) | 1376418.03 (975298.56 to 1918195.68) | 12124.41 (8258.07 to 17515.91) | -1.35  (-1.9 to -0.81) |
| Malta | 150.8 (81.49 to 262.44) | 174.9 (76.13 to 369.3) | 68.62 (36.7 to 117.1) | 109.2 (48.17 to 223.03) | -1.8  (-1.89 to -1.71) |
| Marshall Islands | 391.39 (287.24 to 527.49) | 1755.15 (1204.77 to 2448.65) | 200.31 (136.52 to 284.69) | 1116.18 (703.11 to 1684.78) | -1.69  (-1.89 to -1.48) |
| Mauritania | 73014.86 (38539.21 to 120474.31) | 6972.6 (3530.69 to 11755.45) | 29823.67 (18153.11 to 45157.9) | 1883.44 (1057.66 to 3148.39) | -4.73  (-5.12 to -4.34) |
| Mauritius | 3641.89 (2486.15 to 5290.23) | 1165.5 (729.73 to 1775.07) | 1391.91 (882.62 to 2092.54) | 714.9 (407.99 to 1174.94) | -1.49  (-1.61 to -1.36) |
| Mexico | 624381.3 (520454.53 to 740134.15) | 1860.48 (1504.44 to 2240.24) | 143072.35 (111006.17 to 183573.23) | 458.64 (344.93 to 604.54) | -5.01  (-5.28 to -4.74) |
| Micronesia (Federated States of) | 890.78 (637.5 to 1195.72) | 1868.65 (1266.17 to 2669.61) | 261.87 (174.91 to 375.62) | 886.88 (518.76 to 1354.35) | -2.68  (-2.86 to -2.5) |
| Monaco | 3.86 (2.06 to 7.04) | 109.94 (47.3 to 237.45) | 4.05 (2.15 to 7.02) | 81.65 (35.84 to 168.1) | -1.28  (-1.37 to -1.19) |
| Mongolia | 9503.92 (6605.01 to 13289.55) | 1032.75 (647.21 to 1545.77) | 4531.37 (2683.28 to 6822.82) | 441.67 (227.39 to 747.27) | -3.45  (-3.67 to -3.23) |
| Montenegro | 571.1 (340.39 to 907.84) | 360.8 (178.51 to 657.06) | 294.47 (170.78 to 465.18) | 278.04 (137.89 to 515.15) | -1.18  (-1.31 to -1.05) |
| Morocco | 112913.22 (79629.43 to 157616.1) | 1136.35 (731.7 to 1721.34) | 53980.77 (33394.98 to 83532.81) | 585.49 (313.29 to 999.74) | -2.21  (-2.25 to -2.16) |
| Mozambique | 824786.06 (525698.34 to 1308644.94) | 12506.07 (7718.32 to 20301.66) | 373555.29 (266068.36 to 506311.61) | 2609.94 (1737.19 to 3862.12) | -5.8  (-6.01 to -5.59) |
| Myanmar | 479695.73 (301520.83 to 800415.85) | 2954.92 (1703.14 to 5261.61) | 173445.64 (113674.05 to 257448.49) | 1180.6 (713.5 to 1842.48) | -3.25  (-3.35 to -3.16) |
| Namibia | 28976.44 (18477.24 to 40641.66) | 4646.42 (2776.09 to 6815.98) | 16834.6 (11433.56 to 23287.83) | 1989.61 (1231.22 to 2986.77) | -1.98  (-2.44 to -1.51) |
| Nauru | 81 (55.17 to 116.43) | 1620.43 (1019.85 to 2508.81) | 40.09 (27.16 to 57.63) | 1008.11 (616.76 to 1576.04) | -1.73  (-2.5 to -0.94) |
| Nepal | 684850.76 (443642 to 1049940.29) | 7217.75 (4497.9 to 11275.62) | 110204.7 (74637.88 to 156119.89) | 1234.61 (765.95 to 1857.69) | -5.79  (-6.07 to -5.51) |
| Netherlands | 2719.26 (1447.54 to 4800.85) | 100.77 (44.78 to 207.13) | 1931.9 (1053.72 to 3307.38) | 71.35 (31.14 to 146.01) | -1.53  (-1.65 to -1.4) |
| New Zealand | 1946.38 (1091.18 to 3354.76) | 246.02 (116.16 to 467.1) | 1620.68 (844.29 to 2908.36) | 187.38 (76.58 to 411.41) | -0.94  (-0.98 to -0.9) |
| Nicaragua | 47592.47 (36325.56 to 62754.85) | 2485.14 (1797.4 to 3406.87) | 9330.62 (6899.6 to 12406.85) | 484.14 (323.28 to 722.53) | -5.89  (-6.15 to -5.64) |
| Niger | 529500.47 (333592.93 to 849394.93) | 11279.03 (6762.78 to 18379.84) | 417490.56 (292581.71 to 573991.01) | 3208.92 (2057.86 to 4786.9) | -4.78  (-5.05 to -4.51) |
| Nigeria | 1576686.46 (1179225.56 to 2175637.96) | 3662.79 (2582.17 to 5356.79) | 2045299.7 (1478886.77 to 2832834.45) | 2163.57 (1464.46 to 3087.34) | -2.01  (-2.15 to -1.87) |
| Niue | 9.03 (6.09 to 12.76) | 1105.07 (681.78 to 1716.74) | 2.68 (1.78 to 3.91) | 711.23 (412.66 to 1157.99) | -1.82  (-2.03 to -1.61) |
| North Macedonia | 3076 (1973.78 to 4713.75) | 582.28 (331.83 to 966.15) | 1231.76 (740.56 to 1891.87) | 361.37 (186.33 to 627.37) | -1.69  (-1.79 to -1.6) |
| Northern Mariana Islands | 92.62 (61.35 to 133.81) | 697.94 (409.06 to 1111.68) | 34.96 (22.16 to 53.02) | 512.89 (288.43 to 860.84) | -0.64  (-0.84 to -0.45) |
| Norway | 1264.85 (693.29 to 2165.24) | 160.1 (73.82 to 315.89) | 995.04 (541.55 to 1812.46) | 106.01 (48.08 to 226.89) | -1.8  (-1.94 to -1.65) |
| Oman | 14097.63 (10125.97 to 19580.01) | 1614.92 (1102.49 to 2287.36) | 6361.6 (3785.19 to 9766.62) | 587.89 (313.94 to 1008.41) | -3.27  (-3.64 to -2.9) |
| Pakistan | 1895068.74 (1365576.48 to 2566828.73) | 3477.59 (2292.5 to 5060.86) | 1967335.25 (1447921.78 to 2618762.91) | 2254.66 (1539.26 to 3211.89) | -1.35  (-1.52 to -1.18) |
| Palau | 34.73 (22.59 to 50) | 737.11 (417.06 to 1189.5) | 15.26 (9.34 to 23.57) | 488.76 (257.62 to 861.12) | -1.24  (-1.36 to -1.12) |
| Palestine | 7138.19 (4680.47 to 10391.39) | 680.37 (390.96 to 1090.36) | 6715.06 (4119.17 to 10107.17) | 363.25 (192.27 to 629.29) | -2.04  (-2.11 to -1.97) |
| Panama | 9729.64 (7758.15 to 11829.83) | 1177.22 (874.48 to 1572.31) | 7254.84 (5408.45 to 9501.76) | 647.53 (437.08 to 939.65) | -3.03  (-3.77 to -2.3) |
| Papua New Guinea | 27266.31 (19457.47 to 36750.95) | 1557.6 (1026.33 to 2239.34) | 46815.92 (31956.43 to 64196.67) | 1209.56 (767.16 to 1805.17) | -0.63  (-0.77 to -0.5) |
| Paraguay | 17290.43 (12938.24 to 23172.33) | 1000.97 (670.46 to 1456.61) | 13257.77 (9065.34 to 18033.1) | 702.1 (420.76 to 1099.28) | -0.58  (-1.07 to -0.08) |
| Peru | 271848.74 (214902.99 to 342869.9) | 3175.97 (2359.19 to 4293.08) | 54416.71 (35352.86 to 78589.28) | 601.01 (342.8 to 968.45) | -6.25  (-6.46 to -6.04) |
| Philippines | 471870.7 (369047.54 to 612380) | 1784.42 (1328.44 to 2455.87) | 203208.77 (152377.82 to 280173.59) | 566.25 (394.63 to 820.73) | -3.83  (-4.01 to -3.65) |
| Poland | 44065.07 (25926.91 to 69528.34) | 473.33 (236.42 to 831.1) | 16137.17 (8901.12 to 26389.98) | 278.31 (130.21 to 523.93) | -1.96  (-2.08 to -1.84) |
| Portugal | 4963.38 (2905.24 to 8369.12) | 252.73 (128.26 to 492.48) | 1613.59 (894.36 to 2901.14) | 118.07 (54.07 to 257.17) | -2.72  (-2.96 to -2.48) |
| Puerto Rico | 4094.03 (2297.04 to 6555.93) | 420.84 (198.03 to 775.94) | 1291.27 (705.3 to 2161.23) | 266.73 (116.05 to 519.13) | -1.84  (-1.98 to -1.71) |
| Qatar | 561.53 (358.27 to 864.22) | 437.86 (243.87 to 736.43) | 815.37 (451.58 to 1397.5) | 197.08 (91.29 to 401.62) | -3.11  (-3.28 to -2.94) |
| Republic of Korea | 46262.75 (28573.39 to 70785.78) | 414.62 (228.44 to 683.95) | 12070.93 (6723.39 to 19951.91) | 180.44 (78.86 to 350.92) | -2.75  (-3 to -2.5) |
| Republic of Moldova | 5787.61 (3681.24 to 8925.36) | 470.28 (250.79 to 832.11) | 1417.08 (799.05 to 2354.35) | 251.23 (118.68 to 466.47) | -2.36  (-2.48 to -2.23) |
| Romania | 27945.98 (17127.93 to 42263.1) | 522.27 (288.79 to 872.12) | 10126.91 (5920.61 to 15901.81) | 351.56 (175.98 to 634.98) | -1.48  (-1.57 to -1.39) |
| Russian Federation | 69437.13 (43886.43 to 102114.59) | 201.44 (113.34 to 338.82) | 36474.24 (21859.74 to 57496.59) | 135.36 (69.29 to 243.74) | -1.91  (-2.17 to -1.64) |
| Rwanda | 274265.11 (184484.41 to 397453.6) | 7231.34 (4636.65 to 10880.82) | 88639.52 (59642.4 to 122657.39) | 1858.64 (1116.23 to 2889.02) | -5.58  (-6.18 to -4.98) |
| Saint Kitts and Nevis | 237.35 (189.01 to 293.63) | 1748.69 (1285.09 to 2346.86) | 75.47 (50.71 to 105.5) | 679.37 (373.38 to 1079.95) | -2.94  (-3.26 to -2.62) |
| Saint Lucia | 666.8 (495.18 to 911.22) | 1268.34 (852.12 to 1870.07) | 195.12 (125.4 to 298.56) | 649.38 (358.29 to 1103.65) | -2.24  (-2.47 to -2) |
| Saint Vincent and the Grenadines | 743.83 (574.16 to 946.88) | 1878.89 (1326.4 to 2612.47) | 211.06 (142.74 to 304.58) | 885.14 (521.66 to 1397.35) | -2.46  (-2.69 to -2.22) |
| Samoa | 651.57 (443.78 to 929.28) | 1040.18 (633.52 to 1599.07) | 449.44 (277 to 685.31) | 645.27 (353.81 to 1084.76) | -1.33  (-1.5 to -1.15) |
| San Marino | 5.24 (2.77 to 9.12) | 117.97 (48.6 to 253.04) | 4.47 (2.52 to 7.45) | 84.42 (36.63 to 172.47) | -1.41  (-1.51 to -1.31) |
| Sao Tome and Principe | 3374.59 (2348.7 to 4549.91) | 5725.9 (3661.04 to 8226.76) | 1003.99 (649.8 to 1423.27) | 1419.96 (818.14 to 2242.74) | -4.91  (-5.74 to -4.09) |
| Saudi Arabia | 36485.73 (25499.95 to 49972.53) | 519.42 (338.46 to 761.87) | 14001.6 (8265.66 to 22804.07) | 206.07 (104.51 to 373.33) | -2.94  (-3.09 to -2.79) |
| Senegal | 202838.08 (144272.76 to 282965.78) | 5119.27 (3343.19 to 7538.82) | 125808.45 (86194.99 to 178404.79) | 2056.73 (1313.15 to 3080.18) | -3.26  (-3.4 to -3.12) |
| Serbia | 10153.48 (6124.74 to 15615.21) | 500.96 (265.15 to 867.57) | 4079.55 (2316.15 to 6828.19) | 288.04 (135.2 to 552.01) | -2.12  (-2.23 to -2) |
| Seychelles | 157.78 (100.14 to 241.05) | 667.1 (373.15 to 1110.79) | 84.02 (49.26 to 131.58) | 388.4 (193.51 to 707.94) | -1.87  (-1.95 to -1.79) |
| Sierra Leone | 209264.5 (136177.39 to 312908.72) | 11520.27 (7220.9 to 17662.92) | 144243.94 (94446.3 to 211968.55) | 4171.82 (2495.24 to 6514.97) | -3.79  (-4.06 to -3.51) |
| Singapore | 1856.7 (1029.92 to 3094.73) | 291.91 (128.12 to 547.6) | 1076.4 (596.54 to 1788.54) | 135.35 (57.61 to 279.5) | -2.94  (-3.08 to -2.8) |
| Slovakia | 4726.79 (2741.9 to 7739.87) | 370.28 (178.95 to 681.39) | 2014.63 (1108.94 to 3354.49) | 239.77 (111.18 to 457.59) | -1.47  (-1.54 to -1.39) |
| Slovenia | 1192.12 (675.06 to 1941.04) | 302.63 (142.15 to 579.84) | 574.99 (319.3 to 966.65) | 187.37 (86.19 to 366.42) | -1.92  (-2.03 to -1.81) |
| Solomon Islands | 3877.03 (2670.94 to 5454.65) | 2307.77 (1461.88 to 3471.39) | 3385.68 (2418.91 to 4646.58) | 1273.62 (827.09 to 1899.47) | -2  (-2.14 to -1.87) |
| Somalia | 610777.23 (363234.62 to 966623.82) | 16596.85 (9674.06 to 26451.01) | 658799.39 (428622.48 to 991559.89) | 6363.22 (3862.1 to 9901.56) | -2.6  (-3.45 to -1.75) |
| South Africa | 461828.59 (358641.32 to 594662.75) | 3513.21 (2604.34 to 4725.04) | 173617.95 (134413.46 to 220919.04) | 1175.25 (839.26 to 1640.13) | -3.26  (-3.88 to -2.63) |
| South Sudan | 646956.68 (377751.45 to 947803.05) | 22587.15 (12819.72 to 33287.64) | 206400.67 (139925.87 to 297829.38) | 4803.38 (3029.99 to 7359.94) | -5.49  (-5.71 to -5.28) |
| Spain | 17678.7 (8972.65 to 29933.25) | 243.99 (101.7 to 475.04) | 9482.4 (4810.53 to 17510.9) | 144.12 (58.02 to 319.34) | -1.91  (-2.08 to -1.73) |
| Sri Lanka | 59984.53 (42578.65 to 83285.76) | 1101.78 (741.6 to 1574.76) | 22290.39 (14299.73 to 33254.62) | 457.98 (265.71 to 734.01) | -3.36  (-3.52 to -3.2) |
| Sudan | 445364.86 (231910.84 to 855536) | 4356.86 (2145.87 to 8529.87) | 206663.73 (140871.27 to 290908.7) | 1308.79 (822.94 to 1975.67) | -4.23  (-4.33 to -4.14) |
| Suriname | 2221.68 (1626.8 to 2877.2) | 1735.04 (1184.26 to 2468.89) | 1170.25 (775.35 to 1693.13) | 827.19 (482.38 to 1333.36) | -2.65  (-2.73 to -2.56) |
| Sweden | 2039.88 (1125.52 to 3327.44) | 133.23 (61.11 to 262.54) | 1886.75 (1043.91 to 3122.33) | 104.12 (48.23 to 202.12) | -1.12  (-1.25 to -0.98) |
| Switzerland | 1472.74 (846.62 to 2498.07) | 128.22 (59.78 to 266.59) | 1080.34 (590.08 to 1866.23) | 82.47 (36.1 to 170.21) | -1.63  (-1.8 to -1.45) |
| Syrian Arab Republic | 83590.25 (60567.59 to 112214.34) | 1315.31 (887.41 to 1924.15) | 21615.19 (14135.6 to 31864.23) | 590.8 (346.74 to 958.85) | -2.62  (-3.01 to -2.22) |
| Taiwan (Province of China) | 15502.36 (9268.25 to 24896.34) | 291.67 (145.16 to 529.22) | 4396.85 (2375.71 to 7472.2) | 147.48 (66.76 to 292.32) | -2.38  (-2.62 to -2.13) |
| Tajikistan | 22706.6 (14943.72 to 31896.55) | 922.48 (566.4 to 1418.74) | 19684.99 (12684.87 to 28502.89) | 594.39 (333.14 to 958.6) | -2.27  (-3.08 to -1.46) |
| Thailand | 98762.9 (70700.53 to 137550.34) | 612.97 (411.47 to 878.86) | 24807.65 (14973.81 to 40268.98) | 255.06 (135.95 to 456.32) | -3.04 (-3.22 to -2.85) |
| Timor-Leste | 41997.9 (10783.55 to 73134.54) | 10552.4 (2698.29 to 18506.71) | 6477.99 (4498.44 to 8936.81) | 1288.38 (816.42 to 1922.99) | -7.82 (-8.29 to -7.34) |
| Togo | 87086.54 (57779.22 to 128602.04) | 4503.6 (2771.46 to 6962.19) | 55853.83 (37492.29 to 80170.61) | 1761.81 (1119.79 to 2631.87) | -3.72 (-4.08 to -3.35) |
| Tokelau | 11.43 (7.99 to 15.95) | 1457.26 (911.77 to 2205.83) | 3.23 (2.1 to 4.7) | 664.48 (378.58 to 1076.41) | -2.83 (-2.98 to -2.68) |
| Tonga | 377.53 (269.84 to 515.33) | 976.86 (638.32 to 1426.24) | 221.02 (153.94 to 318.69) | 626.15 (381.09 to 988.57) | -1.51 (-1.6 to -1.42) |
| Trinidad and Tobago | 3429.94 (2253.05 to 5018.22) | 856.22 (501.72 to 1392.99) | 1465.83 (851.3 to 2328.47) | 553.51 (270.33 to 991.59) | -1.77 (-1.92 to -1.62) |
| Tunisia | 17752.54 (12243.21 to 24799.06) | 550.17 (352.42 to 830.54) | 6481.06 (3913.06 to 10087.43) | 251.7 (136.65 to 441.51) | -2.59 (-2.75 to -2.43) |
| Turkey | 235170.48 (162570.88 to 337612.81) | 1088.49 (660.63 to 1701.56) | 52698.34 (32171.46 to 84917.91) | 350.06 (172.77 to 631.87) | -4.17 (-4.41 to -3.93) |
| Turkmenistan | 13824.96 (9924.77 to 19246.92) | 876.31 (564.13 to 1296.9) | 7476.29 (4647.21 to 11097.3) | 484.13 (259.74 to 804.88) | -2.08 (-2.14 to -2.03) |
| Tuvalu | 111.26 (76.81 to 155.76) | 3008.12 (1916.91 to 4473.27) | 26.74 (17.63 to 38.68) | 819.01 (472.24 to 1289.04) | -4.35 (-4.67 to -4.02) |
| Uganda | 697130.99 (429180.07 to 1109556.13) | 6978.67 (4115.81 to 11319.69) | 392154.97 (256862.01 to 552118.27) | 1977.51 (1195.26 to 3004.25) | -4.15 (-4.4 to -3.89) |
| Ukraine | 34313.99 (24328.19 to 48580.07) | 310.81 (194.74 to 485.84) | 13920.41 (8958.35 to 20904.79) | 211.64 (124.26 to 360.25) | -2.01 (-2.29 to -1.74) |
| United Arab Emirates | 3890.67 (2466.9 to 5798.48) | 629.08 (351.81 to 1046.92) | 4430.18 (2556.69 to 6992.36) | 396.64 (198.65 to 686.55) | -1.36 (-1.48 to -1.24) |
| United Kingdom | 28832.73 (17620.72 to 41836.38) | 263.45 (150.26 to 417.13) | 24610.64 (14925.68 to 37629.98) | 209.75 (114.78 to 363.78) | -0.99 (-1.08 to -0.9) |
| United Republic of Tanzania | 1136747.3 (831551.74 to 1517698.04) | 8380.92 (5858.12 to 11628.79) | 708717.48 (500231.81 to 949492.85) | 2757.07 (1787.02 to 4025.39) | -4.07 (-4.27 to -3.87) |
| United States of America | 53682.54 (33349.43 to 81061.03) | 96.57 (54.95 to 157.76) | 55289.72 (32929.5 to 88271.8) | 91.73 (45.79 to 164.17) | -0.17 (-0.5 to 0.17) |
| United States Virgin Islands | 237.36 (157.57 to 346.01) | 754.45 (442.46 to 1214.82) | 86.44 (50.79 to 138.45) | 442.09 (216.28 to 814.97) | -1.89 (-2.09 to -1.7) |
| Uruguay | 6331.68 (4679.64 to 8649.02) | 800.73 (545.5 to 1190.58) | 2283.88 (1399.13 to 3581.32) | 337.98 (182.54 to 602.03) | -2.83 (-3.02 to -2.63) |
| Uzbekistan | 121717.15 (83490.77 to 172046.2) | 1363.1 (858.86 to 2048.5) | 97493.68 (61402.59 to 143828.01) | 941.49 (514.26 to 1510.57) | -1.33 (-1.37 to -1.3) |
| Vanuatu | 965.63 (674.3 to 1344.91) | 1341.95 (864.08 to 2031.19) | 1116.36 (757.55 to 1537.63) | 1019.17 (627.37 to 1522.58) | -1.03 (-1.24 to -0.81) |
| Venezuela (Bolivarian Republic of) | 71147.38 (59789.14 to 86256.2) | 982.07 (781.01 to 1248.22) | 29231.1 (20858.8 to 39966.36) | 434.5 (285.82 to 646.68) | -3.92 (-4.62 to -3.21) |
| Viet Nam | 293310.16 (209152.26 to 402726.68) | 1117.09 (717.4 to 1652.07) | 73557.95 (46591.44 to 109889.18) | 355.05 (203.55 to 575.85) | -4.15 (-4.25 to -4.06) |
| Yemen | 442141.68 (204518.72 to 1191712.98) | 5522.1 (2470.88 to 14908.9) | 288253.35 (198058.03 to 419670.66) | 2243.29 (1482.06 to 3382.67) | -3.16 (-3.39 to -2.93) |
| Zambia | 389165.63 (273808.09 to 540112.48) | 9260.52 (6162.07 to 13332.74) | 237224.67 (173853.54 to 313672.37) | 3024.18 (2035.86 to 4338.69) | -3.71 (-3.83 to -3.58) |
| Zimbabwe | 163636.09 (103730.2 to 238083.54) | 3264.45 (1954.38 to 4902.08) | 195364.44 (130406.05 to 273090.67) | 3240.04 (1988.28 to 4785.67) | 1.98 (1.08 to 2.89) |

**Supplementary Table 6** Global and regional burden of iodine deficiency among children in 2019

| Locations | Prevalent Cases (95%UI) | ASPR/100,000 (95% CI) | Incident cases (95%UI) | ASIR/100,000 (95% CI) | DALY cases | Age-standardized DALY rate |
| --- | --- | --- | --- | --- | --- | --- |
| Global | 12110179.48 (8119587.79 to 17101462.65) | 604.14 (403.46 to 856.81) | 2568080.01 (1817082.15 to 3485847.07) | 129.2 (90.79 to 177.2) | 240953.95 (135489.43 to 399709.29) | 12.03 (6.71 to 20.17) |
| SDI regions |  |  |  |  |  |  |
| High SDI | 179635.82 (106325.72 to 276179.96) | 104.2 (61.53 to 160.08) | 37393.21 (23118.95 to 56574.49) | 22.19 (13.59 to 33.24) | 1966.72 (784.84 to 3983.58) | 1.14 (0.46 to 2.32) |
| High-middle SDI | 566107.6 (363192.71 to 840566.34) | 224.84 (144.32 to 333.17) | 124390.49 (86525.55 to 176861.43) | 49.84 (33.81 to 70.37) | 7995.54 (3887.58 to 14797.33) | 3.18 (1.52 to 5.85) |
| Middle SDI | 1703748.71 (1098327.38 to 2514393.31) | 297.54 (191.98 to 438.62) | 376393.34 (263883.42 to 526873.92) | 66.44 (45.57 to 93.17) | 27530.15 (14610.02 to 48923.71) | 4.81 (2.52 to 8.56) |
| Low-middle SDI | 3767717.31 (2480863.29 to 5460611.77) | 690.11 (450.38 to 1000.23) | 811260.38 (570599.07 to 1112569.45) | 150.63 (103.84 to 210.32) | 84632.97 (48236.87 to 137163.53) | 15.52 (8.75 to 25.42) |
| Low SDI | 5889500.76 (3971517.13 to 8153951.74) | 1275.4 (861.44 to 1762.91) | 1217923.61 (880004.72 to 1610385.89) | 261.41 (185.36 to 349.44) | 118753.22 (67359.74 to 196963.17) | 25.68 (14.4 to 42.5) |
| GBD regions |  |  |  |  |  |  |
| Andean Latin America | 4633.57 (2206.14 to 7900.97) | 25.65 (12.29 to 43.72) | 949.78 (488.97 to 1592.61) | 5.26 (2.68 to 8.69) | 63.13 (24.54 to 130.06) | 0.35 (0.13 to 0.72) |
| Australasia | 4129.12 (2359.76 to 6376.98) | 72.87 (41.6 to 112.26) | 835.36 (489.9 to 1263.63) | 14.97 (8.83 to 22.64) | 44.77 (17.4 to 91.77) | 0.79 (0.3 to 1.67) |
| Caribbean | 31131.94 (17124.01 to 48606.19) | 261.13 (142.63 to 405.51) | 5971.22 (3479.44 to 9126.81) | 50.6 (29.45 to 76.69) | 659.02 (296.56 to 1208.78) | 5.53 (2.39 to 10.36) |
| Central Asia | 64552.51 (43228.41 to 90505.2) | 246.52 (165.69 to 346.33) | 12602.42 (8531.32 to 17510.02) | 47.2 (32.05 to 65.57) | 1153.44 (590.5 to 1995.68) | 4.4 (2.14 to 7.79) |
| Central Europe | 19627.73 (12428.34 to 28395.15) | 105.45 (66.52 to 152.82) | 3787.03 (2429.68 to 5419.01) | 20.9 (13.33 to 30.12) | 214.44 (88.93 to 433.73) | 1.15 (0.47 to 2.34) |
| Central Latin America | 233162.72 (155282.64 to 327611.45) | 343.38 (227.23 to 482.19) | 44276.5 (29267.05 to 61806.3) | 66.6 (44.09 to 93.29) | 3535.18 (1726.1 to 6379.09) | 5.21 (2.53 to 9.48) |
| Central Sub-Saharan Africa | 3025669.1 (2093816.96 to 3989100.6) | 5478.62 (3785.45 to 7239.22) | 574793.08 (408347.42 to 751909.2) | 1020.33 (723.23 to 1327.74) | 41619.4 (21111.67 to 75559.03) | 75.03 (38.05 to 136.95) |
| East Asia | 368397.53 (220031.39 to 590488.73) | 159.72 (94.52 to 258.92) | 95960.22 (66872.96 to 142051.11) | 41.54 (27.57 to 61.16) | 4040.08 (1617.12 to 8450.04) | 1.75 (0.7 to 3.65) |
| Eastern Europe | 51376.8 (34972.77 to 71281.66) | 137.14 (93.23 to 190.53) | 9736.63 (6618.27 to 13477.5) | 25.93 (17.55 to 36.23) | 1118.91 (565.21 to 1862.21) | 2.99 (1.49 to 5.1) |
| Eastern Sub-Saharan Africa | 1355074.22 (867524.23 to 2008277.9) | 788.37 (505.73 to 1172.02) | 310443.99 (218422.14 to 431055.51) | 179.35 (123.99 to 253.18) | 27055.83 (14498.2 to 45319.17) | 15.71 (8.32 to 26.58) |
| High-income Asia Pacific | 22206.68 (12869.18 to 33782.93) | 89.31 (51.69 to 135.95) | 4442.78 (2632.46 to 6678.11) | 18.36 (10.76 to 27.54) | 242.5 (94.14 to 509.15) | 0.98 (0.38 to 2.03) |
| High-income North America | 56744.93 (32611.05 to 87653.9) | 79.97 (46.12 to 123.38) | 11278.33 (6706.25 to 17286.96) | 16.44 (9.63 to 25.07) | 619.32 (242.55 to 1296.16) | 0.87 (0.34 to 1.83) |
| North Africa and Middle East | 949003.41 (662517.61 to 1291809.15) | 532.09 (372.44 to 722.07) | 173594.14 (119047.63 to 233276.37) | 97.95 (67.08 to 131.8) | 24680.75 (14314.62 to 39748.57) | 13.84 (7.96 to 22.37) |
| Oceania | 856.14 (373.62 to 1536.78) | 18.81 (8.35 to 33.7) | 179.21 (89.84 to 306.99) | 3.85 (1.9 to 6.6) | 21.2 (7.53 to 43.16) | 0.47 (0.16 to 0.97) |
| South Asia | 4881807.58 (3116618.26 to 7125750.27) | 887.18 (565.43 to 1299.87) | 1087008.42 (767848.41 to 1490562.13) | 200.91 (138.53 to 279.89) | 118981.42 (67481.01 to 191456.3) | 21.66 (12.14 to 35.31) |
| Southeast Asia | 147421.66 (88892.36 to 236693.49) | 82.76 (49.3 to 132.77) | 41121.74 (29319.78 to 59042.43) | 23.22 (15.31 to 33.96) | 2904.03 (1387.57 to 5158.12) | 1.63 (0.78 to 2.94) |
| Southern Latin America | 10148.24 (5705.83 to 15013.87) | 65.75 (37.06 to 97.08) | 2011.22 (1165.03 to 2951.4) | 13.21 (7.62 to 19.46) | 110.73 (43.42 to 225.5) | 0.72 (0.27 to 1.47) |
| Southern Sub-Saharan Africa | 77830.97 (46501.63 to 120439.69) | 327.11 (195.28 to 505.24) | 16346.85 (10273.56 to 23998.38) | 68.85 (42.95 to 102.42) | 910.95 (381.71 to 1857.48) | 3.83 (1.59 to 7.88) |
| Tropical Latin America | 19444.62 (10076.1 to 32541.55) | 37.28 (19.46 to 62.13) | 3910.68 (2174.64 to 6282.31) | 7.67 (4.16 to 12.37) | 212.02 (79.81 to 451.27) | 0.41 (0.15 to 0.86) |
| Western Europe | 141324.32 (85769.23 to 217570.31) | 194.23 (116.64 to 299.43) | 31688.04 (20928.65 to 46709.3) | 44.29 (28.39 to 65.51) | 1542.03 (606.45 to 3165.83) | 2.12 (0.83 to 4.36) |
| Western Sub-Saharan Africa | 645635.69 (389552.02 to 991921.14) | 336.76 (203.04 to 516.65) | 137142.39 (87880.16 to 200706.03) | 70.61 (44.6 to 103.42) | 11224.81 (5551.87 to 20076.15) | 5.85 (2.85 to 10.58) |

**Supplementary Table 7** Global and regional burden of iron deficiency among children in 2019

| Locations | Prevalent Cases (95%UI) | ASPR/100,000  (95% CI) | Incident cases (95%UI) | ASIR/100,000  (95% CI) | DALY cases | Age-standardized DALY rate |
| --- | --- | --- | --- | --- | --- | --- |
| Global | 391491699.23 (382834300.61 to 400499411.06) | 20146.35 (19407.85 to 20888.54) | NA | NA | 13620230.64 (9174233.54 to 19794594.83) | 698.9 (466.54 to 1015.31) |
| SDI regions |  |  |  |  |  |  |
| High SDI | 7371084.95 (6492145.97 to 8365813.21) | 4671.08 (3765.88 to 5786.17) | NA | NA | 149955.44 (93839.06 to 231790.76) | 93.68 (55.55 to 149.37) |
| High-middle SDI | 22437432.98 (21042722.77 to 23933637.88) | 9271.51 (8331.91 to 10291.1) | NA | NA | 618841.96 (402987.94 to 911054.32) | 254.57 (162.91 to 385.17) |
| Middle SDI | 74903749.56 (71788498.78 to 77947951.98) | 13720.54 (12825.23 to 14632.6) | NA | NA | 2336857.73 (1555153.16 to 3391386.24) | 425.65 (282.68 to 624.46) |
| Low-middle SDI | 131645173.97 (126737773.98 to 136926308.21) | 25497.06 (24067.64 to 27050.28) | NA | NA | 4611592.66 (3079567.4 to 6738220.99) | 887.81 (585.77 to 1297.56) |
| Low SDI | 154901334.64 (150630641.37 to 159216170.24) | 32516.96 (30993.53 to 34036.61) | NA | NA | 5895716.72 (3944808.94 to 8616020.1) | 1240.83 (819.96 to 1813.62) |
| GBD regions |  |  |  |  |  |  |
| Andean Latin America | 2686720.94 (2298807.32 to 3102904.35) | 14834.8 (11554.93 to 18852.94) | NA | NA | 70861.47 (44016.3 to 110157.65) | 391.54 (224.06 to 640.84) |
| Australasia | 378781.56 (255618.87 to 550578.67) | 7152.01 (4056.56 to 12186.55) | NA | NA | 6195.33 (3133.51 to 11129.22) | 116.21 (47.28 to 244.48) |
| Caribbean | 2488561.19 (2256989.54 to 2724735.35) | 21502.95 (18148.53 to 25052.68) | NA | NA | 75295.5 (48559.93 to 113864.02) | 648.64 (391.18 to 1006.31) |
| Central Asia | 5232945.13 (4653920 to 5800774.5) | 19244.32 (15835.29 to 22913.75) | NA | NA | 154800.5 (98709.2 to 227226.4) | 570.05 (340.27 to 877.65) |
| Central Europe | 1664081.59 (1456146.38 to 1911562.33) | 9797.95 (7831.49 to 12275.83) | NA | NA | 39486.66 (24252.71 to 60494.09) | 229.62 (133.09 to 368.52) |
| Central Latin America | 5804863.12 (5379536.02 to 6271630.9) | 9114.43 (8124.54 to 10266.49) | NA | NA | 143777.06 (93522.31 to 210590.62) | 224.15 (142.47 to 336.01) |
| Central Sub-Saharan Africa | 15180080.69 (13477412.75 to 16882954.38) | 26308.13 (21812.73 to 31108.19) | NA | NA | 496116.51 (315481.14 to 735381.39) | 863.04 (516.59 to 1321.32) |
| East Asia | 8555501.51 (7185285.86 to 10167906.68) | 3629.73 (2723.62 to 4711.96) | NA | NA | 206133.79 (127438.05 to 317068.12) | 87.97 (47.57 to 145.28) |
| Eastern Europe | 1799697 (1330382.64 to 2402253.96) | 4951.41 (3124.79 to 7738.71) | NA | NA | 35945.1 (20388.75 to 59327.65) | 97.8 (47.08 to 183.49) |
| Eastern Sub-Saharan Africa | 47854083.91 (45913937.72 to 49880313.35) | 26843.23 (25055.24 to 28692.6) | NA | NA | 1653748.53 (1104166.98 to 2426650.9) | 931.01 (613.91 to 1366.16) |
| Global | 391491699.23 (382834300.61 to 400499411.06) | 20146.35 (19407.85 to 20888.54) | NA | NA | 13620230.64 (9174233.54 to 19794594.83) | 698.9 (466.54 to 1015.31) |
| High-income Asia Pacific | 1426249.47 (1050589.26 to 1879055.29) | 6350.17 (3884.4 to 10061.75) | NA | NA | 31930.17 (18161.51 to 52886.74) | 139.62 (65.88 to 258.26) |
| High-income North America | 2330753.13 (1678762.64 to 3118717.59) | 3618.47 (2053.33 to 5826.49) | NA | NA | 45707.74 (24972.58 to 78926.71) | 69.36 (30.06 to 133.63) |
| North Africa and Middle East | 26452064.6 (24566356.7 to 28671263.26) | 15187.7 (13391.61 to 17160.04) | NA | NA | 786372.8 (515429.96 to 1178235.7) | 450.83 (283.09 to 684.98) |
| Oceania | 1111810.99 (962617.26 to 1258941.72) | 22370.1 (17445.45 to 27781.41) | NA | NA | 37276.48 (23079.6 to 55039.4) | 756.95 (441.32 to 1199.61) |
| South Asia | 160105820.65 (153978399.23 to 166417752.19) | 31523.88 (29637.75 to 33458.24) | NA | NA | 6046176.4 (4004177.84 to 8790538.78) | 1180.06 (775.65 to 1732.25) |
| Southeast Asia | 20925216.27 (18966205.38 to 22979648.51) | 12736.34 (10952.83 to 14777.29) | NA | NA | 569630.48 (358447.8 to 864036.43) | 343.49 (212.38 to 529.35) |
| Southern Latin America | 1456907.44 (1113640.66 to 1833745.98) | 10144.72 (6595.32 to 14813.74) | NA | NA | 31920.77 (18627.47 to 51889.33) | 220.66 (109.36 to 413.86) |
| Southern Sub-Saharan Africa | 3605687.95 (3152947.92 to 4109775.01) | 15304.75 (12325.12 to 18938.37) | NA | NA | 106635.7 (67522.64 to 159646.54) | 451.45 (264.25 to 704.9) |
| Tropical Latin America | 6088968.75 (4840166.53 to 7543103.73) | 12536.16 (8376.69 to 17678.4) | NA | NA | 182219.01 (110308.6 to 283954.34) | 372.9 (193.08 to 648.09) |
| Western Europe | 2784386.85 (2296580.05 to 3337399.75) | 4219.94 (3198.71 to 5551.05) | NA | NA | 52145.66 (31613.08 to 81175.06) | 77.99 (44.32 to 129.03) |
| Western Sub-Saharan Africa | 73558516.47 (69129405.58 to 77971916.44) | 36715.5 (33278.85 to 40025.55) | NA | NA | 2847854.97 (1885088.17 to 4113576.07) | 1425.3 (921.99 to 2097.08) |

**Supplementary Table 8** Global and regional burden of protein-energy malnutrition among children in 2019

| Locations | Prevalent Cases (95%UI) | ASPR/100,000 (95% CI) | Incident cases (95%UI) | ASIR/100,000 (95% CI) | DALY cases | Age-standardized DALY rate |
| --- | --- | --- | --- | --- | --- | --- |
| Global | 72727765.96 (68933020.63 to 77526486.27) | 3796.01 (3584.19 to 4063.58) | 49762816.45 (45620846.43 to 55096112.33) | 3874.04 (3009.59 to 4975.13) | 10874288.94 (8809866.22 to 13400280.04) | 570.08 (458.83 to 707.64) |
| SDI regions |  |  |  |  |  |  |
| High SDI | 1471698.42 (1250104.24 to 1764417.41) | 926.07 (782.3 to 1108.86) | 1434011.88 (1191200.74 to 1754738.2) | 1179.79 (871.11 to 1634.87) | 36034.23 (21447.72 to 55261.49) | 21.74 (12.88 to 33.78) |
| High-middle SDI | 5842736.38 (5338653.02 to 6485014.56) | 2432.83 (2197.52 to 2729.05) | 4619678.24 (4080681.98 to 5315986.86) | 1913.94 (1673.74 to 2222.97) | 212404.34 (152129.3 to 278293.64) | 88.38 (63.51 to 118.92) |
| Middle SDI | 17663085.04 (16412447.16 to 19206948.13) | 3292.9 (3049.68 to 3607.76) | 13304634.99 (11967842.56 to 15071413.11) | 2463.88 (2210.38 to 2788.93) | 1104071.02 (882235.83 to 1337357.79) | 206.85 (164.04 to 255.07) |
| Low-middle SDI | 25102290.45 (23894967.03 to 26615434.16) | 5009.55 (4734.76 to 5340.25) | 16964151.15 (15618154.32 to 18669937.78) | 3358.24 (3079.95 to 3707.78) | 2772206.73 (2233699.57 to 3388432.81) | 557.11 (444.47 to 688.72) |
| Low SDI | 22611376.14 (21917772.25 to 23441032.94) | 4641.11 (4457.26 to 4858.39) | 13419064.36 (12694527.39 to 14302105.84) | 2766.15 (2586.15 to 2983.88) | 6742633.02 (5230049.45 to 8668358.28) | 1380.37 (1063.59 to 1780.7) |
| GBD regions |  |  |  |  |  |  |
| Andean Latin America | 119029.18 (111759.85 to 127747.89) | 655.89 (603.16 to 720.12) | 89227.04 (81965.75 to 98556.89) | 673.11 (555.17 to 826.36) | 42657.33 (29572.14 to 58467.28) | 234.98 (158.9 to 325.73) |
| Australasia | 41897.54 (38199.19 to 46889.72) | 790.28 (698.17 to 911.23) | 49143.05 (44902.45 to 54828.39) | 995.52 (870.23 to 1169.82) | 611.11 (373.96 to 971.89) | 11.16 (6.13 to 18.69) |
| Caribbean | 187077.86 (177863.83 to 198568.56) | 1641.97 (1526.71 to 1778.11) | 94421.95 (86214.55 to 105325.84) | 1717.36 (1261.42 to 2305.52) | 80638.61 (52397.75 to 122420.03) | 709.51 (450.33 to 1089.9) |
| Central Asia | 623161.41 (595673.7 to 653864.46) | 2274.95 (2133.76 to 2433.66) | 389498.74 (366874.62 to 416740.65) | 2062.16 (1653.7 to 2642.27) | 19324.84 (13128.08 to 26642.96) | 70.62 (47.39 to 99.52) |
| Central Europe | 261635.69 (238481.06 to 291024.95) | 1564.52 (1401.33 to 1756.47) | 194377.6 (171109.7 to 225487.97) | 2018.74 (1410.77 to 2921.04) | 5697.84 (3772.99 to 8106.16) | 33.28 (21.47 to 48.85) |
| Central Latin America | 826515.35 (754558.92 to 922945.7) | 1302.76 (1176.16 to 1468.34) | 680479.83 (600634.58 to 790035.19) | 1457.92 (1145.07 to 1880.01) | 158542.17 (119426.98 to 206202.46) | 252.69 (188.67 to 331.36) |
| Central Sub-Saharan Africa | 2087459.16 (1998952.59 to 2180298.65) | 3524.62 (3338.3 to 3727.63) | 858505.97 (813721.23 to 905590.44) | 2678.98 (2077.83 to 3485.01) | 609574.4 (409321.51 to 882987.62) | 1030.07 (681.94 to 1511.99) |
| East Asia | 4351559.05 (3733461 to 5135554.81) | 1845.17 (1514.95 to 2259.14) | 3709885.48 (3064489.66 to 4548534.58) | 2371.44 (1660.9 to 3395.07) | 110768.26 (81794.86 to 148975.21) | 47.08 (32.5 to 66.71) |
| Eastern Europe | 738204.04 (685412.32 to 799399.32) | 2059.04 (1838.02 to 2297.33) | 552977.33 (505159.91 to 608462.45) | 2303.93 (1757.16 to 3073.58) | 16244.88 (10673.06 to 22948.08) | 44.57 (28.23 to 66.31) |
| Eastern Sub-Saharan Africa | 5743861.85 (5629586.82 to 5881301.14) | 3134.42 (3040.22 to 3243.67) | 2835302.53 (2735922.77 to 2961364.95) | 2628.85 (2074.25 to 3346.72) | 2974981.97 (2274513.58 to 3852464.58) | 1624.68 (1235.27 to 2122.22) |
| High-income Asia Pacific | 246057.47 (221850.74 to 279181.1) | 1125.82 (1011.86 to 1270.41) | 255471.28 (229647.08 to 289908.61) | 1294.99 (1107.7 to 1563.41) | 3255.76 (2009.41 to 4884.31) | 14.02 (8.49 to 21.44) |
| High-income North America | 339744.98 (261877.07 to 444143.51) | 513.18 (385.66 to 680.41) | 352582.38 (266279.32 to 464097.92) | 662.71 (454.14 to 966.45) | 9378.63 (5448.07 to 15015.92) | 13.75 (7.99 to 21.91) |
| North Africa and Middle East | 5454784.32 (5166671.95 to 5769418.09) | 3166.51 (2978.18 to 3392.51) | 4105657.98 (3824353.82 to 4455535.59) | 3168.02 (2630.79 to 3885.44) | 359754.62 (267371.62 to 494041.3) | 209.01 (154 to 292.8) |
| Oceania | 223824.67 (213252.15 to 235136.95) | 4252.22 (3990.29 to 4530.56) | 103581.73 (97786.65 to 109973.65) | 3840.09 (2889.01 to 5106.26) | 18776.45 (13733.03 to 25968.52) | 357.45 (258.98 to 502.3) |
| South Asia | 34174597.85 (32267711.66 to 36551526) | 7069.86 (6645.26 to 7577.03) | 24278695.8 (22182795.22 to 26939767.44) | 7660.09 (5927.69 to 9824.29) | 2775158.64 (2173453.96 to 3542709.85) | 581.12 (450.72 to 752.21) |
| Southeast Asia | 6724680.11 (6372986.16 to 7149001.74) | 4224.77 (3972.18 to 4531.13) | 4614570.85 (4240410.04 to 5098653.59) | 4083.51 (3238.72 to 5229.78) | 395704.67 (312539.09 to 490537.1) | 248.83 (194.77 to 310.54) |
| Southern Latin America | 130243.14 (115900.12 to 148311.76) | 907.86 (780.05 to 1063.42) | 123864.92 (108386.55 to 144917.93) | 1002.15 (820.82 to 1234.78) | 6971.07 (5462.26 to 8757.19) | 49.01 (36.77 to 64.41) |
| Southern Sub-Saharan Africa | 490650.48 (473463.13 to 512612.41) | 2107.55 (2004.67 to 2230.07) | 269627.99 (253095.2 to 290082.01) | 2027.75 (1535.83 to 2699.75) | 307270.22 (222898.86 to 408922.09) | 1321.39 (946.66 to 1764.27) |
| Tropical Latin America | 577968.38 (546189.98 to 618661.9) | 1229.53 (1150.02 to 1328.63) | 427226.53 (394121.32 to 471422.39) | 1131.91 (945.51 to 1393.36) | 98519.61 (78767.82 to 120698.96) | 210.91 (167.3 to 262.35) |
| Western Europe | 680602.57 (554117.44 to 842881.53) | 1003.02 (813.19 to 1241.8) | 709900.29 (572905.12 to 889646.47) | 1313.71 (959.25 to 1832.37) | 21147.38 (11965.16 to 33656.12) | 29.59 (16.64 to 47.42) |
| Western Sub-Saharan Africa | 8704210.87 (8472114.99 to 8980616.91) | 4196.94 (4030.15 to 4390.9) | 5067817.18 (4853823.58 to 5331800.92) | 3524.45 (2893.87 to 4358.37) | 2859310.47 (2097410.31 to 3788198.89) | 1374.75 (1005.33 to 1830.01) |

**Supplementary Table 9** Global and regional burden of Vitamin A deficiency among children in 2019

| Locations | Prevalent Cases (95%UI) | ASPR/100,000  (95% CI) | Incident cases (95%UI) | ASIR/100,000  (95% CI) | DALY cases | Age-standardized DALY rate |
| --- | --- | --- | --- | --- | --- | --- |
| Global | 209674096.57 (196241630.19 to 225107157.08) | 10779.02 (9727.6 to 12133.06) | 209674096.57 (196241630.19 to 225107157.08) | 10779.02 (9727.6 to 12133.06) | 992430.71 (674034.16 to 1388337.83) | 51.19 (34.22 to 72.86) |
| SDI regions |  |  |  |  |  |  |
| High SDI | 1656869.15 (1471547.43 to 1878062.47) | 1030.23 (848.47 to 1263.04) | 1656869.15 (1471547.43 to 1878062.47) | 1030.23 (848.47 to 1263.04) | 1427.69 (896.67 to 2169.15) | 0.9 (0.52 to 1.44) |
| High-middle SDI | 8688886.2 (7845655.6 to 9636908.18) | 3558.31 (2988.12 to 4252.64) | 8688886.2 (7845655.6 to 9636908.18) | 3558.31 (2988.12 to 4252.64) | 23222.11 (15496.83 to 33780.83) | 9.58 (6.07 to 14.38) |
| Middle SDI | 32357110.71 (29305729.77 to 35668673.24) | 5905.03 (4942.86 to 7035.82) | 32357110.71 (29305729.77 to 35668673.24) | 5905.03 (4942.86 to 7035.82) | 113977.37 (75713.62 to 165505.89) | 20.9 (13.57 to 31) |
| Low-middle SDI | 60085016.5 (53253053.98 to 68333424.26) | 11631.03 (9576.04 to 14527.17) | 60085016.5 (53253053.98 to 68333424.26) | 11631.03 (9576.04 to 14527.17) | 284192.5 (189084.19 to 407345.43) | 55.36 (35.24 to 81.65) |
| Low SDI | 106741855.57 (100467847.19 to 113414825.93) | 22389.56 (20252.91 to 24796.64) | 106741855.57 (100467847.19 to 113414825.93) | 22389.56 (20252.91 to 24796.64) | 569021.68 (393361.01 to 802235.54) | 119.05 (80.27 to 168.46) |
| GBD regions |  |  |  |  |  |  |
| Andean Latin America | 1256009.87 (1041363.85 to 1524120.48) | 6941.17 (5096.15 to 9480.62) | 1256009.87 (1041363.85 to 1524120.48) | 6941.17 (5096.15 to 9480.62) | 4401.23 (2851.93 to 6464.68) | 24.32 (14.49 to 38.56) |
| Australasia | 8225.37 (6300.66 to 10682.51) | 150.1 (95.19 to 229.67) | 8225.37 (6300.66 to 10682.51) | 150.1 (95.19 to 229.67) | 9.62 (4.87 to 17.24) | 0.18 (0.07 to 0.4) |
| Caribbean | 953341.41 (780069.99 to 1164381.43) | 8200.42 (5902.23 to 11367.72) | 953341.41 (780069.99 to 1164381.43) | 8200.42 (5902.23 to 11367.72) | 4642.73 (2986.41 to 6963.39) | 40.09 (23.01 to 64.57) |
| Central Asia | 1469785.46 (1250519.12 to 1713227) | 5427.84 (4202.62 to 7065.81) | 1469785.46 (1250519.12 to 1713227) | 5427.84 (4202.62 to 7065.81) | 5731.71 (3701.36 to 8331.26) | 21.06 (12.97 to 32.36) |
| Central Europe | 1461865.48 (1314992.64 to 1635077.48) | 8336.12 (6943.67 to 10032.26) | 1461865.48 (1314992.64 to 1635077.48) | 8336.12 (6943.67 to 10032.26) | 2122.6 (1321.12 to 3233.31) | 12.4 (7.17 to 20) |
| Central Latin America | 4758078.08 (3963280.35 to 5732788.02) | 7336.48 (5345.24 to 10226.18) | 4758078.08 (3963280.35 to 5732788.02) | 7336.48 (5345.24 to 10226.18) | 11780.21 (7630.91 to 17642.68) | 18.33 (11.32 to 27.91) |
| Central Sub-Saharan Africa | 17980935.82 (15382342.96 to 20726591.67) | 31206.7 (23673.5 to 39486.02) | 17980935.82 (15382342.96 to 20726591.67) | 31206.7 (23673.5 to 39486.02) | 89366.5 (58597.65 to 127731.69) | 154.48 (93.91 to 235.5) |
| East Asia | 8579280.25 (6515241.79 to 11436606.52) | 3682.78 (2276.86 to 5901.61) | 8579280.25 (6515241.79 to 11436606.52) | 3682.78 (2276.86 to 5901.61) | 19967.79 (12338.67 to 29703.39) | 8.52 (5.12 to 13.18) |
| Eastern Europe | 169575.21 (140409.12 to 206239.5) | 459.34 (332.43 to 634.1) | 169575.21 (140409.12 to 206239.5) | 459.34 (332.43 to 634.1) | 134.39 (75.58 to 224.9) | 0.37 (0.18 to 0.71) |
| Eastern Sub-Saharan Africa | 44610382.16 (41104406.3 to 48133060.96) | 25052.34 (21931.14 to 28528.86) | 44610382.16 (41104406.3 to 48133060.96) | 25052.34 (21931.14 to 28528.86) | 203713.97 (137400.74 to 290051.49) | 113.96 (75.33 to 164.97) |
| High-income Asia Pacific | 305881.21 (242233.27 to 386910.08) | 1324.65 (874.9 to 1946.34) | 305881.21 (242233.27 to 386910.08) | 1324.65 (874.9 to 1946.34) | 280.34 (148.06 to 480) | 1.23 (0.53 to 2.54) |
| High-income North America | 626721.21 (476113.13 to 821993.22) | 968.26 (621.1 to 1508.18) | 626721.21 (476113.13 to 821993.22) | 968.26 (621.1 to 1508.18) | 336.96 (171.03 to 602.08) | 0.52 (0.2 to 1.12) |
| North Africa and Middle East | 14418460.35 (13018070.05 to 15873295.58) | 8262.99 (6892.71 to 9734.23) | 14418460.35 (13018070.05 to 15873295.58) | 8262.99 (6892.71 to 9734.23) | 53901.83 (34904.62 to 76274.63) | 31 (19.9 to 45.59) |
| Oceania | 810995.73 (665085.9 to 982278.11) | 16565.41 (12002.32 to 23108.83) | 810995.73 (665085.9 to 982278.11) | 16565.41 (12002.32 to 23108.83) | 2958.89 (1788.69 to 4427.45) | 59.44 (33.64 to 96.43) |
| South Asia | 47190262.15 (36397675.23 to 60576337.86) | 9367.51 (6183.13 to 14292.66) | 47190262.15 (36397675.23 to 60576337.86) | 9367.51 (6183.13 to 14292.66) | 275135.06 (177167.21 to 399305.17) | 54.9 (32.47 to 86.73) |
| Southeast Asia | 16829093.5 (14552056.19 to 19371437.97) | 10207.99 (7904.14 to 12991.69) | 16829093.5 (14552056.19 to 19371437.97) | 10207.99 (7904.14 to 12991.69) | 53962.29 (34714.04 to 80106.16) | 32.89 (20.53 to 50.24) |
| Southern Latin America | 1318910.89 (1043742.92 to 1656819.07) | 8912.02 (6052.8 to 12984.18) | 1318910.89 (1043742.92 to 1656819.07) | 8912.02 (6052.8 to 12984.18) | 2134.29 (1190.04 to 3581.37) | 14.8 (6.64 to 29.78) |
| Southern Sub-Saharan Africa | 3019773.83 (2577546.66 to 3510457.27) | 12827.88 (9636.23 to 16726.84) | 3019773.83 (2577546.66 to 3510457.27) | 12827.88 (9636.23 to 16726.84) | 10439.3 (6852.76 to 15397.26) | 44.34 (27.31 to 68.52) |
| Tropical Latin America | 5376433.13 (3962216.02 to 7183737.08) | 10847.27 (6407.41 to 17109.65) | 5376433.13 (3962216.02 to 7183737.08) | 10847.27 (6407.41 to 17109.65) | 14200.19 (8436.74 to 21425.06) | 29 (15.86 to 48.95) |
| Western Europe | 915104.65 (816945.87 to 1034554.9) | 1355.81 (1108.22 to 1665.44) | 915104.65 (816945.87 to 1034554.9) | 1355.81 (1108.22 to 1665.44) | 672.62 (389.31 to 1102.98) | 1.02 (0.54 to 1.75) |
| Western Sub-Saharan Africa | 37614980.81 (35202347.96 to 40181525.82) | 18803.86 (16846.05 to 21093.73) | 37614980.81 (35202347.96 to 40181525.82) | 18803.86 (16846.05 to 21093.73) | 236538.18 (161434.73 to 332228.05) | 117.81 (79.11 to 167.53) |

**Supplementary Table 10** Global and regional burden of other nutritional deficiencies among children in 2019

| Locations | Prevalent Cases (95%UI) | ASPR/100,000  (95% CI) | Incident cases (95%UI) | ASIR/100,000  (95% CI) | DALY cases | Age-standardized DALY rate |
| --- | --- | --- | --- | --- | --- | --- |
| Global | NA | NA | NA | NA | 611673.96 (475532.06 to 782402.44) | 31.68 (23.6 to 42.19) |
| SDI regions |  |  |  |  |  |  |
| High SDI | NA | NA | NA | NA | 8473.77 (5134.27 to 13050.73) | 5.04 (3.06 to 7.78) |
| High-middle SDI | NA | NA | NA | NA | 27402.62 (19890.25 to 36935.28) | 11.23 (7.9 to 15.42) |
| Middle SDI | NA | NA | NA | NA | 90489.52 (70227.53 to 115570.21) | 16.62 (12.57 to 21.83) |
| Low-middle SDI | NA | NA | NA | NA | 262273.77 (199883.05 to 336364.98) | 51.39 (35.91 to 71.69) |
| Low SDI | NA | NA | NA | NA | 222875.65 (162613.51 to 303058.72) | 46.06 (32.59 to 64.64) |
| GBD regions |  |  |  |  |  |  |
| Andean Latin America | NA | NA | NA | NA | 2334.13 (1606.66 to 3347.13) | 12.87 (8.09 to 19.45) |
| Australasia | NA | NA | NA | NA | 134.95 (87.39 to 209.73) | 2.43 (1.44 to 3.95) |
| Caribbean | NA | NA | NA | NA | 1568.65 (951.01 to 2411.82) | 13.66 (7.19 to 23.51) |
| Central Asia | NA | NA | NA | NA | 3130.69 (2348.33 to 4134) | 11.5 (8.17 to 16.24) |
| Central Europe | NA | NA | NA | NA | 907.95 (592.68 to 1313.96) | 5.1 (3.26 to 7.55) |
| Central Latin America | NA | NA | NA | NA | 5619.22 (4258.74 to 7210.87) | 8.74 (6.13 to 12.17) |
| Central Sub-Saharan Africa | NA | NA | NA | NA | 6637.9 (4157.98 to 10306.23) | 11.29 (6.74 to 18.3) |
| East Asia | NA | NA | NA | NA | 30665.46 (24077.19 to 39137.44) | 13.01 (9.35 to 18.16) |
| Eastern Europe | NA | NA | NA | NA | 2772.55 (1954.69 to 3826.11) | 7.48 (5.03 to 10.76) |
| Eastern Sub-Saharan Africa | NA | NA | NA | NA | 27794.17 (18986.94 to 37633.54) | 15.29 (10.2 to 21.29) |
| High-income Asia Pacific | NA | NA | NA | NA | 715.9 (463.73 to 1064.9) | 3 (1.93 to 4.48) |
| High-income North America | NA | NA | NA | NA | 2483.23 (1497.79 to 3892.21) | 3.62 (2.17 to 5.66) |
| North Africa and Middle East | NA | NA | NA | NA | 19726.22 (14680.58 to 25563.22) | 11.31 (7.99 to 15.51) |
| Oceania | NA | NA | NA | NA | 354.72 (238 to 497.31) | 6.89 (4.46 to 10.09) |
| South Asia | NA | NA | NA | NA | 384828.71 (286844.01 to 505203.79) | 77.57 (54.61 to 108.63) |
| Southeast Asia | NA | NA | NA | NA | 30902.36 (23499.57 to 39929.38) | 18.91 (14.02 to 25.45) |
| Southern Latin America | NA | NA | NA | NA | 379.96 (271.93 to 540.88) | 2.55 (1.63 to 3.93) |
| Southern Sub-Saharan Africa | NA | NA | NA | NA | 1555.6 (1151.78 to 2044.82) | 6.63 (4.7 to 9.19) |
| Tropical Latin America | NA | NA | NA | NA | 4907.48 (3778.21 to 6274.24) | 10.33 (7.5 to 14.08) |
| Western Europe | NA | NA | NA | NA | 5531.93 (3259.89 to 8625.85) | 7.74 (4.52 to 12.17) |
| Western Sub-Saharan Africa | NA | NA | NA | NA | 78722.18 (55389.46 to 108915.43) | 38 (25.89 to 53.26) |
